# A Simple Entry to the 5,8-Disubstituted Indolizidine Skeleton via Hetero Diels-Alder Reaction

**DOI:** 10.3390/molecules28217316

**Published:** 2023-10-28

**Authors:** Juan Francisco Rodríguez-Caro, María M. Afonso, José Antonio Palenzuela

**Affiliations:** Departamento de Química Orgánica, Instituto Universitario de Bio-Orgánica Antonio González (SINTESTER), Universidad de La Laguna, Avda. Astrofísico Fco. Sánchez 2, 38206 La Laguna, Spain; jfrodcaro@gmail.com

**Keywords:** 5,8-indolizidine, hetero Diels–Alder, Δ^1^-pyrroline, indolizidine **181B**

## Abstract

The 5,8-disubstituted indolizidines are the largest family of indolizidines isolated from the skin of amphibians. These compounds exhibit interesting biological activities such as noncompetitive blockers of nicotinic receptors. In this paper, we present a short, simple, and general synthesis of these alkaloids based on the hetero Diels–Alder reaction between suitable monoactivated dienes and Δ^1^-pyrroline as the dienophile. The selectivity of the process is explained based on computational studies. Concise synthesis of the indolizidine alkaloid **181B** from a hetero Diels–Alder reaction was accomplished in four steps.

## 1. Introduction

**Figure 1:**
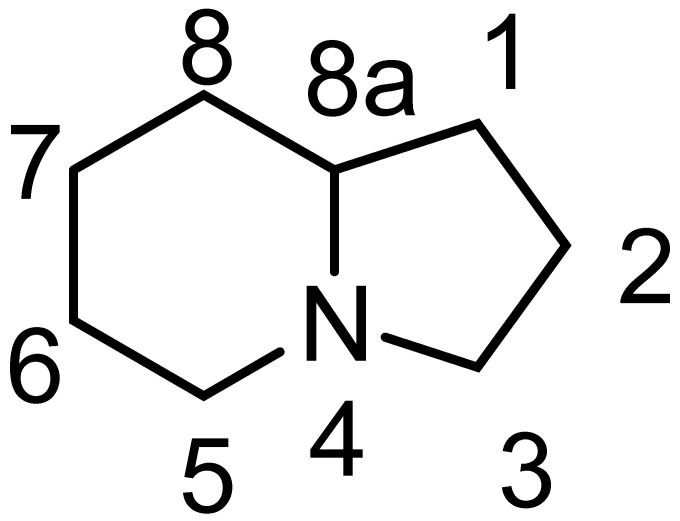
Structure and numbering of the indolizidine skeleton.

Indolizidine alkaloids constitute a large family of heterocyclic compounds isolated from numerous sources, both terrestrial and marine [1,2,3]. The basic structure of indolizidines is that of a five-membered ring fused with a six-membered ring, with a nitrogen atom at one of the fusion points (Figure 1).

**Figure 2:**
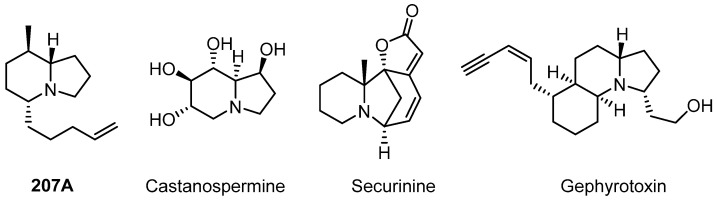
Examples of naturally occurring indolizidines.

Indolizidines exist with different substitution patterns—from the simple basic skeleton with some hydroxyl groups to more complex structures (Figure 2).

Some of these alkaloids show interesting biological activities, such as antitumor, antiviral, antileukemic, and others [4,5].

A large number of indolizidines have been isolated from or detected on the skins of amphibians, and these are usually substituted with alkyl and hydroxyl groups, composing several families of alkaloids [6]. The largest family is that of the 5,8-disubstituted indolizidines, with more than 80 examples reported. These indolizidines come mainly from dietary sources since many have been isolated from mites that coexist with the amphibian [7,8]. Many of these have tentative structures due to the difficulty of isolation because of the small amount present in samples obtained from natural sources.

Although some species of amphibians that contain these alkaloids are known to be toxic, the toxicity is not produced by the 5,8-disubstituted indolizidines. Their reported biological activity is as noncompetitive blockers of nicotinic receptors [9,10].

Numerous synthetic schemes aimed at the preparation of the indolizidine skeleton, both racemic and enantioselective, have been published. The most usual methodology is the cyclization of a suitable monocyclic intermediate, giving the bicyclic skeleton of the alkaloids [11].

**Figure 3:**
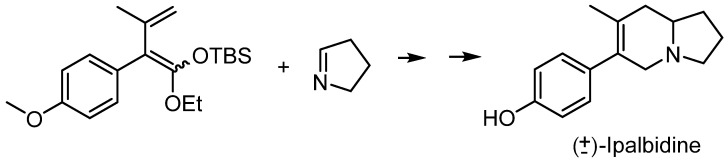
Danishefsky and Vogel’s synthesis of Ipalbidine.

A special mention must be made to Danishefsky and Vogel’s synthesis of (±)-Ipalbidine [12], in which a hetero Diels–Alder reaction between an activated diene and Δ^1^-pyrroline was used as the key step to prepare the target compound (Figure 3).

This approach has been used in a few examples, with Δ^1^-pyrroline derivatives and 1,3-diactivated dienes giving hydroxyl-substituted indolizidines [13,14,15,16], or Δ^1^-pyrroline and allenyltrimethylsilylthioketenes acting as dienes [17,18].

**Scheme 1:**
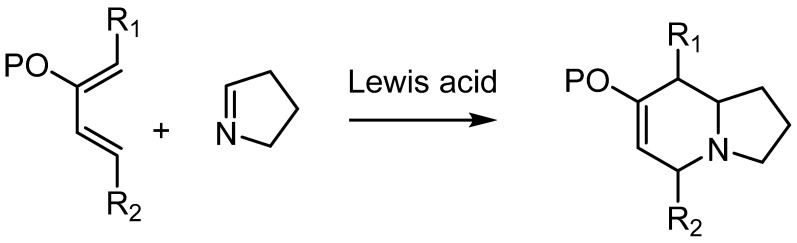
Proposed route to the 5,8-disubstituted indolizidine skeleton.

In our group, the hetero Diels–Alder reactions of monoactivated dienes have been used for the synthesis of oxygen-containing compounds via reaction with aldehydes [19]. We found that monoactivated dienes work well in those reactions in the presence of Lewis acids. Thus, we envisioned that the reaction between a suitable monoactivated diene and a cyclic imine (Δ^1^-pyrroline) could give quick access to the 5,8-alkyl-disubstituted indolizidine skeleton (Scheme 1), providing a simple method to prepare a variety of heterocyclic compounds in a few steps. The relative stereochemistry of the cycloadduct depends on the endo/exo selectivity of the cycloaddition, which in turn depends on the nature of the substituents on the diene. This aspect must be taken into account to match the relative stereochemistry of the natural compounds.

In this article, we present our results in that direction, confirming the idea that the indolizidine skeleton can be accessed from easily prepared dienes and Δ^1^-pyrroline in a few steps. First, the conditions for the reactions between simple monoactivated dienes and Δ^1^ pyrroline were studied. Then stereochemical issues were addressed, and finally, the racemic synthesis of the 5,8-disubstituted indolizidine **181B** from the hetero Diels–Alder reaction was achieved in only four steps.

## 2. Results and Discussion

To simplify the synthesis of the indolizidine skeleton, we decided to use monoactivated dienes with a silyloxy group, which works well with hetero dienophiles such as aldehydes under Lewis acid catalysis [19].

### 2.1. Synthesis of the First Model: Mono-Substituted Indolizidine Skeleton

**Scheme 2:**
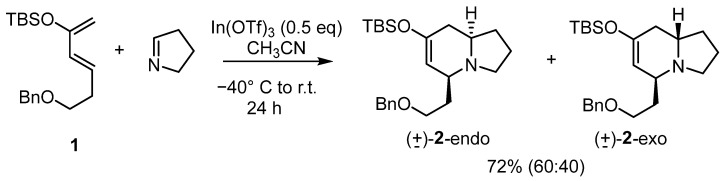
Synthesis of mono-substituted alkyl indolizidine skeleton.

To test this idea, we decided to start with a simple mono-substituted alkyl indolizidine skeleton following the reactions shown in Scheme 2. Mono-substituted indolizidines with the substituent at position five were proposed as natural compounds; however, it was later determined that their structures were incorrect [6]. This type of 5-substituted indolizidine has been the target of several synthesis efforts [20,21,22,23,24,25].

The simple monoactivated diene **1** was chosen to test the reaction. The alkyl chain on **1** contains a benzyloxy group since that group can serve as a reactivity point to make further transformations on the indolizidine skeleton [26,27]. Additionally, these dienes tend to be volatile and somewhat difficult to work with. The presence of the benzyloxy group was expected to reduce the volatility.

Although the hetero Diels–Alder reaction was expected to proceed with high regioselectivity, the endo/exo ratio depends on the structure and degree of substitution on the diene, thus a complete study of the selectivity of the cycloaddition reaction was planned.

The diene was prepared following previous reports [28]. The dienophile Δ^1^-pyrroline was prepared from the diethylacetal of 4-aminobutyraldehyde as described in the literature [29] and was freshly distilled before each use.

We tested BF_3_·OEt_2_, and In(OTf)_3_ as Lewis acids since in our experience these two acids work well in hetero Diels–Alder reactions. The boron derivative gave the expected cycloadducts (±)-**2** in a 40% yield and a 60:40 ratio of endo:exo isomers. The low yield was due to its reactivity with the diene, resulting in recovered unsaturated ketones. With the indium compound, the yield increased to 72% with the same endo:exo ratio. It must be noted that all compounds were slightly volatile even with the benzyloxy group present. The amount of BF_3_·OEt_2_ needed for the reaction to proceed to completion was one equivalent, as is usual for this reagent, since the complex it forms with the dienophile is quite strong. As a comparison, three equivalents were needed for the Danishefsky and Vogel hetero Diels–Alder reaction [12]. For the In(OTf)_3_, it was found that 0.5 equivalents were needed for the reaction to be complete.

The reaction was also studied computationally using the DFT level [30]. These studies concluded that the mechanism is a cycloaddition reaction but with some asynchronicity. The low endo:exo selectivity correlated with the small difference in the energy of the corresponding transition states probably due to the low steric hindrance shown by the diene (for a discussion see the supporting information).

After extraction and purification, the structure and relative stereochemistry of the cycloadducts were studied using standard NMR experiments such as COSY, HMQC, HMBC, and ROESY.

**Figure 4:**
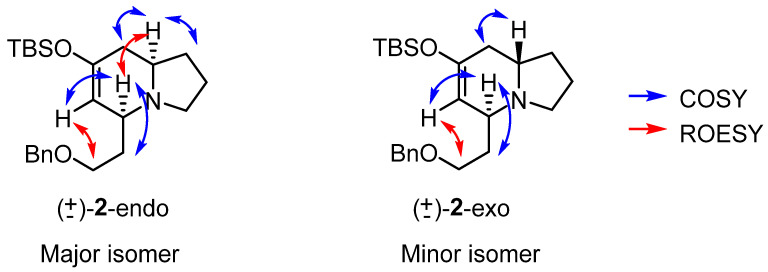
COSY and ROESY correlations for *(±)*-**2**-endo and (±)-**2**-exo.

Figure 4 shows the results of these experiments, which allowed us to ascertain that the major compound obtained in the cycloaddition was the endo isomer. This isomer has the same relative stereochemistry as most of the known mono-substituted indolizidines.

### 2.2. Synthesis of 8-Methyl-5-Alkyl Indolizidine Skeleton

**Scheme 3:**
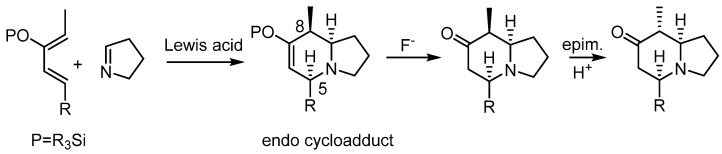
Proposed synthesis of the 8-methyl-5-alkyl indolizidine skeleton with the most common relative stereochemistry found in natural compounds.

Once we found a set of conditions to carry out the hetero Diels–Alder reaction with Δ^1^-pyrroline as the dienophile, we moved on to a system closely related to the most abundant indolizidine alkaloids from amphibious sources, the 5,8-disubstituted indolizidines [6]. Several procedures for the syntheses of this family of indolizidines have been published [31,32,33]. The most common among these are the indolizidines with a methyl group at the eighth position, and thus, we decided to prepare an example of a 5-alkyl-8-methyl indolizidine. In these compounds, the stereochemistry of the stereogenic centers is usually cis for the hydrogens at the positions α to the nitrogen (C5 y C8a) and trans between these and the hydrogen at C8. This relative stereochemistry is expected from the endo adduct of a cycloaddition reaction in which the group at the eighth position has undergone epimerization. Since this position is adjacent to the silyloxy group in the expected cycloadducts, we envisioned that after cleaving the silyl group, the resulting carbonyl group could help to epimerize this position (Scheme 3). A computational study indicated that the epimerized ketone should be more stable than the ketone one coming from the cycloaddition reaction. For a discussion see the supporting information.

**Scheme 4:**
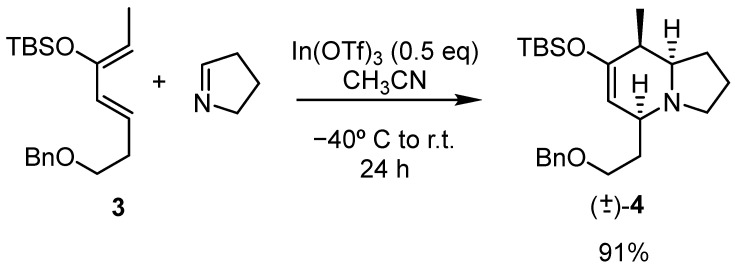
Synthesis of cycloadduct (±)-**4**-endo.

For this study, we decided to maintain the benzyloxy group in the alkyl chain to increase its polarity and facilitate its handling (Scheme 4).

**Figure 5:**
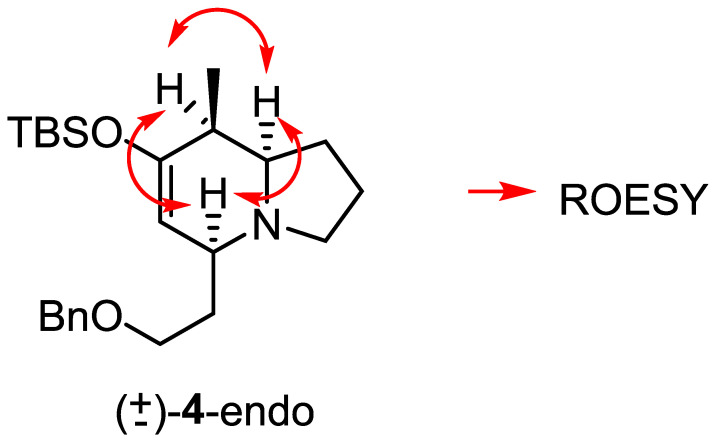
ROESY correlations with the cycloadduct (±)-**4**-endo.

The diene needed for this synthesis was prepared according to the literature [28]. After reacting with Δ^1^-pyrroline under In(OTf)_3_ catalysis, one cycloadduct (±)-**4** was observed as the major compound (>90%) in an almost quantitative yield. NMR studies of this major cycloadduct (Figure 5) indicated that this compound corresponds to the endo adduct and thus, the three hydrogen atoms in the stereocenters of the molecule are in an all-cis relationship. The computational study of this reaction indicates a larger difference in energy between the respective transition states, favoring the endo one (see the supporting information).

**Scheme 5:**
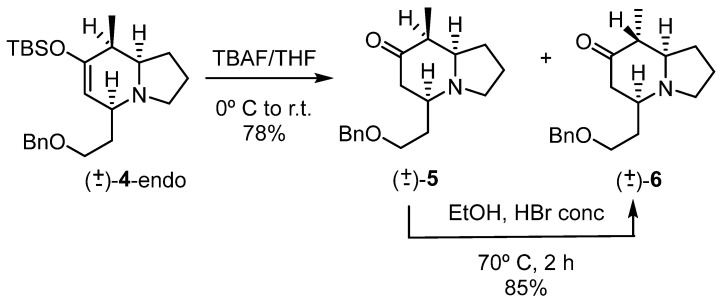
Desilylation and epimerization reactions.

The subsequent step was the cleavage of the enol-silyl group to give the corresponding ketone, which was used to attempt the epimerization of the methyl group on carbon 8 (Scheme 5).

Thus, compound (±)-**4**-endo was treated with TBAF, yielding a mixture of compounds that were identified as two ketones, (±)-**5** and (±)-**6**, epimeric at carbon 8. This result indicated the ease of the epimerization step.

The mixture was treated with HBr [34] to give the completely isomerized ketone (±)-**6**. The yield from these two steps, desilylation–epimerization, was 66%.

**Figure 6:**
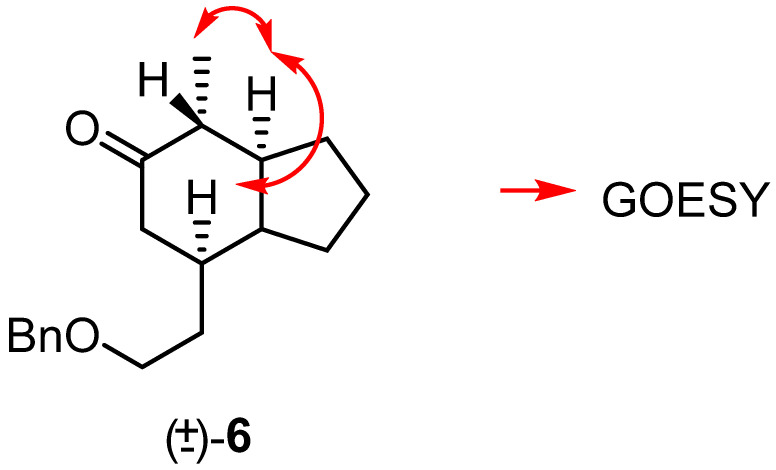
Spatial correlations found for (±)-**6** from GOESY experiments.

NMR spectroscopic analysis indicated that the new keto-indolizidine (±)-**6** had the same relative stereochemistry as most of the naturally occurring 5,8-disubstituted indolizidines (Figure 6).

### 2.3. Synthesis of Indolizidine Rac-**181B**

**Scheme 6:**
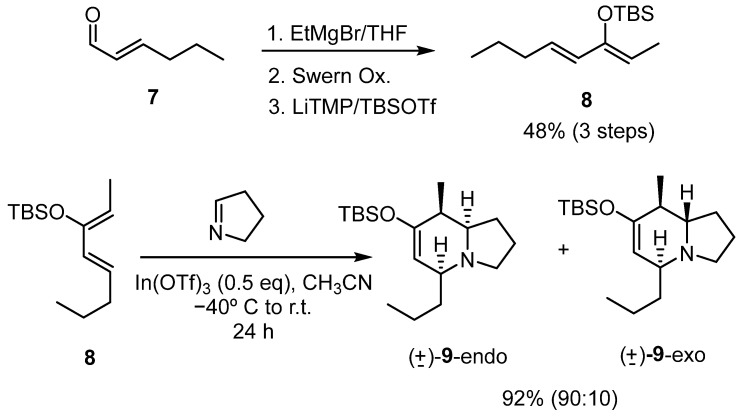
Synthesis of cycloadducts (±)-**9**.

Finally, once the validity of the basic strategy was confirmed, we carried out total synthesis of one natural indolizidine in racemic form. The synthesis could have been performed from the previously obtained keto-indolizidine (±)-**6**, eliminating the ketone group and transforming the benzyloxy group into a leaving group to introduce the required alkyl chain. However, we felt that it would be interesting to prepare the target molecule in as few steps as possible to check the validity of this fast approach to the 5, 8-disubstituted indolizidine family. Thus, a diene with the required substituents in place was needed. Diene **8** was prepared as shown in Scheme 6 as a 9:1 mixture of Z:E isomers. The major isomer, easily separated by column chromatography using hexanes with 1% of Et_3_N as eluent, was reacted with Δ^1^-pyrroline in the presence of In(OTf)_3_ to give the expected cycloadducts (±)-**9** in a 90:10 endo:exo ratio. This ratio is expected from the presence of the methyl group at the eighth position, which increases the selectivity favoring the endo approach as found from the calculation on compound **4**. The yield was almost quantitative as judged by the ^1^H-NMR spectra of the crude reaction mixture, although the isolated yield was 92%, probably due to some decomposition of compounds (±)-**9** in contact with the silica gel.

The structures and relative stereochemistry of both cycloadducts were studied using NMR experiments, which confirmed that the major compound was the endo cycloadduct.

Based on the experience acquired, we decided to treat the endo cycloadduct directly with HBr to obtain the epimerized ketone in one step.

**Scheme 7:**
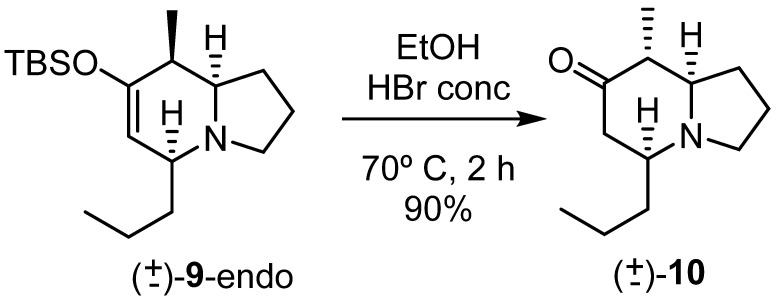
Synthesis of keto-indolizidine (±)-**10**.

When the crude reaction mixture from the cycloaddition reaction was treated directly with HBr under the same conditions as before, ketone (±)-**10** was obtained in a 90% isolated yield (Scheme 7).

The structure was confirmed using NMR analysis. The relative stereochemistry was more difficult to prove since all three hydrogen atoms on the tertiary carbons present similar chemical shifts. Thus the assignment was tentatively established as shown in Scheme 7 based on similarity with compound **6** and some correlations observed in the ROESY spectrum.

The next step was the removal of the carbonyl group. In the literature, there are several examples of this transformation in similar compounds, and we tried some of them. The transformation of the ketone into a dithioketal and reduction with Ni-Raney/H_2_ [35], or treatment with TCDI and radical reduction of the thioester formed [36] gave complex mixtures.

**Scheme 8:**
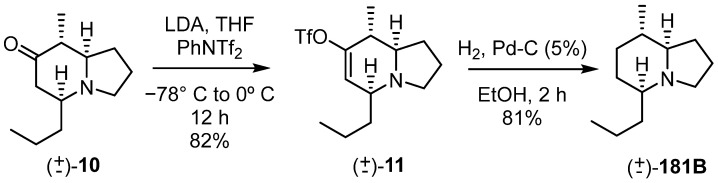
Synthesis of indolizidine (±)-**181B**.

We then resorted to the transformation of the ketone into an enol-triflate and the reduction of this moiety via catalytic hydrogenation (Scheme 8) [37].

The enol-triflate was prepared using LDA as the base and N-phenyl bis-trifluoromethanesulfonimide (PhNTf_2_), giving (±)-**11** in an 82% yield. The hydrogenation step, using Pd/C as the catalyst, gave the reduced compound (±)-**181B** in an 81% yield. The ^1^H NMR spectrum of this compound, after forming its salt with trifluoroacetic acid, was compared to that published by Schneider et al. [38]; the two were found to be identical. Thus, the relative stereochemistry assumed previously was correct and it was demonstrated that a 5,8-indolizidine such as **181B** can be prepared in a very short synthetic sequence, namely, a hetero Diels–Alder reaction, desilylation–isomerization, formation of the enol-triflate, and catalytic hydrogenation in an overall yield of 49.5%.

The different substitutions in the indolizidine skeleton can be introduced by selecting the adequate diene.

This approach can also be used to confirm the proposed structures of the alkaloids isolated or detected in very small amounts from natural sources.

## 3. Materials and Methods

### 3.1. General Experimental Procedures

All moisture-sensitive reactions were carried out under an argon or nitrogen atmosphere with dry solvents under anhydrous conditions. All solvents and reagents were purified using standard techniques or used as supplied from commercial sources. Reactions under standard conditions were monitored by thin-layer chromatography (TLC) on silica gel 60 F254 plates. Visualization was accomplished with UV light, stained with an ethanolic solution of phosphomolybdic acid or ninhydrin, and developed by heating. Silica gel (200–300 mesh) was used for column chromatography. NMR and spectra were recorded in CDCl_3_, C_6_D_6_, or CD_3_OD at 500 MHz for ^1^H NMR and 125 MHz for ^13^C NMR on a Bruker Avance instrument. Chemical shifts are given in (δ) parts per million and coupling constants (J) in Hz. 1H- and 13C-spectra were referenced using the solvent signal as an internal standard. The data are reported as (s = singlet, d = doublet, t = triplet, q = quartet, quintet = quintet, m = multiplet, dd = doublet of doublets, dt = doublet of triplets, and bs = broad singlet). High-resolution mass spectral analysis (HRMS) data were obtained using a VG AutoSpec spectrometer via electrospray ionization (ESI) or electron impact (EI). The ^1^H NMR and ^13^C NMR spectra and two-dimensional NMR spectroscopy of new compounds are provided in the Supplementary Materials.

### 3.2. Compound Synthesis

General Procedure for the Hetero Diels–Alder reaction with In(OTf)_3_

To a solution of In(OTf)_3_ (0.50 mmol) in dry acetonitrile was added a solution of Δ^1^-pyrroline (1.00 mmol) in acetonitrile at 0 °C under argon. After 10 min, the mixture was cooled at −40 °C and a solution of diene (1.00 mmol) in acetonitrile was added. The reaction mixture was allowed to slowly warm to room temperature and stirred for 24 h, quenched with NaHCO_3_ aqueous solution, extracted with ethyl acetate, washed with brine, dried over Na_2_SO_4_, filtered, and concentrated in vacuo. The residue was purified by flash chromatography to give the corresponding products.

Hetero Diels–Alder reaction between diene **1** and Δ^1^-pyrroline

According to the general procedure, a 60:40 mixture of isomers **2-endo:2-exo** was obtained (72%) and separated by flash chromatography (hexane/ethyl acetate 60:40 to 30:70 + TEA 0.2%)

rac-(5S,8aR)-5-(2-(benzyloxy)ethyl)-7-((tert-butyldimethylsilyl)oxy)-1,2,3,5,8,8a-hexahydroindolizine (**2-endo**)

Colorless oil. ^1^H NMR (CDCl_3_, 500 MHz) δ 0.11 (s, 6H), 0.91 (s, 9H), 1.48 (m, 1H), 1.70–2.18 (m, 8H), 2.35 (m, 1H), 2.89 (bs, 1H), 3.32 (t, *J* = 9 Hz, 1H), 3.58 (m, 2H), 4.52 (q, *J* = 12 Hz, 2H), 4.80 (s, 1H), 7.25–7.37 (m, 5H); ^13^C NMR (CDCl_3_, 125 MHz) δ −4.5, −4.2, 18.1, 21.7, 25.7, 30.7, 34.6, 37.1, 52.0, 59.0, 61.4, 67.5, 73.1, 106.6, 127.5, 127.6, 128.3, 138.6, 150.0; HRMS (ESI): Calcd for C_23_H_38_NO_2_Si [M + H] 388.2672, found 388.2671.

rac-(5S,8aS)-5-(2-(benzyloxy)ethyl)-7-((tert-butyldimethylsilyl)oxy)-1,2,3,5,8,8a-hexahydroindolizine (**2-exo**)

Colorless oil. ^1^H NMR (CDCl_3_, 500 MHz) δ 0.13 (s, 6H), 0.91 (s, 9H), 1.59 (m, 1H), 1.69 (m, 1H), 1.91 (m, 1H), 2.05 (m, 1H), 2.11 (m, 2H), 2.87 (m, 1H), 3.00 (m, 1H), 3.26 (m, 1H), 3.54 (m, 2H), 3.62 (m, 2H), 3.67 (m, 1H), 4.51 (q, *J* = 12.5 Hz, 2H), 4.87 (d, *J* = 4.3 Hz, 1H), 7.27–7.38 (m, 5H); ^13^C NMR (CDCl_3_, 125 MHz) δ −4.5, −4.4, 18.0, 21.2, 25.7, 30.3, 32.9, 33.2, 49.4, 53.0, 67.5, 73.1, 127.6, 127.7, 128.4, 138.4, 148.4; HRMS (ESI): Calcd for C_23_H_38_NO_2_Si [M + H] 388.2672, found 388.2666.

Hetero Diels–Alder reaction between diene **3** and Δ^1^-pyrroline

According to the general procedure, compound **4** was isolated (91%) as a major isomer using hexane/ethyl acetate 70:30 + TEA 0.2%

rac-(5S,8S,8aR)5-(2-(benzyloxy)ethyl)-7-((tert-butyldimethylsilyl)oxy)-8-methyl-1,2,3,5,8,8a-hexahydroindolizine (**4**)

Colorless oil. ^1^H NMR (CDCl_3_, 500 MHz) δ 0.00 (s, 3H), 0.02 (s, 3H), 0.81 (s, 9H), 0.89 (d, *J* = 6.7 Hz, 3H), 1.51–1.57 (m, 2H), 1.59–1.76 (m, 4H), 1.90–1.95 (m, 2H), 2.36 (td, *J* = 2.3, 8.5 Hz, 1H), 2.74 (bs, 1H), 3.15 (td, *J* = 8.4, 11.5 Hz, 1H), 3.40–3.45 (m, 1H), 3.47–3.53 (m, 1H), 4.38 (m, 2H), 4.53 (d, *J* = 1.8, 1H), 7.19 (m, 5H); ^13^C NMR (CDCl_3_, 125 MHz) δ −0.46, −0.41, 12.5, 18.0, 22.2, 25.9, 26.0, 34.5, 37.4, 52.3, 59.3, 64.0, 67.4, 73.0, 105.0, 127.4, 127.6, 128.3, 138.5, 155.0; HRMS (EI): Calcd for C_24_H_39_NO_2_Si [M+] 401.2750, found 401.2745.

Synthesis of Keto-indolizidine **6**

To a solution of **4** (0.250 g, 0.62 mmol) in THF (5 mL) at 0 °C was added tetrabutylammonium fluoride trihydrate (0.196 g, 0.62 mmol). The mixture was allowed to reach room temperature and after completion, the solvent was removed by vacuum, affording a 1:2 mixture of C-8 methyl-epimers **5** and **6** (0.139 g, 78%). To a solution of this mixture in EtOH (3 mL) was added HBr (conc.) (1.0 mL). The reaction mixture was heated at 70 °C for 2 h. After cooling to room temperature, the solvent was removed by vacuum, and DCM was added. Then, an aqueous solution of NaHCO_3_ was added, extracted with DCM, washed with brine, dried over Na_2_SO_4_, filtered, and concentrated in vacuo. The residue was purified by flash chromatography hexane/ethyl acetate 35:65, affording **6** as the only compound (0.118 g, 85%)

rac-(5S,8R,8aR)-5-(2-(benzyloxy)ethyl)-8-methylhexahydroindolizin-7(1H)-one (**6**)

Yellow oil. ^1^H NMR (CDCl_3_, 500 MHz): 1.00 (d, *J* = 6.7, 3H), 1.60 (m, 1H), 1.71 (m, 1H), 1.79 (m, 1H), 1.90 (m, 1H), 2.00 (m, 2H), 2.12 (m, 2H), 2.32 (m, 2H), 2.43 (m, 1H), 2.52 (m, 1H), 3.27 (t, *J* = 9.0 Hz, 1H), 3.51 (m, 2H), 4.48 (s, 2H), 7.25–7.37 (m, 5H) ^13^C NMR (δ, CDCl_3_): 10.5, 21.4, 30.0, 34.8, 46.0, 50.2, 50.8, 59.2, 66.6, 70.4, 73.1, 127.6, 128.0, 138.4, 210.5; HRMS (EI): Calcd for C_18_H_25_NO_2_ 287.1885, found 287.1878.

Synthesis of tert-butyldimethyl(((2Z,4E)-octa-2,4-dien-3-yl)oxy)silane (**8**)

To an ethylmagnesium bromide solution (1.0 M in THF, 76 mmol, 1.5 equiv) at 0 °C was slowly added trans-2-hexenal (5.0 g, 50.9 mmol, 1.0 equiv) in THF (25 mL). The reaction was allowed to warm to room temperature and stirred for 1 h. The reaction mixture was carefully quenched with saturated aqueous NH_4_Cl, extracted with diethyl ether, washed with brine, dried over MgSO_4_, and carefully concentrated in vacuo. The residue was purified by distillation through a vigreux column, bp. 112 °C (760 Torr), yielding the corresponding alcohol (4.6 g, 71%)

(E)-oct-4-en-3-ol (**8**): Pale yellow oil. ^1^H NMR (CDCl_3_, 500 MHz) 0.80 (t, *J* = 7.3 Hz, 6H), 1.25–1.52 (m, 5H), 1.91 (m, 2H), 3.87 (q, *J* = 6.7 Hz, 1H), 5.35 (dt, *J* = 15.2, 7.0 Hz, 1H), 5.53 (dt, *J* = 15.2, 6.9 Hz, 1H).

A round-bottom flask equipped with a magnetic stirrer and containing DMSO (1.06 mL, 15 mmol) in DCM (100 mL) at −78 °C was charged dropwise with oxalyl chloride 2.0 M in DCM (6 mL, 12 mmol,) and stirred for 30 min. To the resulting mixture, at −60 °C, a solution of the previously prepared alcohol (1.28 g, 10 mmol) in DCM (8 mL) was added dropwise and the reaction mixture was stirred for 1 h at −60 °C, then TEA (6.9 mL, 50 mmol) was added dropwise and stirred for 15 min. The reaction mixture was allowed to warm to room temperature, diluted with DCM, quenched by the addition of water, extracted with DCM, and the combined organic layers washed sequentially with 1% aqueous HCl solution (30 mL), followed by 5% aqueous NaHCO_3_ solution (30 mL), and brine (30 mL). The organic layers were dried (MgSO_4_) and filtered, and the solvent was carefully removed by vacuum. The crude product was purified by flash chromatography hexane/ethyl acetate 85:15, affording (E)-oct-4-en-3-one (1.07 g, 85%).

(E)-oct-4-en-3-one: Colorless oil. ^1^H NMR (CDCl_3_, 500 MHz) 0.94 (t, *J* = 7.7 Hz, 3H), 1.09 (t, *J* = 7.4 Hz, 3H), 1.49 (m, 2H), 2.19 (m, 2H), 2.56 (q, *J* = 7.4 Hz, 2H), 6.09 (dt, *J* = 15.7, 1.5 Hz, 1H), 6.85 (dt, *J* = 15.7, 7.0 Hz, 1H).

To a solution of (E)-oct-4-en-3-one (0.75 g, 5.95 mmol) in THF (20 mL) cooled at −78 °C, tert-butyldimethylsilyl trifluoromethane sulfonate (1.6 mL, 7.14 mmol) was added. Then, 2,2,6,6-tetramethylpiperidine lithium salt (5.95 mmol, 0.3 M in THF) was added. After 15 min, an aqueous solution of NH_4_Cl was added and the mixture was extracted with diethyl ether, washed with brine, dried over Na_2_SO_4_, filtered, and carefully concentrated in vacuo. The combined organic layers were dried over anhydrous Na_2_SO_4_ and the solvent was removed by vacuum. The crude product was purified by flash chromatography hexane + 1% TEA, affording **8** (1.14 g, 80%)

tert-Butyldimethyl(((2Z,4E)-octa-2,4-dien-3-yl)oxy)silane (**8**): Colorless oil. ^1^H NMR (C_6_D_6_, 500 MHz) δ 0.15 (s, 6H), 0.87 (t, *J* = 7.3 Hz, 3H), 1.07 (s, 9H), 1.35 (quintet, *J* = 7.3 Hz, 2H), 1.65 (d, *J* = 6.8 Hz, 3H), 1.99 (q, *J* = 6.9 Hz, 2H), 4.73 (q, *J* = 7.0 Hz, 1H), 5.86–5.97 (m, 2H); ^13^C NMR (C_6_D_6_, 125 MHz) δ −3.4, 11.9, 13.9, 18.7, 23.1, 26.2, 34.8, 128.7, 129.6, 149.7; HRMS (EI) calc for C_14_H_28_OSi 240.1909, found 240.1904.

Hetero Diels–Alder reaction between diene **8** and Δ^1^-pyrroline

According to the general procedure, a mixture of compounds **9-endo:9-exo** was isolated (92%) as 90:10 isomers, hexane/ethyl acetate 70:30 to 50:50 + TEA 0.2%

rac-(5S,8S,8aR)-7-((tert-butyldimethylsilyl)oxy)-8-methyl-5-propyl-1,2,3,5,8,8a-hexahydroindolizine (**9-endo**)

Colorless oil. ^1^H NMR (C_6_D_6_, 500 MHz) δ 0.18 (s, 3H), 0.19 (s, 3H), 0.94 (t, *J* = 7.3 Hz, 3H), 1.00 (s, 9H), 1.24 (d, *J* = 6.8 Hz, 3H), 1.27–1.40 (m, 1H), 1.41–1.51 (m, 2H), 1.52–1.75 (m, 5H), 1.96 (q, *J* = 8.6 Hz, 1H), 2.13 (m, 1H), 2.50 (m, 1H), 2.84 (bs, 1H), 3.17 (t, *J* = 7.4 Hz, 1H), 4.77 (d, *J* = 1.7 Hz, 1H); ^1^H NMR (CDCl_3_, 500 MHz): 0.14 (s, 3H), 0.15 (s, 3H), 0.90 (t, *J* = 7.0 Hz, 3H), 0.93 (s, 9H), 1.02 (d, *J* = 6.8 Hz, 3H), 1.27 (m, 1H), 1.40 (m, 2H), 1.49 (m, 2H), 1.66 (m, 1H), 1.75 (m, 2H), 1.99 (m, 1H), 2,05 (m, 1H), 2.46 (m, 1H), 2.66 (m, 1H), 3.26 (td, *J* = 8.8, 2.1 Hz, 1H), 4.67 (d, *J* = 1.7 Hz, 1H); ^13^C NMR (C_6_D_6_, 125 MHz) δ −4.5, −4.0, 12.9, 14.9, 17.8, 18.3, 22.8, 26.0, 26.3, 37.5, 38.1, 52.3, 61.7, 64.2, 105.7, 155.5; E.M. m/z (relative intensity): 309 (M+) (2.5), 266 (100), 75 (12), 73 (23); HRMS: Calcd for C_18_H_35_NOSi [M+] 309.2488, found 309.2480.

rac-(5S,8S,8aS)-7-((tert-butyldimethylsilyl)oxy)-8-methyl-5-propyl-1,2,3,5,8,8a-hexahydroindolizine (**9-exo**)

Colorless oil. ^1^H NMR (C_6_D_6_, 500 MHz) δ 0.20 (s, 3H), 0.21 (s, 3H), 0.96 (t, *J* = 7.0 Hz, 3H), 1.03 (s, 9H), 1.10 (d, *J* = 6.8 Hz, 3H), 1.44 (m, 2H), 1.58 (m, 1H), 1.69 (q, *J* = 7.3 Hz, 1H), 1.91 (m, 1H), 2.03 (q, *J* = 7.0 Hz, 1H), 2.71 (bs, 1H), 2.78 (t, *J* = 7.0 Hz, 2H), 3.37 (m, 1H), 5.04 (d, *J* = 3.7 Hz, 1H); ^13^C NMR (C_6_D_6_, 125 MHz) δ −4.5, −4.4, 14.8, 15.6, 18.5, 20.5, 22.3, 26.0, 30.3, 36.1, 37.4, 50.1, 55.2, 60.0, 106.1, 152.2.

Synthesis of Keto-indolizidine (**10**)

To a solution of **9-endo** (225 mg, 1.02 mmol) in EtOH (4 mL) was added HBr (conc.) (1.3 mL). The reaction mixture was heated at 70 °C for 2 h. After cooling to room temperature, the solvent was removed by vacuum, and DCM was added. Then, an aqueous solution of NaHCO_3_ was added, extracted with DCM, washed with brine, dried over Na_2_SO_4_, filtered, and concentrated in vacuo. The residue was purified by flash chromatography hexane/ethyl acetate 70:30 to give compound **10** (128 mg, 90%)

rac-(5S,8R,8aR)-8-methyl-5-propylhexahydroindolizidin-7(1H)-one (**10**)

^1^H NMR (C_6_D_6_, 500 MHz) δ 0.78 (t, *J* = 7.1 Hz, 3H), 1.00 (d, *J* = 6.5 Hz, 3H), 1.04 (m, 1H), 1.24 (m, 2H), 1.37 (m, 2H), 1.60 (m, 1H), 1.68 (m, 1H), 1.74 (q, *J* = 8.6 Hz, 1H), 2.09 (m, 2H), 2.31 (d, *J* = 10.5 Hz, 1H), 2.96 (t, *J* = 8.5 Hz, 1H); ^13^C NMR (C_6_D_6_, 125 MHz) δ 10.9, 14.5, 17.9, 21.8, 30.4, 37.3, 45.8, 50.3, 50.6, 61.5, 70.6, 208.6; HRMS (ESI): Calcd for C_12_H_22_NO [M + H] 196.1701, found 196.1696.

Synthesis of Enol-triflate (**11)**

To a solution of LDA (1.58 mmol, 0.5 M in THF) at −78 °C was added, dropwise, a solution of keto-indolizidine **10** (0.103 g, 0.53 mmol) in THF (2 mL). The reaction mixture was stirred and allowed to slowly warm to 0 °C for 2 h, then a solution of N-Phenyl-bis(trifluoromethanesulfonimide (0.284 g, 0.8 mmol) in THF (2 mL) was added, and then stirred at 0 °C for 12 h. The reaction mixture was diluted with diethyl ether, quenched by the addition of water, extracted with diethyl ether, washed with brine, dried over MgSO_4_, and concentrated in vacuo. The residue was purified by flash chromatography column hexane/ethyl acetate 90:10, affording **11** (0.142 g, 82%)

rac-(5S,8S,8aR)-8-methyl-5-propyl-1,2,3,5,8,8a-hexahydroindolizin-7-yl trifluoromethanesulfonate (**11**)

Colorless oil. ^1^H NMR (CDCl_3_, 500 MHz) δ 0.94 (t, *J* = 7.2 Hz, 3H), 1.11 (d, *J* = 7.0 Hz, 3H), 1.30 (m, 2H), 1.47 (m, 1H), 1.61 (m, 2H), 1.82 (m, 1H), 1.94 (m, 1H), 2.10 (m, 1H), 2.18 (m, 2H), 2.55 (m, 1H), 2.93 (m, 1H), 3.35 (m, 1H), 5.67 (s, 1H).

Synthesis of **181B**

A stirred solution of **11** (0.140 g, 0.43 mmol) in EtOH (2 mL) was degassed and purged with vacuum–argon cycles. To this solution was added 28 mg (20% weight) of 5 % Pd-C. The mixture was stirred under a hydrogen atmosphere at balloon pressure for 2 h, and then argon was bubbled through the solution for a few minutes. The reaction mixture was filtered through Celite and then one drop of trifluoroacetic acid was added. The solvent was removed by vacuum to give 103 mg of compound **181B** as its salt (81% yield).

rac-(5S,8S,8aR)-8-methyl-5-propyloctahydroindolizine (**181B**)

^1^H NMR (CD_3_OD, 500 MHz) δ 0.99 (t, *J* = 7.0 Hz, 3H), 1.03 (d, *J* = 6.6 Hz, 3H), 1.24–1.57 (m, 5H), 1.67 (m, 2H), 1.90 (m, 2H), 2.00 (m, 1H), 2.13 (m, 2H), 2.37 (m, 1H), 2.86 (m, 1H), 3.07 (m, 2H), 3.73 (m, 1H); ^13^C NMR (CD_3_OD, 125 MHz) δ 14.1, 18.3, 19.4, 20.0, 28.3, 30.1, 32.5, 35.2, 36.0, 52.1, 65.2, 73.6; HRMS (ESI): Calcd for C_12_H_24_N [M + H] 182.1909, found 182.1905.

## Data Availability

Data is contained within the article or Supplementary Materials.

## Supplementary Materials

The following supporting information can be downloaded at: https://www.mdpi.com/article/10.3390/molecules28217316/s1: ^1^H-NMR, ^13^C-NMR, and 2D spectra of compounds 2-endo, 2-exo, 4-endo, 6, 11-endo, 11-exo, 12, and **181B**; ^1^H-NMR and ^13^C-NMR of compound 10 and ^1^H-NMR of compound 13; Discussion of the calculations of the reaction profiles of dienes 1 and 3 with Δ^1^-pyrroline, epimerization of compounds **5** to **6**, and the cartesian coordinates of the stationary points of computational calculations. Refs. [39,40] are cited in the Supplementary Materials.

## Sample Availability

Not available.
