# Supplementary material for: A Simple Entry to the 5,8-Disubstituted Indolizidine Skeleton via Hetero Diels-Alder Reaction
Source: Molecules. 2023 Oct 28;28(21):7316. doi: 10.3390/molecules28217316 (PMC10647431; doi:10.3390/molecules28217316)
Supplement: Supplementary file 1 [file molecules-28-07316-s001.zip › molecules-2683168-supplementary.pdf]

A simple entry to the 5,8-disubstituted indolizidine skeleton via hetero Diels-Alder reaction.

Juan Francisco Rodríguez-Caro, María del Mar Afonso* and José Antonio Palenzuela,*

Instituto Universitario de Bio-Orgánica Antonio González (SINTESTER), Departamento de Química Orgánica, Universidad de La Laguna, Avda. Astrofísico Fco. Sánchez 2, 38206 La Laguna, Tenerife, Spain

* Correspondence: mmafonso@ull.edu.es (M.d.M.A.); jpalenz@ull.edu.es (J.A.P.).

Supplementary material

Table of contents

Computational section 2

General Computational Procedures 2

Reaction of diene DA 3

Reaction of diene DB 4

Epimerization reaction 5

Coordinates of the calculated stationary points 7

NMR spectra of the synthesized compounds 21

Computational section

General computat onprocedures

All calculations were performed using Orca 5.01 [30]. The functional used was B3LYP with the D4 correction and the def2-SVP basis set. The solvent (THF) was simulated using the Conductor-like Polarizable Continuum Model (CPCM) as implemented in ORCA. The transition states were found using the NEB protocol as implemented in Orca. All stationary states were confirmed by frequency calculations giving 0 imaginary frequencies for starting materials and final products and one imaginary frequency for transition states. That frequency was checked to correspond to the relevant bond formation. IRC analysis was performed to ascertain that the transition states correspond to the reaction studied. No intermediates were found in those analyses. The Lewis acid was modeled using BF_3 coordinated to the nitrogen atom of the dienophile. The structures (Figure S1) were constructed using Avogadro 4.2.1 [39] and the 3D images were made with Cylview 1.06 beta [40].

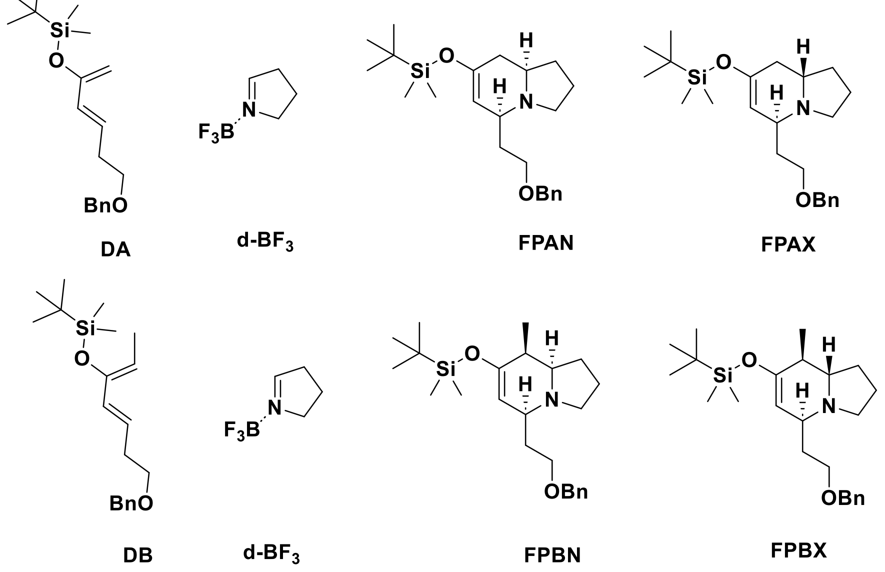

Figure S1. Structure of the compounds used in the calculations.

Reaction of diene DA

Figure S2 shows the relevant points in the reaction profile for the reaction of diene DA in the calculations) and Δ[1]-pyrroline coordinate to BF_3 d-BF_3).

Figure S2. Reaction profile for the reaction of diene X and Δ[1]-pyrroline. Relevant distances are in Å, and activation energies, and reaction energies, at 298 K are in kcal/mol.

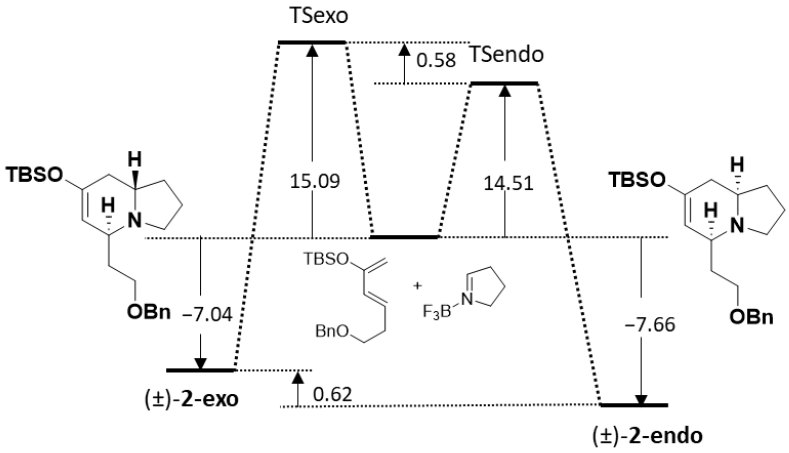

The difference between the two transition states, corresponding to the endo (TSAN) and exo (TSAX) approaches, relative to the starting materials is small, thus suggesting that a mixture close to 1:1 should be obtained. In this case, experimentally, a 60:40 mixture favoring the endo adduct was obtained.

An analysis of the transition states (Figure S3) indicates that the partial bond between the extreme of the diene and the carbon terminus of the double bond on the dienophile is shorter than the other intermolecular bond being formed, thus suggesting an asynchronicity in this reaction with the C-C bond forming faster that the C-N bond. However, no intermediate was found at this level of theory, indicating that this is a pericyclic reaction and not a stepwise one. This is observed in both transition states, corresponding to the endo and exo approaches of the dienophile.

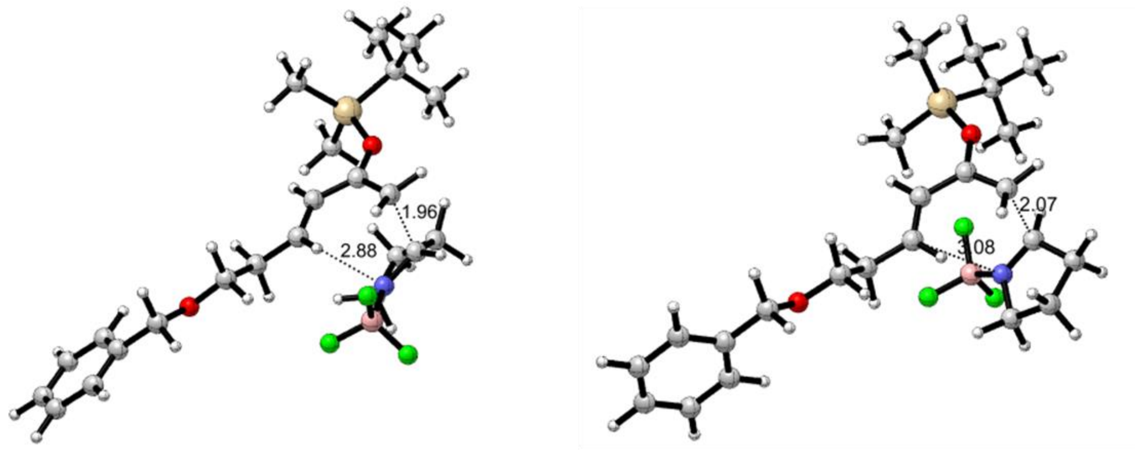

TSAN TSAX

Figure S3. The transition state of the endo (TSAN) and exo (TSBX) approaches of the dienophile to the diene DA with the bond-forming distances. The distances are indicated in Å.

Reaction of diene DB

The analysis of the reaction profile of diene DB, is similar to the already discussed, but the presence of the methyl group increases the energy difference between the two transition states relative to the starting materials, suggesting that the endo approach should be preferred in this reaction. Experimentally, the endo product is obtained in a 90:10 ratio relative to the exo approach (Figure S4).

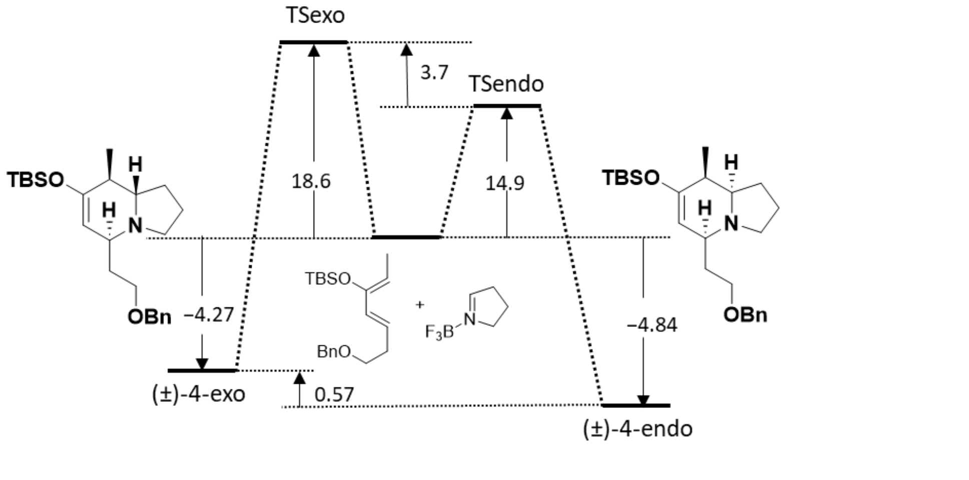

Figure S4. Reaction profile for the reaction of diene DB and Δ^1-pyrroline. Relevant distances are in Å, and activation energies, and reaction energies, at 298 K are in kcal/mol.

The transition states also show a similar behavior as the simpler diene (Figure S5)

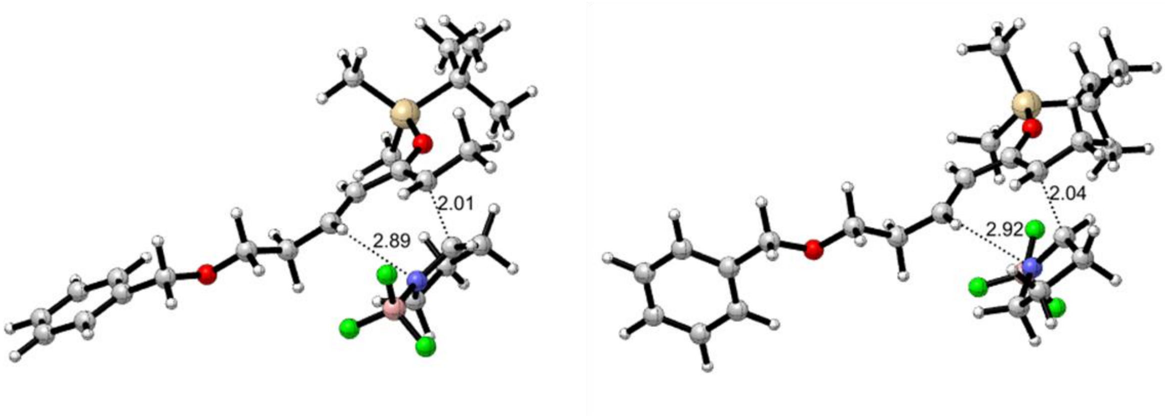

TSBN TSBX

Figure S5 Transition state of the endo (TSBN) and exo (TSBX) approaches of the dienophile to the diene DB with the bond-forming distances. The distances are indicated in Å.

Epimerization reaction

The last aspect analyzed using computational studies was the relative stability of the two epimers on the carbon 8 of the keto-indolizidines KBN and KBN-iso.

The thermodynamic stability of the two epimers was calculated and it was found that the epimerized compound was more stable than the one coming from the cycloaddition reaction by 4.14 kcal/mol. In Figure S6 can be seen that in the epimerized compound, the methyl group adopts an equatorial disposition which results in less steric interactions.

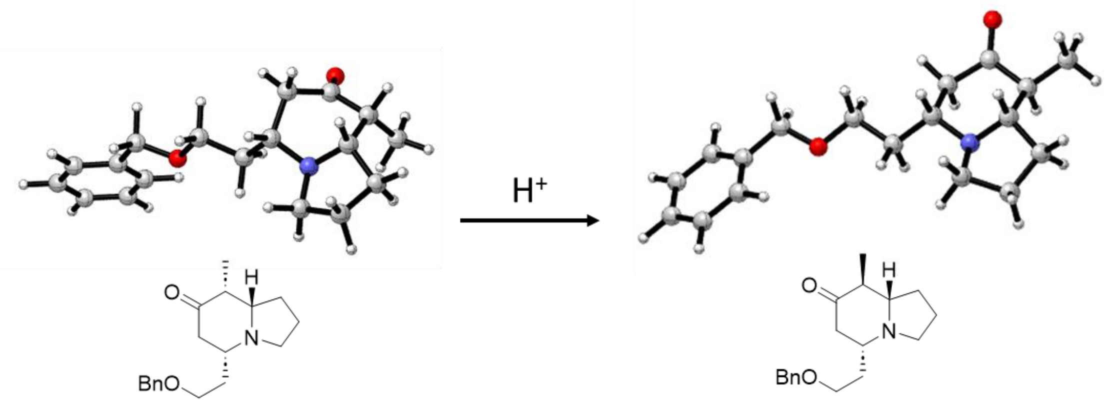

KBN KBN-iso

Figure S6. Structures of the keto-indolizidines showing the conformational changes occurring after the epimerization step.

Coordinates of the calculated stationary points

Δ1-pyrroline + BF_3 (d-BF_3)

Total Enthalpy ... -535.22348855 Eh
Final entropy term (T*S) ... 0.04276581 Eh
Final Gibbs free energy ... -535.26625436 Eh

H	-0.40019730856075	-1.64397710709809	1.65438787421788
C	-0.45186746746517	-1.20954310953144	0.64750357209552
H	-1.18611046668096	-1.78726614011496	0.06632777670210
C	0.91296089987846	-1.21512601991087	-0.07890868521541
H	1.77653091259732	-1.24438691781666	0.61219755827106
H	1.05295583853167	-2.04543228750147	-0.78838095648202
C	0.91685029159946	0.10708036572354	-0.76757677614081
H	1.63619610583808	0.44091887420081	-1.52115399591065
N	-0.01605207236140	0.89163360184961	-0.37772719324454
C	-0.85188207939632	0.27777197114576	0.67350841540034
H	-0.61314570447897	0.78195495605823	1.62386112870730
H	-1.91007784198546	0.46748469775099	0.44733039324250
B	-0.26624591358507	2.38653878768297	-0.88804121506438
F	0.71684403707371	2.66217462332848	-1.82505711206584
F	-0.17948701674005	3.20685903645491	0.22680745804352
F	-1.53824221426454	2.41494466777818	-1.43983824255656

Diene DA

Total Enthalpy ... -1180.32550701 Eh
Final entropy term (T*S) ... 0.08177194 Eh
Final Gibbs free energy ... -1180.40727894 Eh

C	0.85512212444492	2.02875560103913	-1.38985883744628
C	0.13499801565686	-0.72770707172936	-0.15832455156118
C	0.38636918112072	1.76410976871409	-0.15618832116402
C	0.39348325200269	0.42813653406983	0.47307855967186
C	0.15334701397537	-2.07466288028726	0.49886011831204
C	1.25451126658092	-2.97954523715383	-0.04789251821967
O	1.13246737276174	-4.24510792074911	0.56259144636168
C	2.11060539470686	-5.17414418892932	0.15440738936223
C	1.79917845519531	-6.53648049932233	0.72403389950900
C	2.83465794619015	-7.41498961387556	1.07230303652496
C	2.55013413973840	-8.69486275875236	1.55900437814470
C	1.22260952879336	-9.10764308789380	1.71272628824576
C	0.47009837335672	-6.95693903546107	0.88204070878211
C	0.18382398841447	-8.23297643186566	1.37496036012340
O	-0.10061390197107	2.76998799871141	0.62893583451556
Si	-1.64874607348784	2.84927599329782	1.35325877063969
C	-1.66958296211700	1.82777374755490	2.92961972553165
C	-2.89917855337701	2.19432476256202	0.11606158253868
C	-1.86396598219838	4.70494253151575	1.71644632219493
C	-0.71536224224759	5.18220060103021	2.62530396383596

C	-1.83187273249578	5.49216649658176	0.39339722348843
C	-3.21297586604319	4.93683596148603	2.42319819108656
H	1.32145360346526	1.24347972769782	-1.98628374282645
H	-0.10879480768867	-0.70411969037666	-1.22820882895539
H	0.28319424483449	-1.97178447778282	1.58851432190094
H	-0.81138303759850	-2.58674309489918	0.32870031181531
H	1.17000211665169	-3.07209895704629	-1.15090914620315
H	2.24991296542111	-2.53809121265227	0.16306248665856
H	2.13775769159376	-5.23172545516615	-0.95550558535748
H	3.12150691537896	-4.84961200503248	0.47407345208542
H	3.87472131151742	-7.09248208043691	0.96490149754571
H	3.36859090673545	-9.36822134911325	1.82776896639206
H	0.99807555974602	-10.10555978535086	2.09887928848970
H	-0.33905791681947	-6.27118001029139	0.62319051324037
H	-0.85674691024308	-8.54680840639584	1.49635855707646
H	-0.82729965772851	2.09576412135923	3.58752726785528
H	-2.60594636180287	1.99887278632712	3.48609222058409
H	-1.60219997962675	0.75209462188727	2.70365462713616
H	-2.83383546846853	2.73414710994496	-0.84205801044847
H	-2.72060024255395	1.12474201599858	-0.08038905797835
H	-3.92584846903469	2.30143848833856	0.50308643421227
H	0.26765046495786	5.01835150592799	2.15534845970655
H	-0.81293876133094	6.26394260998457	2.83132281529883
H	-0.71590771499613	4.66056258740602	3.59661179829753
H	-0.88719564745697	5.33130377234645	-0.15037325213152
H	-2.65929015718405	5.20422774930214	-0.27578442465213
H	-1.92636364563072	6.57606461075170	0.58898648866200
H	-3.35745669246253	6.01337385779207	2.62815906255960
H	-4.06500705356698	4.60294475703034	1.80769465917108
H	-3.26828390302002	4.40867523560796	3.38915425774166
H	0.79530793538399	3.03926423547223	-1.80117566393685
H	0.59295497252676	0.41770546082586	1.55167665558212

TSAN

Total Enthalpy ... -1715.55367804 Eh
Final entropy term (T*S) ... 0.09672882 Eh
Final Gibbs free energy ... -1715.65040685 Eh
Imaginary mode -429.23 cm**-1

C	1.501700	2.202150	-0.863410
C	0.970190	-0.594060	-0.148200
C	0.469380	1.808770	0.003940
C	0.211910	0.429020	0.319990
C	0.599990	-2.029520	-0.011260
C	1.787890	-2.918640	0.334710
O	1.337070	-4.253530	0.366550
C	2.343740	-5.188540	0.678950
C	1.752920	-6.573150	0.787410
C	2.543470	-7.700200	0.519700
C	2.017060	-8.988220	0.655470
C	0.687020	-9.164600	1.052410

C	0.420650	-6.757130	1.184560
C	-0.109240	-8.044510	1.313770
O	-0.394250	2.740120	0.379270
Si	-1.688760	2.858110	1.526830
C	-1.033200	2.248880	3.172840
C	-3.145810	1.847020	0.920300
C	-2.051980	4.725120	1.514680
C	-0.781800	5.504810	1.906050
C	-2.505780	5.155530	0.105990
C	-3.173500	5.021360	2.531580
H	2.400850	1.587270	-0.898000
H	1.911720	-0.386600	-0.658300
H	-0.702170	0.198330	0.872670
H	-0.210200	-2.166160	0.723200
H	0.218480	-2.367890	-0.992820
H	2.582240	-2.791800	-0.427670
H	2.222170	-2.631170	1.314520
H	3.143460	-5.176450	-0.088900
H	2.830640	-4.914060	1.640150
H	3.580540	-7.567620	0.196680
H	2.645200	-9.857100	0.440840
H	0.271810	-10.170980	1.153040
H	-0.200350	-5.881710	1.382660
H	-1.151090	-8.173520	1.620270
H	-0.121890	2.796320	3.461560
H	-1.789880	2.403790	3.959680
H	-0.795280	1.174100	3.141130
H	-3.444660	2.146030	-0.096510
H	-2.926570	0.768140	0.913100
H	-4.007380	2.009060	1.589410
H	0.045440	5.313410	1.204130
H	-0.985230	6.590770	1.896910
H	-0.433770	5.243150	2.918750
H	-1.736330	4.942770	-0.653790
H	-3.433980	4.646460	-0.200510
H	-2.702920	6.242540	0.086460
H	-3.402970	6.102120	2.538190
H	-4.107300	4.489350	2.285230
H	-2.885810	4.740770	3.558360
C	-1.432530	1.238450	-2.678690
H	-2.356710	1.334200	-3.266180
C	-0.619740	-0.008930	-3.057240
H	-0.760130	-0.271750	-4.122740
H	-0.885440	-0.897940	-2.466170
N	0.777990	0.389030	-2.843590
C	0.901680	1.728380	-2.670850
H	1.799800	2.189640	-3.095660
B	1.961370	-0.511860	-3.258810
F	1.629830	-1.850440	-3.004150
F	3.095200	-0.136920	-2.501970
F	2.243000	-0.348000	-4.622790
H	-1.718830	1.199810	-1.618060
C	-0.442640	2.389740	-2.922420

H	-0.466650	2.711950	-3.978840
H	-0.626620	3.276500	-2.300480
H	1.664430	3.280170	-0.945820

TSAX

Total Enthalpy ... -1715.55086572 Eh
Final entropy term (T*S) ... 0.09861174 Eh
Final Gibbs free energy ... -1715.64947746 Eh
Imaginary mode -333.20 cm**-1

C	1.51584910189007	2.17140115730808	-0.76007194329310
C	0.84033750358397	-0.68171331503008	-0.29684617331852
C	0.64495616499459	1.72790768166661	0.23050374713742
C	0.35737720473373	0.32681643138340	0.45842284849792
C	0.45173304773073	-2.11085080015454	-0.11973188202032
C	1.62714304072674	-2.99693167546316	0.28489118152645
O	1.15847981442248	-4.32224813487828	0.38467182523624
C	2.13123700459830	-5.23983235650981	0.82931256998754
C	1.56000294449549	-6.63658476525589	0.83982634980888
C	2.01185912754262	-7.57795468132003	1.77561529096451
C	1.51582927745079	-8.88520094351334	1.76782644091271
C	0.55166606925410	-9.26423904133053	0.82790012069967
C	0.59295072315546	-7.02342618011898	-0.09953823308095
C	0.09042454316296	-8.32774799101095	-0.10370109241650
O	-0.03441067959485	2.64188120054531	0.89885175280623
Si	-1.34947363131936	2.81794388984965	2.00321201464265
C	-0.52889751309204	3.00925659558374	3.67630105599075
C	-2.52907380463324	1.36360592610832	1.98268420885101
C	-2.18069262600103	4.41786972149694	1.39544468588392
C	-1.12586608117207	5.52811689873925	1.23428041859874
C	-2.86962038819409	4.16708293404522	0.04102837500068
C	-3.23104373045417	4.85437520236160	2.43580727664731
H	2.29553748682896	1.49526104466063	-1.10644555576280
H	1.57301595329558	-0.47380017956557	-1.08176369186288
H	-0.36516989518200	0.09340658818252	1.24198629234088
H	-0.34738621153246	-2.20546644380436	0.63222085200704
H	0.04754192070786	-2.48427129587735	-1.07607186329249
H	2.44579305288486	-2.92603967477072	-0.46157521894822
H	2.04510523295987	-2.65921386518912	1.25512219333271
H	3.02095375887211	-5.20263303997814	0.16360261752454
H	2.49024908148639	-4.97072651506448	1.84326459185297
H	2.75625705292295	-7.28289397217269	2.52134136347382
H	1.87669169223544	-9.60749424291951	2.50516743261650
H	0.15722739761357	-10.28391325194811	0.82458851371963
H	0.22927121842568	-6.29017564611163	-0.82187320110905
H	-0.66670712688835	-8.61449644066396	-0.83883034601839
H	0.16004054793319	3.86836913498854	3.69364767418520
H	-1.28462443497812	3.15537041984711	4.46535895086456
H	0.04548975344967	2.10053324040895	3.92129176559691
H	-2.75186886754402	1.05179846811010	0.95276574973844
H	-2.12948959853874	0.50185237751562	2.54015273169358
H	-3.47046124901285	1.66788099783589	2.47008723903434

H	-0.36211236929826	5.25445179135501	0.48987110935006
H	-1.60785056713774	6.46311797070940	0.89525899309461
H	-0.60940038462559	5.74877831235624	2.18310045554317
H	-2.15675322751461	3.82735220171366	-0.72535681651959
H	-3.65856564819027	3.40183756374486	0.11456200391836
H	-3.34056501829278	5.09872164653147	-0.32214691630516
H	-3.73986806186869	5.77477101249558	2.09648723842194
H	-4.00960989190435	4.08824061862365	2.58723977947341
H	-2.77629721487180	5.07274704503893	3.41611351180030
C	1.85966232756925	1.06804415602765	-3.97113931388471
H	2.67806937102079	0.70785695163286	-3.32717060545909
C	0.60186591010914	0.20905239750959	-3.76332674926958
H	0.82191971184865	-0.82408462435143	-3.45357378497035
H	-0.01237216340148	0.15201702876381	-4.68040830712248
N	-0.15838982109566	0.92428215841016	-2.73096527937919
C	0.35608099605493	2.13055222107604	-2.47159936034119
H	-0.32009646962513	2.89594852670296	-2.08539995417197
B	-1.59394001809429	0.49290143726667	-2.33881516821140
F	-1.62765036812280	-0.88178231462002	-2.08040489429372
F	-2.46161692603130	0.78179261700209	-3.40398917348795
F	-1.97745675740119	1.23317421855131	-1.20440633568791
H	2.21970868318521	1.04819321384166	-5.00928221733795
C	1.40367697105670	2.46308768648139	-3.51653077183177
H	2.21290000807118	3.11491178326399	-3.16194799362202
H	0.88489929947235	2.99365074306874	-4.33628681580189
H	1.76083774986696	3.23505817881753	-0.75752156395446

FPAN

Total Enthalpy ... -1715.59045967 Eh
Final entropy term (T*S) ... 0.09528265 Eh
Final Gibbs free energy ... -1715.68574232 Eh

C	1.29647587075067	2.19782667090027	-1.12406852873743
C	0.84189614228776	-0.43676419473616	-0.79973018946629
C	0.23937791661685	1.81845714921124	-0.12731300810899
C	0.02372194656441	0.50171095166141	0.04475586225437
C	0.50491030335537	-1.91314626535353	-0.52936151512293
C	1.72858777059287	-2.79857973558095	-0.34867664950614
O	1.28116723888906	-4.07480542252119	0.05854992865766
C	2.32090271715959	-4.99737045605008	0.26160504838746
C	1.77814224360930	-6.32068799354634	0.74574126879193
C	2.66826907780256	-7.31175335028695	1.18888396422837
C	2.19370910583517	-8.54889295415129	1.62974561913429
C	0.81823791463434	-8.81194503268453	1.63671266173588
C	0.40370384932852	-6.58949175695091	0.75464984229834
C	-0.07291767924975	-7.82833877584720	1.19908958160675
O	-0.39677138810634	2.83876500238081	0.47096163096935
Si	-1.71570833423738	2.82033743454266	1.57344844317434
C	-1.19097697809095	1.92394944521930	3.13639681444190
C	-3.17649759653132	1.98495018285921	0.74344219336188
C	-2.01540538313061	4.67376659664184	1.87683837076054
C	-0.75499812476069	5.30198938426702	2.50192786578956

C	-2.32843563173585	5.37253924650658	0.54041525307552
C	-3.20705561295777	4.84366122934487	2.83900744729360
H	2.28714797325145	1.96229057642505	-0.69909603039161
H	1.91493685355820	-0.28472435765474	-0.60419483578851
H	-0.72602155849776	0.09538594510627	0.72274584572701
H	-0.07641301807689	-1.96848712100487	0.40342319802071
H	-0.12967840652052	-2.33558752124591	-1.31921282751009
H	2.31324569618856	-2.87175510416205	-1.28171893706766
H	2.39789455508666	-2.36636990397493	0.42477202540142
H	2.89227531011440	-5.14970653537139	-0.67924308596036
H	3.05085311192625	-4.60595055793343	1.00120947739436
H	3.74399365839957	-7.10947547658257	1.19106399302172
H	2.89911678548492	-9.31039391897675	1.97355130258111
H	0.44508916814244	-9.77887272081235	1.98471956209881
H	-0.28986578564685	-5.81914382670562	0.41519017874201
H	-1.14897167764454	-8.02375242338737	1.20299865913693
H	-0.25064992178021	2.34007677076958	3.53218663340756
H	-1.96507973986930	2.03175645028763	3.91413895700144
H	-1.04143979925845	0.84786876359729	2.95812133719352
H	-3.40838489179159	2.46411455868894	-0.22110956399512
H	-2.98048471535690	0.91786160111148	0.55787389745691
H	-4.06992805483510	2.05791925650967	1.38510789620487
H	0.12575419849570	5.17997351963010	1.85125865389400
H	-0.90656340364787	6.38507533246545	2.66176198939058
H	-0.51569619241089	4.85522457756528	3.48079139445420
H	-1.50063033707487	5.26506327418855	-0.17848618635870
H	-3.23962062864036	4.96893175776112	0.06912246094674
H	-2.49231244013149	6.45333390998704	0.70367302852603
H	-3.39009398918833	5.91604861285470	3.03375991342584
H	-4.13716582891040	4.42236929238996	2.42307161678206
H	-3.02350025229166	4.36052068126695	3.81296888520725
C	-1.20897999054753	1.32993399720774	-3.00101334119029
H	-1.97391968178619	1.36774354474117	-3.79064929989112
C	-0.74283788846439	-0.10020297996431	-2.74594277082364
H	-0.76271860951666	-0.69459169538416	-3.66645974469768
H	-1.33975416947921	-0.62191176355775	-1.98966039594768
N	0.70413445615077	-0.01882103740963	-2.27801202172978
C	1.14543606084067	1.44855932822832	-2.46417856683074
H	2.12587928684585	1.42911370206938	-2.95073785564821
B	1.64025688076032	-0.92603316395137	-3.25808471915932
F	1.10648950836732	-2.20466031556215	-3.32571108348094
F	2.91829414877449	-0.92788598878323	-2.71455799786434
F	1.62234684605568	-0.33187823988887	-4.51415443784759
H	-1.64259581823805	1.76720892379255	-2.09005279289771
C	0.08230013683488	2.04868117947181	-3.39117598433351
H	0.34183268432056	1.81892141603019	-4.43456564328779
H	0.02683803941195	3.14135392485659	-3.28466797212158
H	1.27629607196957	3.27886639948455	-1.31571671621103

FPAX

Total Enthalpy ... -1715.59250467 Eh
Final entropy term (T*S) ... 0.09224928 Eh

Final Gibbs free energy ... -1715.68475395 Eh

C	1.17832402504980	2.31880810138654	-0.95929395856500
C	0.80310427953560	-0.37175424303816	-1.05204022002262
C	0.65139340076671	1.66124054880591	0.28394955363086
C	0.46230701106609	0.33566210579051	0.22693070289384
C	0.45840477103178	-1.85994778839676	-0.98029860781024
C	1.41786838089958	-2.60016688132063	-0.05974785348127
O	0.98312292787958	-3.93763437474170	0.03561949725556
C	1.78252539748930	-4.73866327814629	0.87005437081029
C	1.25854917983200	-6.15424939241683	0.90799033317830
C	1.72743294812246	-7.04191449317950	1.88887587384646
C	1.27717611501745	-8.36357375826026	1.92640225672751
C	0.34463021604521	-8.81545778982680	0.98486459687824
C	0.32589295083451	-6.61218235481944	-0.03163475424109
C	-0.12946102158414	-7.93475487325916	0.00854167010389
O	0.38475719353739	2.45538065810590	1.34155025250430
Si	-1.04776939660447	2.65893436780116	2.26099283564206
C	-0.42899629802233	3.06738823743462	3.98047457266785
C	-2.07801696696880	1.09177081233502	2.24843147228455
C	-1.99778485990265	4.11712045183038	1.47859058547472
C	-1.11665408137670	5.37969189112953	1.50698430793654
C	-2.35760118971428	3.78223287778698	0.01968175277910
C	-3.28897560603105	4.36965397103398	2.28094679085558
H	2.24462992980786	2.07155315395009	-1.10384281330609
H	1.88779626192166	-0.28460303360856	-1.24443587677808
H	0.04074401140430	-0.23311525041331	1.05458450087910
H	-0.56676045139514	-1.99054962260692	-0.60555743567093
H	0.50021085721584	-2.32972868127487	-1.97172642587795
H	2.45149143105512	-2.55449590862188	-0.46227405593634
H	1.44764581831789	-2.13532727065227	0.94611010540932
H	2.83394383359633	-4.74068417681478	0.50926750740890
H	1.81256513954026	-4.32341321154054	1.89890899808009
H	2.45046185915429	-6.69260881131320	2.63265955170296
H	1.65110118487957	-9.04304504418943	2.69724393304234
H	-0.01235498050437	-9.84836135148316	1.01566956354154
H	-0.04520681618071	-5.92316088482988	-0.79157053856370
H	-0.86034175123336	-8.27751053343112	-0.72923015913917
H	0.25904548789931	3.92759602130103	3.96511547442137
H	-1.26856898069380	3.31184615496339	4.65148069522899
H	0.10895219961492	2.20535818791412	4.40804298716419
H	-2.31246865462708	0.76594875817035	1.22431018936539
H	-1.55422313670766	0.27014079391953	2.76307203930218
H	-3.02579877950789	1.27276004819431	2.78189304847316
H	-0.16690736154120	5.22328493388111	0.96985750427424
H	-1.63970655674455	6.22413160043339	1.02171149651949
H	-0.87435263447255	5.69023302471842	2.53658235835706
H	-1.45625125747184	3.66469234777849	-0.60116901323630
H	-2.94558818794499	2.85484085109140	-0.06779981668376
H	-2.95430907775165	4.59932463144747	-0.42506193059735
H	-3.83495920214016	5.23406169833592	1.86068282373301
H	-3.97101274321067	3.50433804784634	2.24814661827817
H	-3.08153436416980	4.59589811417442	3.34007843171307

C	1.83115014098416	1.04543263808825	-3.89509637567061
H	2.75568555982174	0.98903256193799	-3.29761514704652
C	0.88456493977812	-0.09039995384917	-3.53857817990052
H	1.37620933267371	-1.05586486487985	-3.37471628710641
H	0.12732983618477	-0.22240228325338	-4.32039446644157
N	0.17509464934488	0.34235903670937	-2.26648421307540
C	0.36072673037676	1.87699569787508	-2.18150965537691
H	-0.64167943751720	2.30239344873299	-2.08462054372860
B	-1.42470209784594	0.04484044582105	-2.43844162509480
F	-1.58210711192842	-1.27505511391337	-2.83057559377768
F	-1.87898653188001	0.90605445077805	-3.43300440982154
F	-2.05615633375197	0.30393918606586	-1.23086746713469
H	2.11528057264898	1.01999644873604	-4.95715816875400
C	1.00756157018839	2.27410668211530	-3.51283216422420
H	1.60414280195445	3.19203949958357	-3.40816510788717
H	0.22829293594814	2.45486914631792	-4.26830423401004
H	1.10440998800646	3.41224358975942	-0.87916215340365

DB

Total Enthalpy ... -1219.56099825 Eh
Final entropy term (T*S) ... 0.08614976 Eh
Final Gibbs free energy ... -1219.64714801 Eh

C	23.57022386472656	-11.89608190572276	-2.06807758255074
C	22.73998906973332	-14.72857072469801	-2.95851720775306
C	22.31111568881248	-12.33860798084721	-2.27395283354498
C	21.93992848314715	-13.74742902242186	-2.50806748584264
C	23.97098881726363	-10.46504491232471	-1.88619987068745
C	22.31361995669193	-16.15675751898220	-3.12702578433992
C	23.03016615211174	-17.08846672227702	-2.15218740067190
O	22.62958915617689	-18.41561482043503	-2.41439888889628
C	23.23157281703860	-19.36497329148877	-1.57047349583150
C	22.79889000060225	-20.76380551529081	-1.94027726634414
C	23.14719557499519	-21.83757672196313	-1.10542904755676
C	22.77680833367699	-23.14337007089441	-1.43380810924995
C	22.04663132449765	-23.39411368635783	-2.60218654790449
C	22.06960814078988	-21.02068841950738	-3.10791383790590
C	21.69445092773448	-22.32893602715459	-3.43547873099728
O	21.24990629891705	-11.46805087113363	-2.30773814279662
Si	20.23701791382876	-11.01776016770619	-1.01364980188715
C	21.29792057239878	-10.51793545541516	0.45386309552556
C	19.15318717502344	-12.47829939888928	-0.53490203651100
C	19.21850051583931	-9.57479381984292	-1.72554309753631
C	20.16477042730461	-8.46115634803887	-2.21234070551390
C	18.37274131557204	-10.08068103876194	-2.91000732590309
C	18.29158609623952	-9.01954465497112	-0.62629278948031
H	24.36485029309468	-12.64770213513394	-2.04448731745381
H	23.78437829732164	-14.50082893258114	-3.20735116604789
H	20.88808561092467	-13.98271464452540	-2.30807610135801
H	24.78413601560904	-10.19220743299602	-2.58238319312198
H	23.12788381006209	-9.78170188755982	-2.05740180077264
H	24.35953246240769	-10.27674238255486	-0.86852543106472

H	21.22536635758376	-16.25652466329940	-2.98149862840321
H	22.54375944328924	-16.50483574451466	-4.15073866322463
H	24.13047100808577	-16.98952232272844	-2.26094047532794
H	22.78360000310359	-16.80810790384266	-1.10754518516411
H	24.33871638149156	-19.29162324025085	-1.63580359097699
H	22.97396000980794	-19.16392373597917	-0.50932721794232
H	23.71316485312685	-21.64731544185107	-0.18797770113733
H	23.05510782878240	-23.96936709259290	-0.77349234649884
H	21.75360296030241	-24.41570034650700	-2.85876843014142
H	21.79534320377231	-20.18789491482527	-3.75676031968208
H	21.12288952587813	-22.51467593990964	-4.34923707290986
H	21.87161553371401	-9.59952053771610	0.25627520612404
H	20.66791931130777	-10.34894290653686	1.34287761554504
H	22.01230701754292	-11.32276369578028	0.69372091774547
H	18.59741730799515	-12.87182771843564	-1.40090475712734
H	19.76462549527579	-13.29555238241302	-0.11808913738936
H	18.42235591554673	-12.18012257506195	0.23530153207464
H	20.84062972949952	-8.82142483031537	-3.00398513154417
H	19.58153318173941	-7.61748044712279	-2.62486868407310
H	20.78686106793778	-8.06224246062330	-1.39418155701530
H	19.00451296822991	-10.51255336619161	-3.70317736312374
H	17.64942326139605	-10.85165235184270	-2.59759499240697
H	17.79727128226629	-9.24808096239163	-3.35470320927867
H	17.68549166944638	-8.18394222246144	-1.02176324445284
H	17.59117937340625	-9.78409014464788	-0.25086998227948
H	18.86184019692960	-8.63387554168212	0.23515232260940

TSBN

Total Enthalpy -1754.78846491Eh
Final entropy term (T*S) 0.10098280 Eh
Final Gibbs free energy -1754.88944771 Eh
Imaginary mode -335.22 cm**-1

C	1.56254960690488	2.28940605689818	-0.84344577103057
C	1.02345542410137	-0.59756606579462	-0.37972244534087
C	0.48775924435509	1.80114706613476	-0.08820770175154
C	0.23052829477360	0.39475025617645	0.09108333040327
C	1.90354098713599	3.75694515202603	-0.81420463849744
C	0.64883857366798	-2.03951445320874	-0.32570932206091
C	1.81212709088978	-2.92927832248762	0.09484748089870
O	1.34326411933192	-4.25560890509237	0.18706746500424
C	2.33054069749011	-5.18020084680230	0.58526063718077
C	1.69815260219931	-6.50413152035109	0.93910570631595
C	2.39268702657962	-7.70199444542616	0.71938847568745
C	1.83780141427489	-8.92786972872293	1.10039288265556
C	0.57324668409403	-8.97049799212064	1.69671921024053
C	0.42934109293027	-6.55486785424748	1.53550307116852
C	-0.13032365589133	-7.77979903644631	1.90960258190472
O	-0.40831687105136	2.68114624378534	0.34278919288535
Si	-1.68334063142809	2.66633110105816	1.51036255209055
C	-0.96811252953885	1.98227402930978	3.10022871280637

C	-3.11609180204782	1.63559936525183	0.87843284363792
C	-2.12560561459437	4.50990487659689	1.61664901866004
C	-0.87775768421511	5.32184808048162	2.01228692463316
C	-2.64006697379137	4.99127608630635	0.24634612271744
C	-3.22491412840186	4.70070095693526	2.67990912045496
H	2.40984275675368	1.61014349081563	-0.93904948801895
H	1.99683196715676	-0.36441110251751	-0.81433678899048
H	-0.71750829596709	0.12189217981849	0.56143185571002
H	2.64347339920134	4.00034180348514	-1.59212434914624
H	1.01624102096547	4.38755680017602	-0.96981793102531
H	2.33820587022708	4.03888256550175	0.15983750559525
H	-0.20842836642015	-2.19859701438038	0.34843805835959
H	0.33735312904346	-2.35131841132716	-1.33941455086614
H	2.63046386648343	-2.84936879513453	-0.64845692896700
H	2.22260907853522	-2.59861318361809	1.07130258979515
H	3.08757500213174	-5.31857097195556	-0.21260887903720
H	2.87677489569617	-4.78707908833566	1.46968720939851
H	3.37759732686753	-7.67503988390160	0.24355147959794
H	2.39177463943704	-9.85367360881498	0.92272561979733
H	0.13552938057611	-9.92837220224103	1.99045420070525
H	-0.11948050515370	-5.62506791834425	1.69784041301234
H	-1.12131528676045	-7.80502980887914	2.37149181623925
H	-0.08183175930988	2.55659659976879	3.41330824191848
H	-1.71622025965701	2.03779303357423	3.90806230957906
H	-0.67337007691308	0.92722073963918	2.98598202814652
H	-3.40982509272488	1.93684364019708	-0.13924469741163
H	-2.88082554477470	0.56024850884474	0.86648803219374
H	-3.98718588438245	1.77962725978221	1.53908312795877
H	-0.06824501299625	5.20819447911037	1.27479556059367
H	-1.12791691113591	6.39629988947922	2.07472632931133
H	-0.48429446974243	5.01783869160135	2.99607455614760
H	-1.88541954143367	4.85214299484359	-0.54460791092110
H	-3.55403227280730	4.45729474330488	-0.06062311432044
H	-2.88509609990271	6.06793550528940	0.28838792180485
H	-3.50124629104910	5.76807969299284	2.75285581376457
H	-4.14239820730806	4.14118285047415	2.43340475561264
H	-2.89176302487986	4.37853046560332	3.68036140414710
C	-1.33468211015944	1.40956422397730	-2.83727433882494
H	-2.23761249374494	1.53767023542513	-3.45105213150546
C	-0.49491337523239	0.20018957226574	-3.27328638316098
H	-0.60452613220651	0.00248085765428	-4.35601606258807
H	-0.75835487015763	-0.72722278417739	-2.74417306133638
N	0.89079537417072	0.60190925022773	-3.00321076733129
C	0.98871669289830	1.91728154620424	-2.72820347815821
H	1.90208228566302	2.41985698232559	-3.05979997373951
B	2.09500430175392	-0.26636330067671	-3.43583751251576
F	1.78782034842762	-1.61819767476205	-3.22359454660676
F	3.21349546519633	0.10945889454189	-2.66142751534435
F	2.37507931865230	-0.05707258083484	-4.79329330231927
H	-1.65987015958820	1.29471763064580	-1.79357803625813
C	-0.35264500830941	2.58510212171260	-2.96087453614327
H	-0.35078537255107	2.99450850226732	-3.98696516803468
H	-0.56683666233769	3.41655247809048	-2.27616482748158

TSBX
Total Enthalpy ... -1754.78453258 Eh
Final entropy term (T*S) ... 0.09920354 Eh
Final Gibbs free energy ... -1754.88373612 Eh
Imaginary mode -328.22 cm**-1

C	1.48565637024134	2.23238163026768	-0.79629248928191
C	0.86169340131303	-0.65548858943349	-0.40975177630060
C	0.60928153741702	1.73150317817025	0.17939606167857
C	0.33636112588446	0.32689978516047	0.35451592696075
C	0.47913636928251	-2.09203104940742	-0.29216039186288
C	1.64281720454409	-2.96988793767902	0.16300370564177
O	1.17565540646805	-4.29464635899189	0.27345337257522
C	2.14452389741465	-5.20184607551933	0.74778592964882
C	1.56099471117693	-6.59133808823307	0.82566444960501
C	2.02417813219466	-7.49948285795226	1.78860303585907
C	1.51512255600482	-8.80059875306408	1.84434602635401
C	0.52700125176491	-9.20683907071028	0.94128512579124
C	0.57044337724463	-7.00556035913031	-0.07664798893216
C	0.05506089391139	-8.30360289620642	-0.01724929723982
O	-0.06659073966259	2.61401496494747	0.88819932296934
Si	-1.37361794066260	2.74678244371086	2.00936406147934
C	-0.54036228417663	2.83736216635170	3.68478649169478
C	-2.56631729464036	1.30521325736871	1.92915480611594
C	-2.17918420515865	4.38988350365337	1.48747381724416
C	-1.15810852979452	5.53513290660710	1.62499800936358
C	-2.65152836775108	4.30163909849525	0.02425140474078
C	-3.38681258219697	4.66676409637752	2.40449338688851
H	2.27087848234596	1.54314437975781	-1.11115517282106
H	1.64626510138171	-0.42322205093544	-1.13425085064626
H	-0.42038200790274	0.06223796438024	1.09427173063135
H	-0.36044661065480	-2.21427196892505	0.40955250667568
H	0.13535526928716	-2.44913752012138	-1.27845114476439
H	2.48417601125360	-2.90929638202465	-0.55857981014119
H	2.02986249114841	-2.61408051185015	1.13964217891394
H	3.02816780179126	-5.20174105635167	0.07289356699753
H	2.51572617879988	-4.89216005690174	1.74555393549014
H	2.78735024699422	-7.18336613338210	2.50628780884492
H	1.88501874974693	-9.49636148895964	2.60247349777609
H	0.12244359671079	-10.22148191398135	0.98767480434246
H	0.19792035293755	-6.29847544308983	-0.82020022258767
H	-0.72078208119594	-8.61148809006798	-0.72372654425634
H	0.21577693374220	3.63715002246349	3.71689632832081
H	-1.28183766558399	3.02995979331045	4.47759635715626
H	-0.04200540752467	1.88022503912946	3.91120441281173
H	-2.82060362790085	1.05606738427907	0.89003105437699
H	-2.15379563975072	0.40994439587784	2.42069390747865
H	-3.48960763736601	1.58102483406828	2.46502275094135
H	-0.26538780724534	5.36000808850299	1.00436242462934
H	-1.61155581964897	6.48848454205606	1.29800681859128
H	-0.82532497217963	5.66917302746170	2.66721095292008
H	-1.80691320299482	4.14823848147827	-0.66441713018105

H	-3.35902104014405	3.47368239319246	-0.13762877996446
H	-3.15786176120467	5.24029205246118	-0.26613165849304
H	-3.84690709693921	5.63645258219163	2.14154856660849
H	-4.16799651617072	3.89586981465693	2.30366571951419
H	-3.09791726295723	4.71887508999810	3.46770642262143
C	1.79116060882770	0.81558162824722	-3.97338607323016
H	2.59405135900780	0.44407737603914	-3.31571968209811
C	0.48917513182709	0.04828541633848	-3.71223750382358
H	0.64836487727042	-0.98917185680650	-3.38033334072132
H	-0.14693847780724	0.00808989972168	-4.61574042683923
N	-0.19610680240416	0.84311784493477	-2.68607725066110
C	0.37760852984313	2.04674101013674	-2.50426264010949
H	-0.27769292996686	2.86923311444471	-2.20611569160126
B	-1.66244270857977	0.53371373256864	-2.30499427027700
F	-1.80427832999187	-0.82468049562794	-2.00225513618221
F	-2.49817673910379	0.85042861910463	-3.39023460832926
F	-2.01404194795042	1.33574608894509	-1.20147265169380
H	2.14090329392311	0.71919352098870	-5.01097184271173
C	1.42025604564563	2.25507660474043	-3.59164218953981
H	2.27621877820263	2.87506811005528	-3.29973194772773
H	0.91499075132514	2.76660076882822	-4.43125884950780
C	1.84981606347249	3.69633262317537	-0.74909400755944
H	2.56676545516842	3.95785938782886	-1.53951463130672
H	2.30576701233195	3.96250357671967	0.21942381004178
H	0.95872067936421	4.33305076615911	-0.86908848890281

FPBN

Total Enthalpy ... -1754.82482575 Eh
Final entropy term (T*S) ... 0.09629476 Eh
Final Gibbs free energy ... -1754.92112051 Eh

C	1.40063995525967	2.30002171018440	-1.33019703323551
C	1.14585133869976	-0.37576090236476	-1.32355043994658
C	0.44757322548266	1.71946779816354	-0.31267635012590
C	0.32338292977206	0.37967059906879	-0.32068088516637
C	1.50237465261564	3.82347741180236	-1.32550729195556
C	0.92870445384329	-1.88543911467163	-1.21377779969005
C	1.61289610428957	-2.42954067931545	0.03273143628996
O	1.29348517631075	-3.79810249752165	0.15018973148523
C	1.89936303052941	-4.42479992788209	1.25819115050049
C	1.36411221181435	-5.82581004942229	1.42088748445645
C	2.19148099735122	-6.85004676918610	1.90214673279586
C	1.68731746927112	-8.13939243852259	2.10015748885544
C	0.34902130409574	-8.42169127106426	1.80787745067342
C	0.02333040579539	-6.11663225548914	1.12811174735960
C	-0.48029928988560	-7.40639138719034	1.31796737709835
O	-0.22184534996601	2.59445428648478	0.45128744887577
Si	-1.47169505763549	2.35168762840666	1.60240049481641
C	-0.80124724551585	1.32010875721883	3.01846618976137
C	-2.92194292684424	1.51366053054359	0.75612139077076
C	-1.87722449786470	4.13261124450298	2.13025277771448
C	-0.61891550546429	4.79096135650071	2.72725823782740

C	-2.34228337313749	4.93748466167233	0.90165195233343
C	-2.99777154860836	4.10802376504206	3.18713437058922
H	-0.36735792048688	-0.16477933709930	0.32369269280772
H	2.17765195341812	4.16435447505939	-2.12583678356497
H	0.52514119795536	4.30643739480258	-1.46729080555121
H	1.90574930888110	4.17400267362904	-0.36436491334475
H	-0.14600283116906	-2.11828428871569	-1.14383918730694
H	1.32679804088538	-2.40817669420105	-2.08960437604193
H	2.71096500000778	-2.29128591351539	-0.04521547442770
H	1.28645037127580	-1.88260232172928	0.94065040262938
H	3.00163818753196	-4.44919056997341	1.14268006653137
H	1.69476909168759	-3.83854033335657	2.18033346599510
H	3.24216729748449	-6.63674320174716	2.12069711741298
H	2.34499626786952	-8.92816995311580	2.47534007465636
H	-0.04538778425298	-9.43040206777171	1.95692807422965
H	-0.62070637154143	-5.32436525613873	0.74110759212025
H	-1.52658331881174	-7.62037729865962	1.08260558313416
H	0.11071656890390	1.77240477532071	3.44007059621557
H	-1.54978290866073	1.24658174808152	3.82476020725091
H	-0.55680160244753	0.29714888148754	2.69232953124122
H	-3.24556547583627	2.08260334916788	-0.12986071816285
H	-2.67019611355677	0.49120378733882	0.43573755121776
H	-3.77603500034181	1.44845506150537	1.45051310485241
H	0.20939435376180	4.81703119919681	2.00132643753503
H	-0.83755038177637	5.83289297969744	3.02425220997705
H	-0.26435341728068	4.26007933876761	3.62589163473935
H	-1.57027395816318	4.96285552454386	0.11605039957419
H	-3.26058203144616	4.51861216004770	0.45817815163894
H	-2.56189603336882	5.98179496405787	1.18952489363029
H	-3.24830342872812	5.13731016611141	3.50216905514678
H	-3.92232084204484	3.64970073224564	2.79858082671807
H	-2.69976133804728	3.55192222063436	4.09149965938754
C	-1.28938075509215	1.16174742006039	-3.08844801123777
H	-2.13195873404335	1.19597931491790	-3.79523689460469
C	-0.47118497789189	-0.10896247303837	-3.26190601299888
H	-0.37481329585224	-0.36167894157896	-4.32495196564173
H	-0.88033131172065	-0.98061724369089	-2.74115399459846
N	0.91902100713442	0.19743404347986	-2.73935073978868
C	1.05657574023499	1.74095023555981	-2.73249498116853
B	2.02703409303596	-0.36584595286899	-3.79889887589306
F	1.77678725552749	-1.70779515221230	-4.04959066133122
F	3.27553255277295	-0.17120559255351	-3.22592545005299
F	1.88100170716687	0.37224445700913	-4.96823300943276
H	-1.69595997308611	1.23230420494225	-2.07025482590848
C	-0.25592587797102	2.25736701297843	-3.34832487856419
H	-0.11430707807555	2.38375082675071	-4.43246632502134
H	-0.54964679204036	3.23246619118768	-2.93795557694049
H	2.40515467151689	1.90510754027149	-1.09778229375753
H	1.89971479814274	1.98470722011513	-3.38519095920691
H	2.21751162833019	-0.18316976396236	-1.16587527617769

FPBX

Total Enthalpy ... -1754.82476639 Eh
Final entropy term (T*S) ... 0.09543762 Eh
Final Gibbs free energy ... -1754.92020401 Eh

C	22.92391588369054	-11.58271027134626	-3.78525079218199
C	22.43717829467398	-14.26470497973691	-3.91272118778596
C	22.18745085162053	-12.21488423701412	-2.62128430346112
C	21.97948421368920	-13.53823673122213	-2.68699919637273
C	23.03960344425881	-10.06204253147320	-3.68444566356039
C	22.03354293528850	-15.73914292568733	-3.87858689818406
C	22.84342859136778	-16.51374138568883	-2.84976004770256
O	22.40632510451724	-17.85385286204852	-2.88325547265126
C	23.04368995640874	-18.68982682396514	-1.95158215415365
C	22.52284935706438	-20.10358967948310	-2.06026244117002
C	23.00801882890500	-21.08661961523311	-1.18311556881669
C	22.54753984697185	-22.40228744382935	-1.26467367434260
C	21.59192873712539	-22.75450194482980	-2.22608709856283
C	21.56726547826955	-20.46122056142154	-3.01943575031811
C	21.10489338090321	-21.78004861305298	-3.10116887994584
O	21.77152638362218	-11.39754084147368	-1.63502496869978
Si	20.32777693524816	-11.31801863997657	-0.71008505873762
C	20.91465149344706	-11.00389359068716	1.04193307076173
C	19.33603633359640	-12.90738780349879	-0.82646240561204
C	19.33021364639716	-9.84824129378746	-1.41212652482775
C	20.15904371703983	-8.55438977191307	-1.30537729200459
C	18.98472233686149	-10.12184513299302	-2.88866534561229
C	18.02732411677047	-9.68537155588306	-0.60353352065709
H	23.94453075344628	-12.00613377665514	-3.80567383644544
H	23.53808027956619	-14.21601157957286	-3.99476606317948
H	21.42807664170088	-14.08389409717644	-1.92430249047422
H	23.69704495207072	-9.66072623644220	-4.46814097615043
H	22.05657027370346	-9.57715690025311	-3.78037281044640
H	23.45969849137680	-9.77636489207041	-2.71026305178747
H	20.96577248326739	-15.83280689803077	-3.63573217069014
H	22.18303191585354	-16.21697015605615	-4.85567485811542
H	23.92732946747433	-16.45320519422852	-3.08295514150248
H	22.71153157254826	-16.09208151312742	-1.83315267631176
H	24.14293465298223	-18.68588006140022	-2.11326505322261
H	22.88533006912558	-18.31345943295495	-0.91857947013070
H	23.75422097932497	-20.81716907721183	-0.42900397102049
H	22.93456252714605	-23.15713788217056	-0.57466416592845
H	21.22988892727891	-23.78410790291082	-2.29056546672233
H	21.18797116437278	-19.69891932930911	-3.70088368276118
H	20.35819001096414	-22.04508027961748	-3.85500149897690
H	21.55394712736634	-10.10840587786949	1.09822220218164
H	20.05804392708486	-10.85766013964968	1.72031440369398
H	21.49675388333433	-11.86535139602628	1.40899417506567
H	19.14366015140592	-13.19041042182877	-1.87190713281641
H	19.85276713805223	-13.74254939724415	-0.32711350940885
H	18.36675152106309	-12.76252065451187	-0.32117928242398
H	21.11532731415301	-8.63563022399315	-1.84458103295742
H	19.60045938517788	-7.70503542959958	-1.73977084280459
H	20.38490751057681	-8.29770888632609	-0.25723301292510

H	19.88884513230614	-10.23459239242384	-3.50645884074586
H	18.38595075059132	-11.03843758370406	-3.01170643355841
H	18.40004454968179	-9.28277175594177	-3.30787476237768
H	17.45189695358567	-8.81974411529084	-0.97968781624392
H	17.37549001693009	-10.57051034819038	-0.68445951763163
H	18.22404392106207	-9.50762417418811	0.46697580431140
C	23.77943700156617	-12.92038339555968	-6.66643770303239
H	24.64548075676301	-13.00173642787306	-5.98938919441275
C	22.76252048305860	-14.01343832413112	-6.38201744079583
H	23.20051130208315	-14.99315086822636	-6.16166717325952
H	22.07479733760132	-14.12972437391795	-7.22763806296244
N	21.95892488458486	-13.53282152276190	-5.18500154319917
C	22.20369216200245	-12.00514053580516	-5.08929709474679
H	21.21741006118032	-11.52968368643521	-5.07940771788294
B	20.37200666913358	-13.76250220288497	-5.50814102821151
F	20.19208306921811	-15.08359988792824	-5.88837788624424
F	20.06324504710379	-12.90901214703512	-6.56385634599663
F	19.63164460045154	-13.44641054823813	-4.37767422476861
H	24.15357355792394	-12.97066984877135	-7.69941854004505
C	22.97166713460705	-11.65966192366118	-6.37220424987302
H	23.58860610583324	-10.75968797078843	-6.25348398006905
H	22.25662551457633	-11.47451909376025	-7.18761365739362

NMR spectra of the synthesized compounds

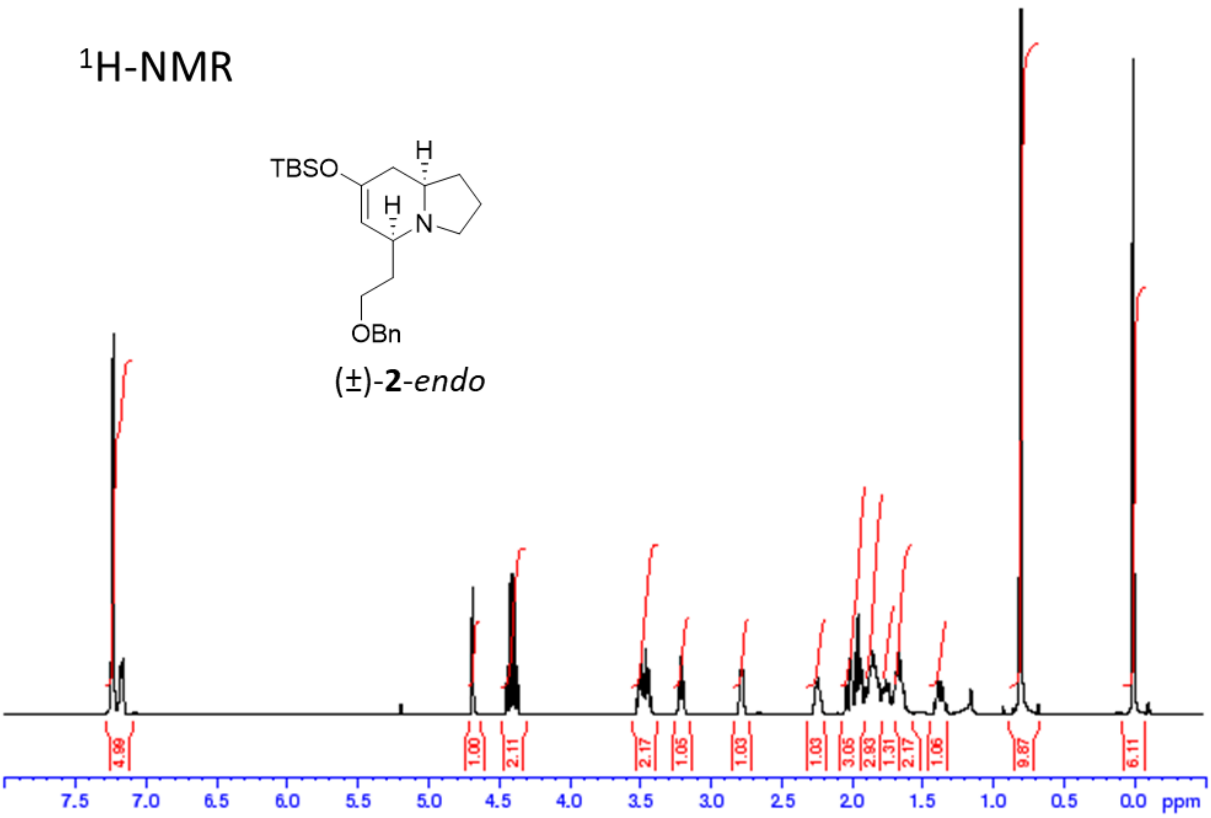

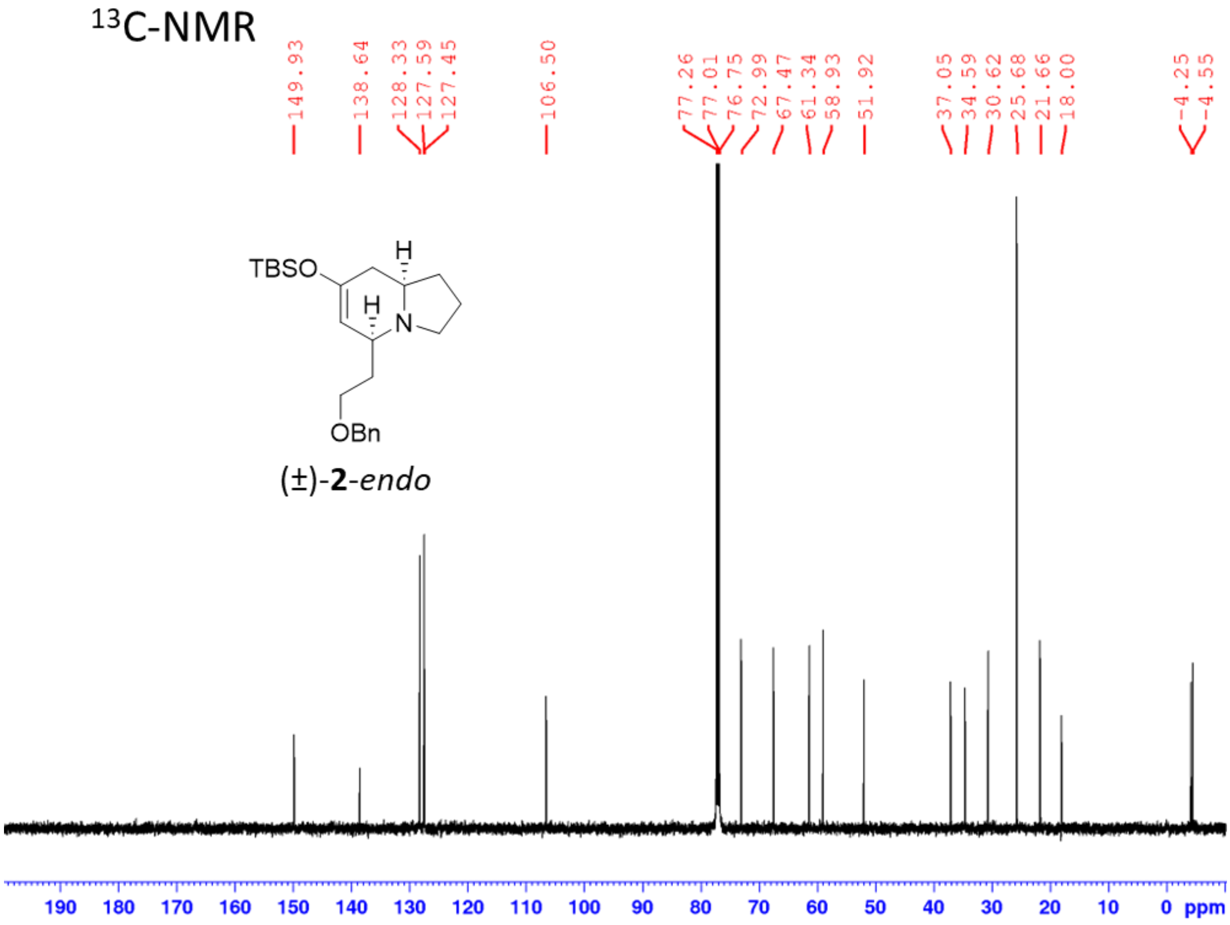

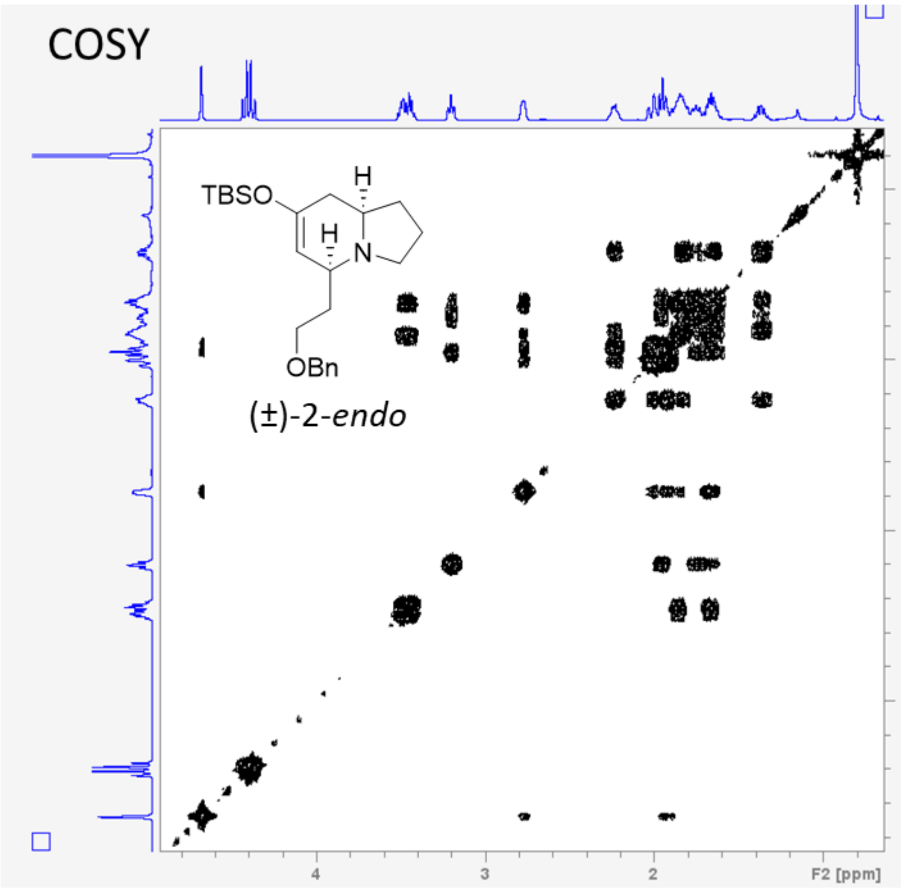
COSY
TBSO
H
H
N
OBn
(±)-2-*endo*
4
3
2
F2 [ppm]

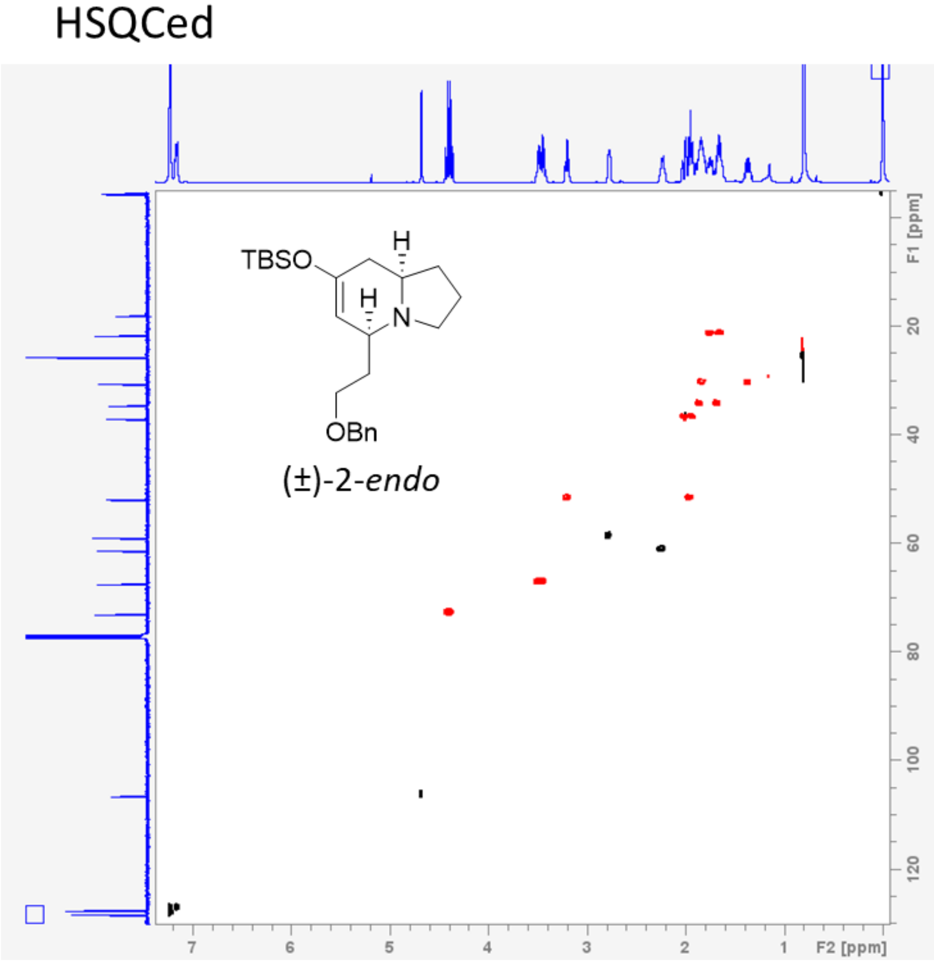
HSQCed
TBSO
H
H
N
OBn
(±)-2-*endo*
7
6
5
4
3
2
1
F2 [ppm]
F1 [ppm]
20
40
60
80
100
120

ROESY

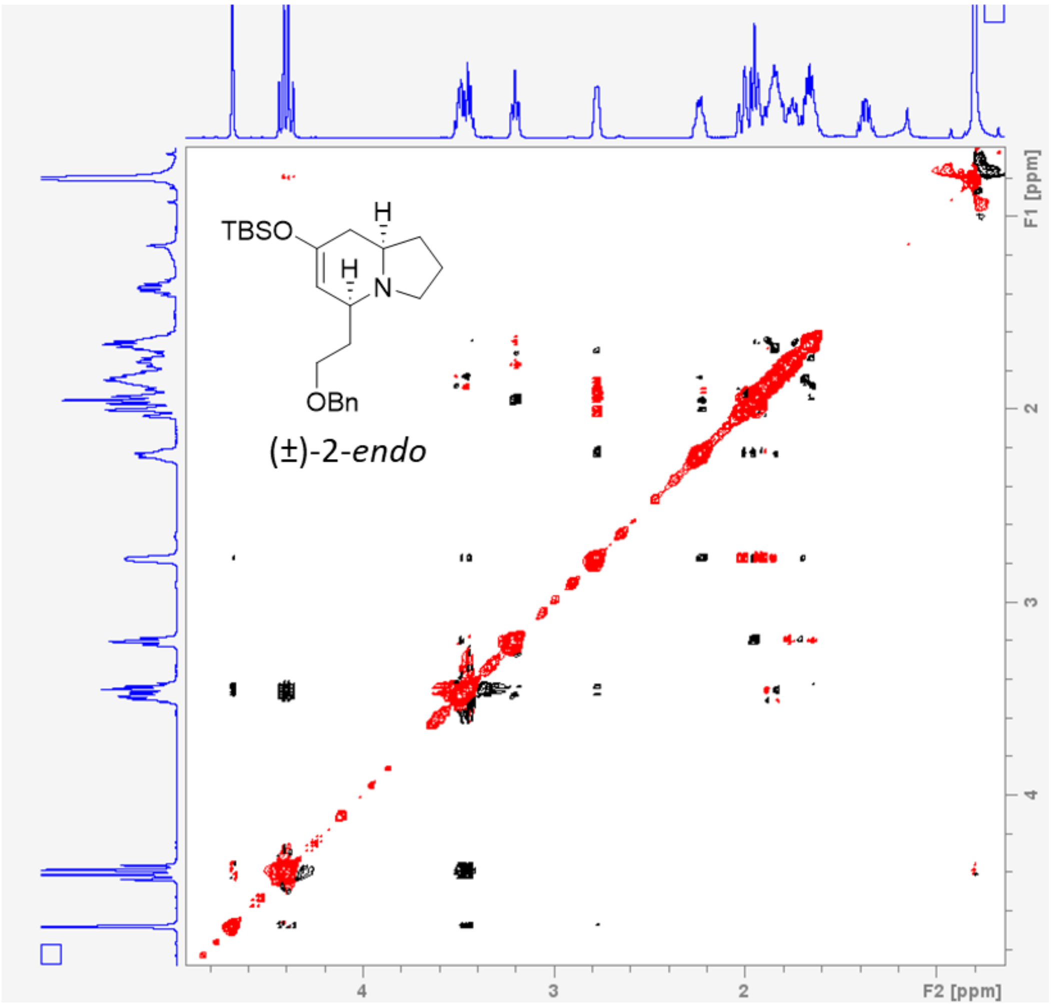

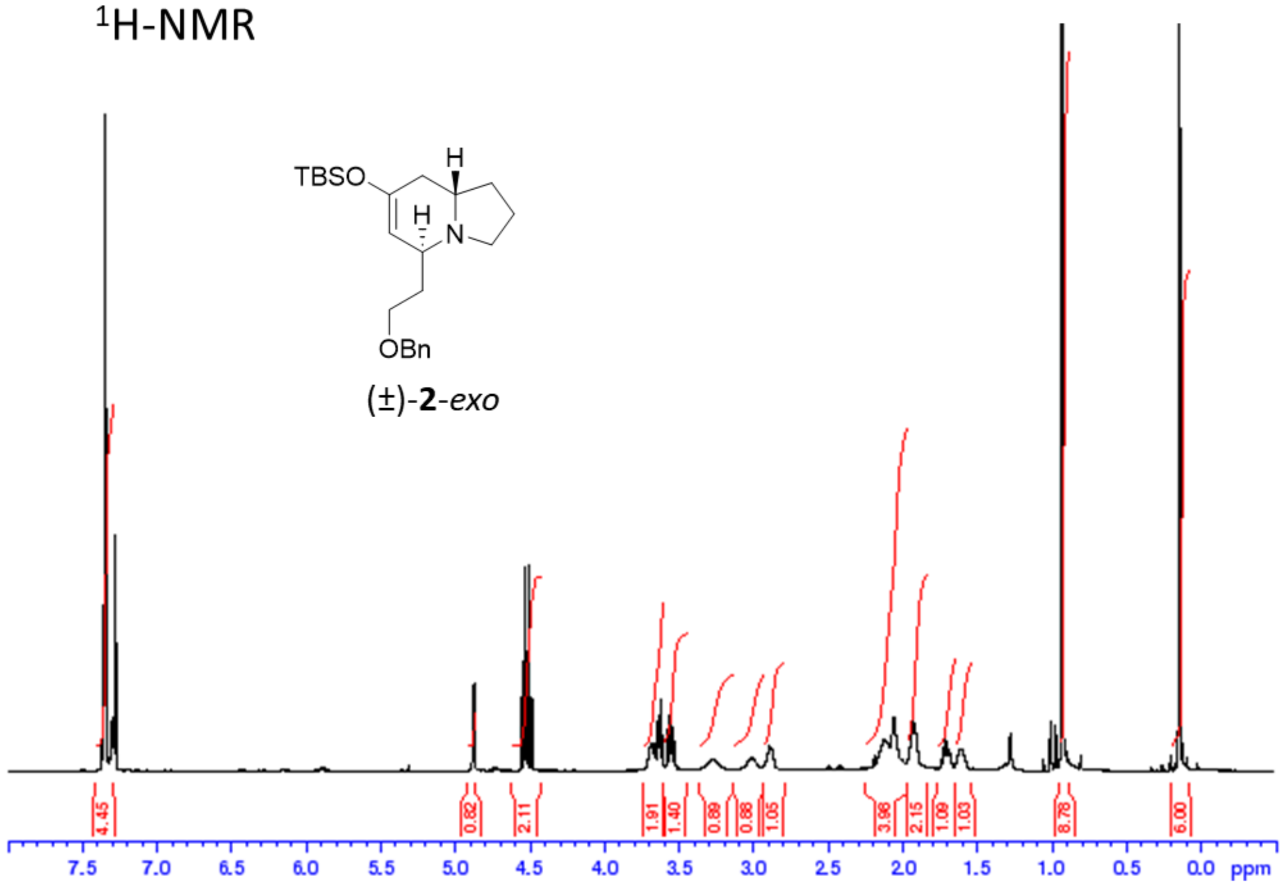

1H-NMR
TBSO
H
H
N
OBn
(±)-2-exo
4.45
0.82
2.11
1.91
1.40
0.89
0.88
1.05
3.98
2.15
1.09
1.03
8.78
6.00
7.5
7.0
6.5
6.0
5.5
5.0
4.5
4.0
3.5
3.0
2.5
2.0
1.5
1.0
0.5
0.0
ppm

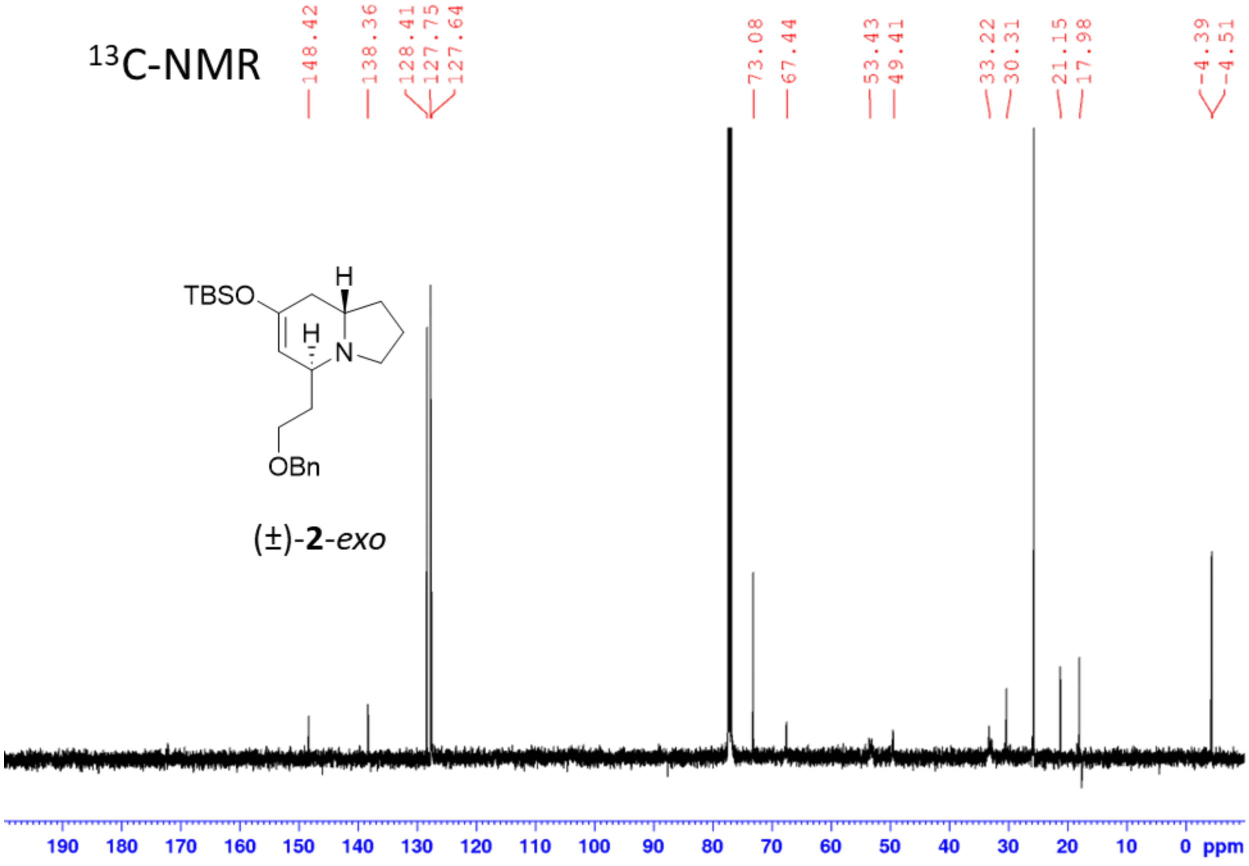

13C-NMR
148.42
138.36
128.41
127.75
127.64
73.08
67.44
53.43
49.41
33.22
30.31
21.15
17.98
-4.39
-4.51
TBSO
H
H
N
OBn
(±)-2-exo
190
180
170
160
150
140
130
120
110
100
90
80
70
60
50
40
30
20
10
0
ppm

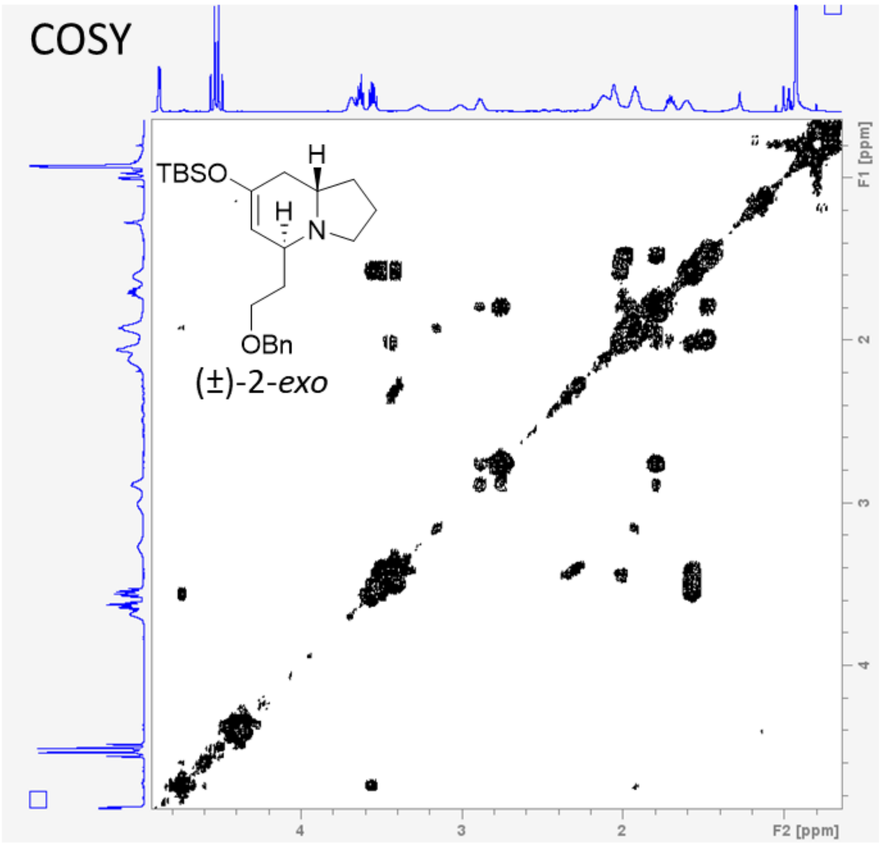
COSY
TBSO
H
H
N
OBn
(±)-2-*exo*
F1 [ppm]
F2 [ppm]

HSQCed

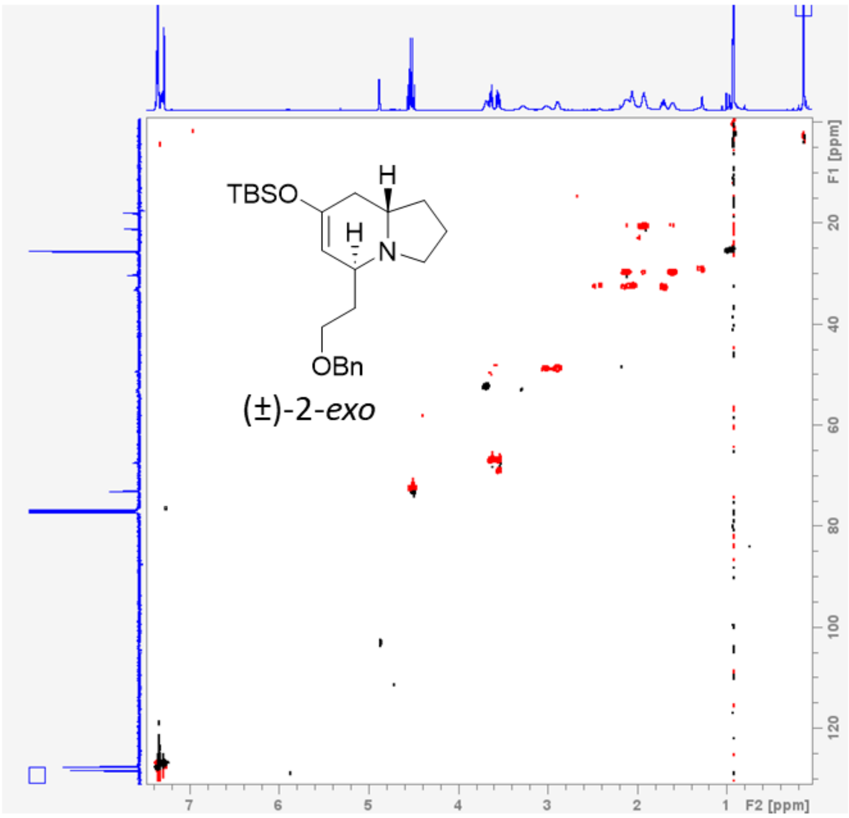
TBSO
H
H
N
OBn
(±)-2-*exo*
F1 [ppm]
F2 [ppm]

ROESY

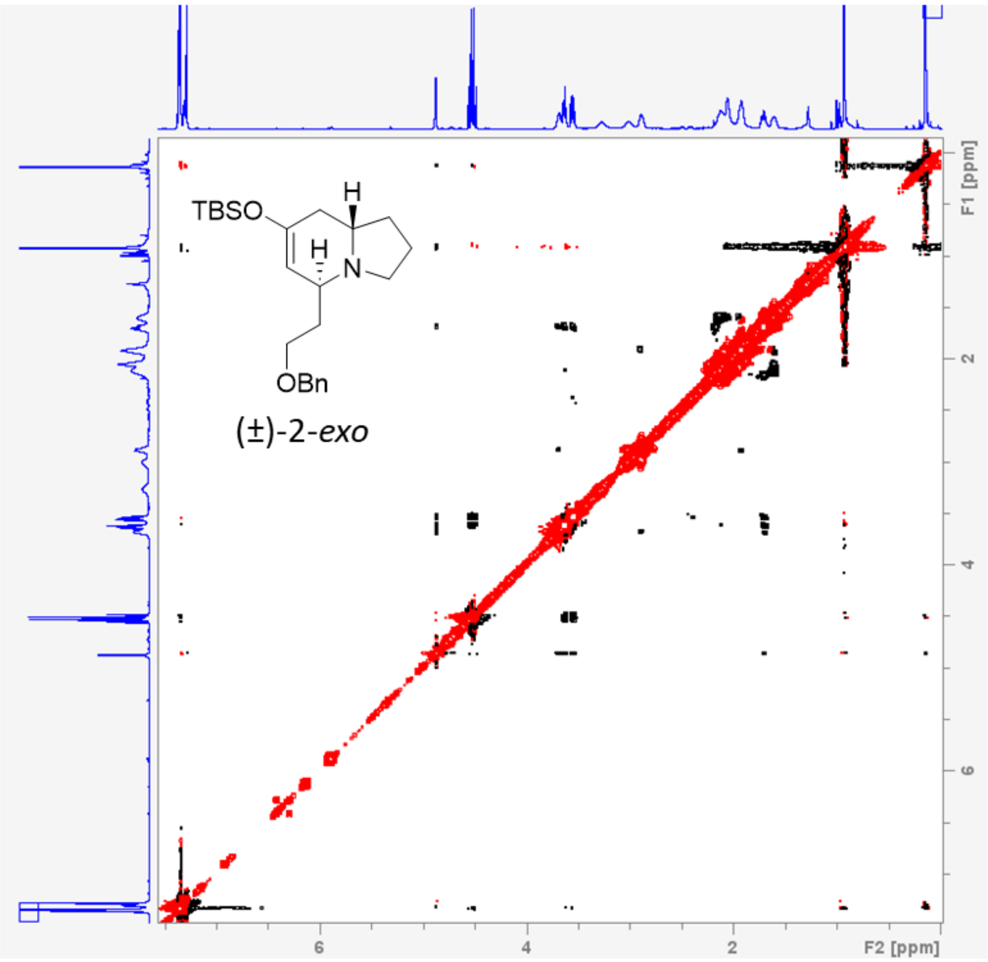

^{1}H-NMR

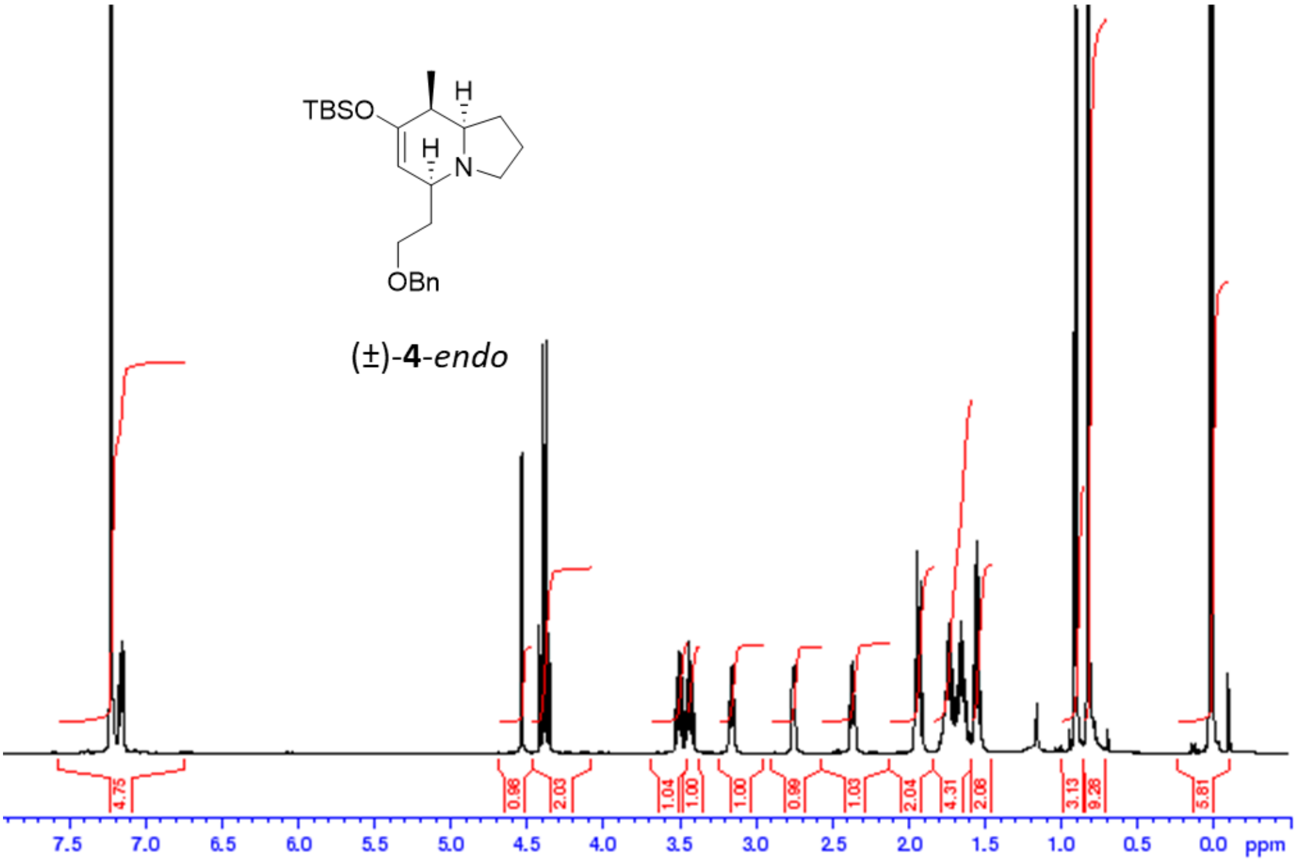

^{13}C-NMR

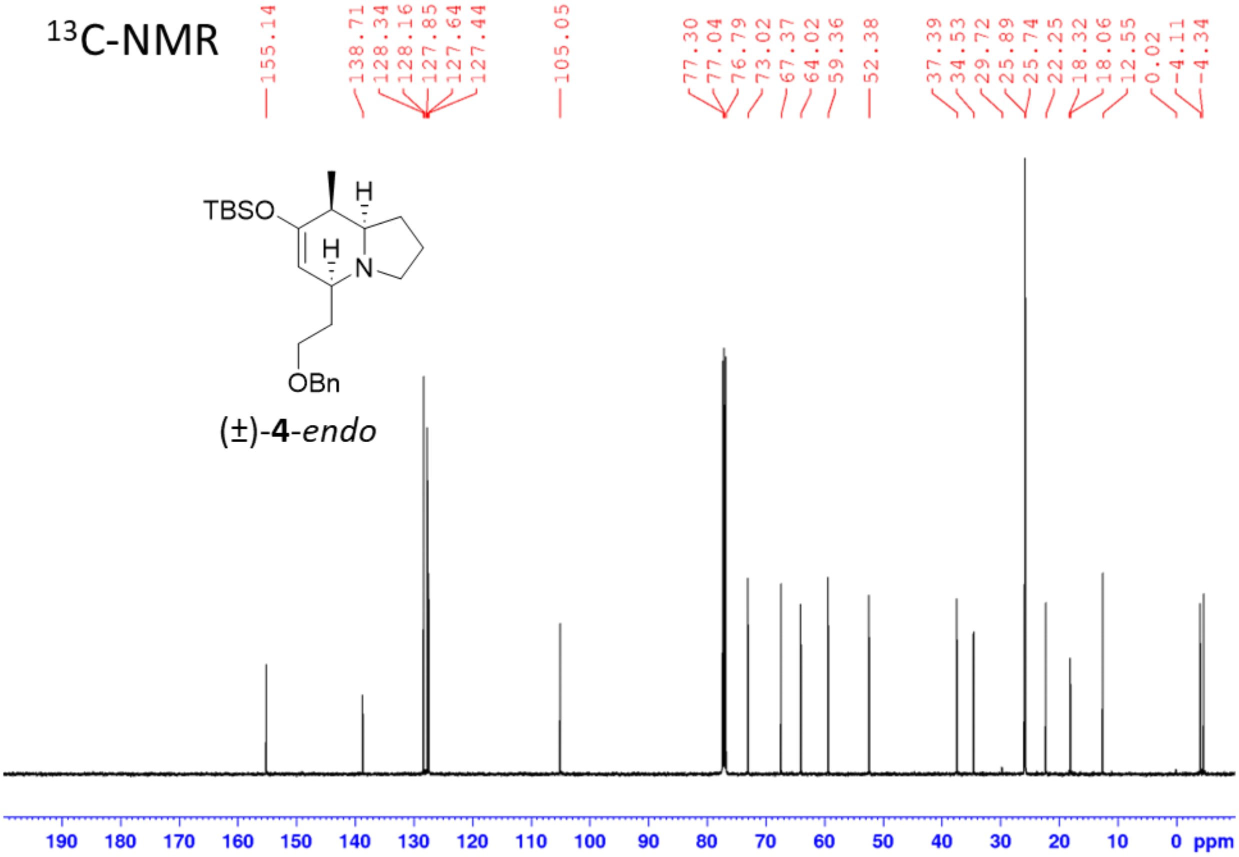

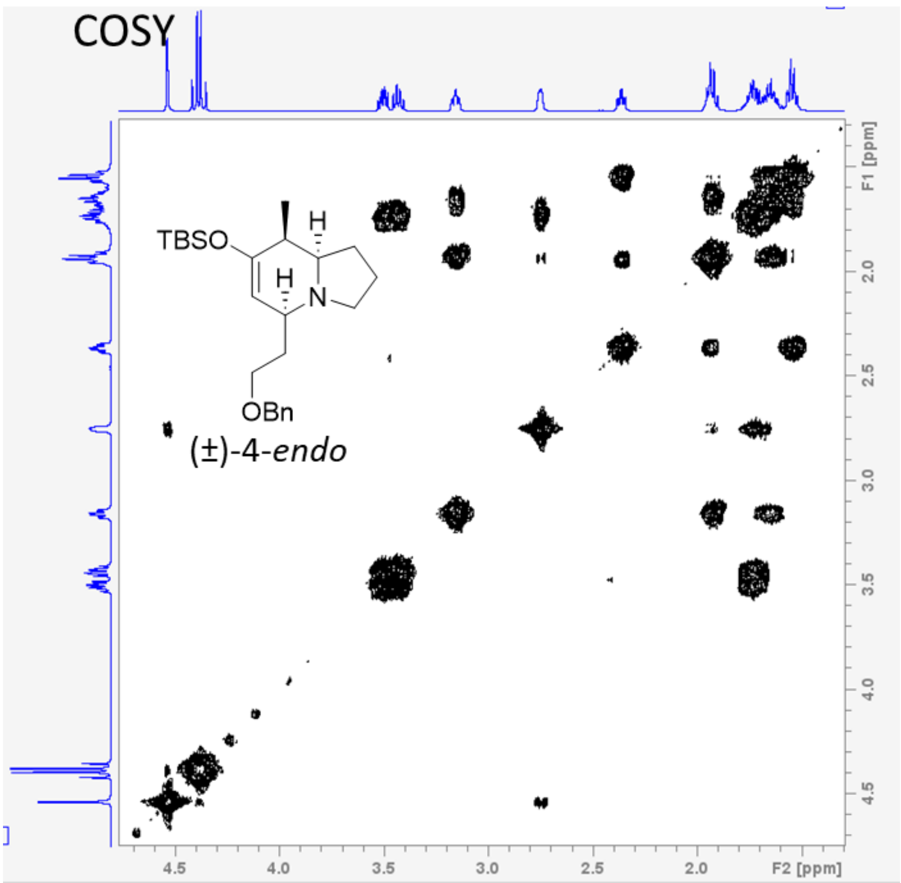
COSY
TBSO
H
H
N
OBn
(±)-4-*endo*
4.5
4.0
3.5
3.0
2.5
2.0
F2 [ppm]
F1 [ppm]

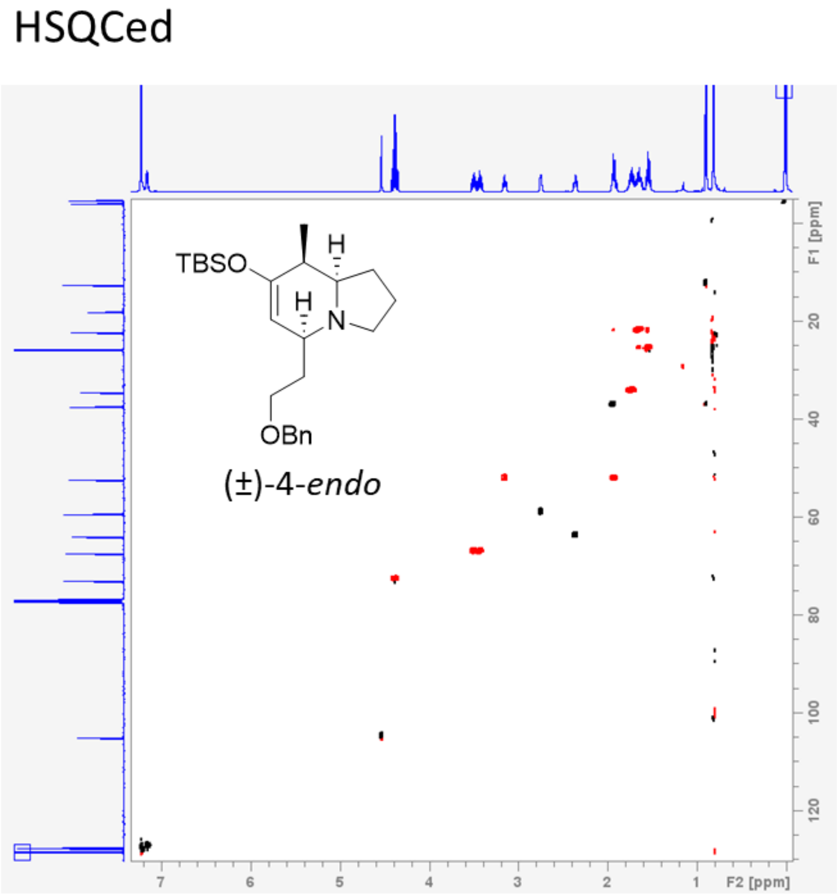
HSQCed
TBSO
H
H
N
OBn
(±)-4-*endo*
7
6
5
4
3
2
1
F2 [ppm]
F1 [ppm]
20
40
60
80
100
120

ROESY

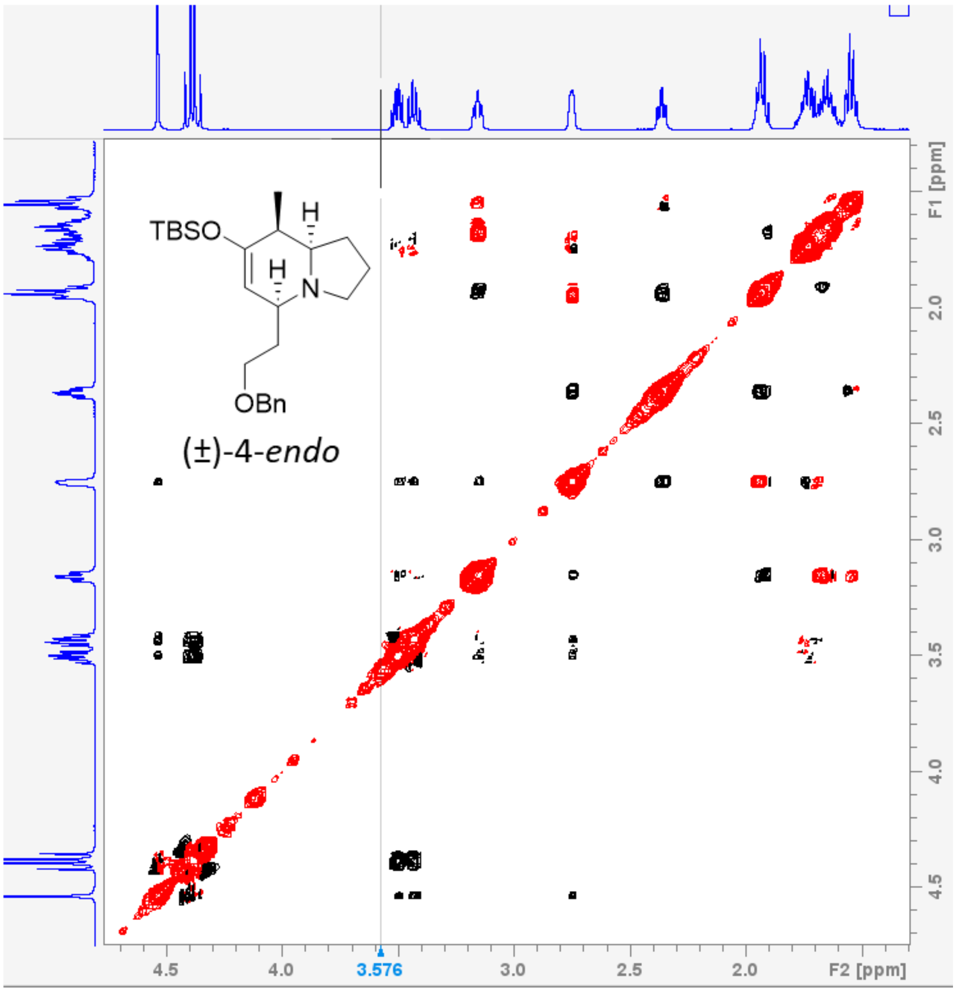

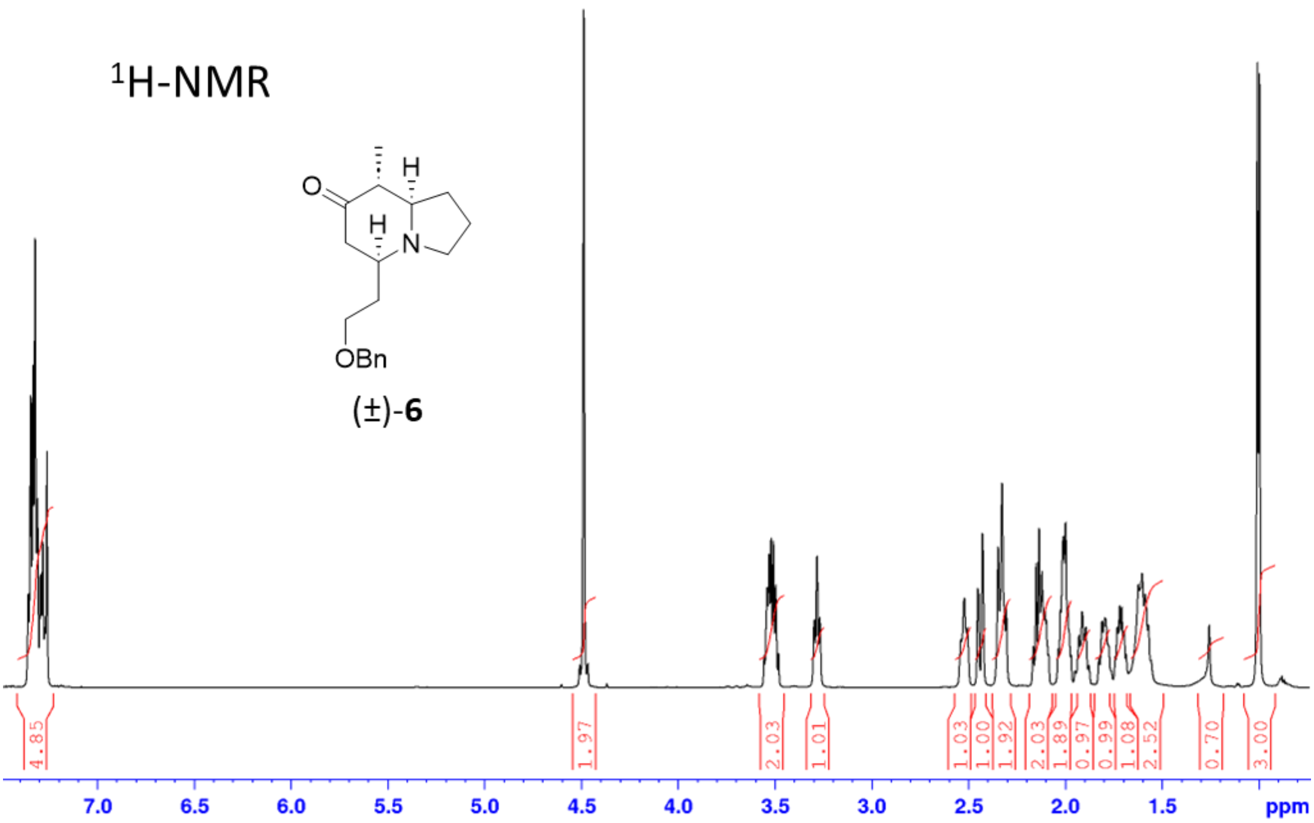

^{1}H-NMR
O
H
H
N
OBn
(±)-6
4.85
1.97
2.03
1.01
1.03
1.00
1.92
2.03
1.89
0.97
0.99
1.08
2.52
0.70
3.00
7.0
6.5
6.0
5.5
5.0
4.5
4.0
3.5
3.0
2.5
2.0
1.5
ppm

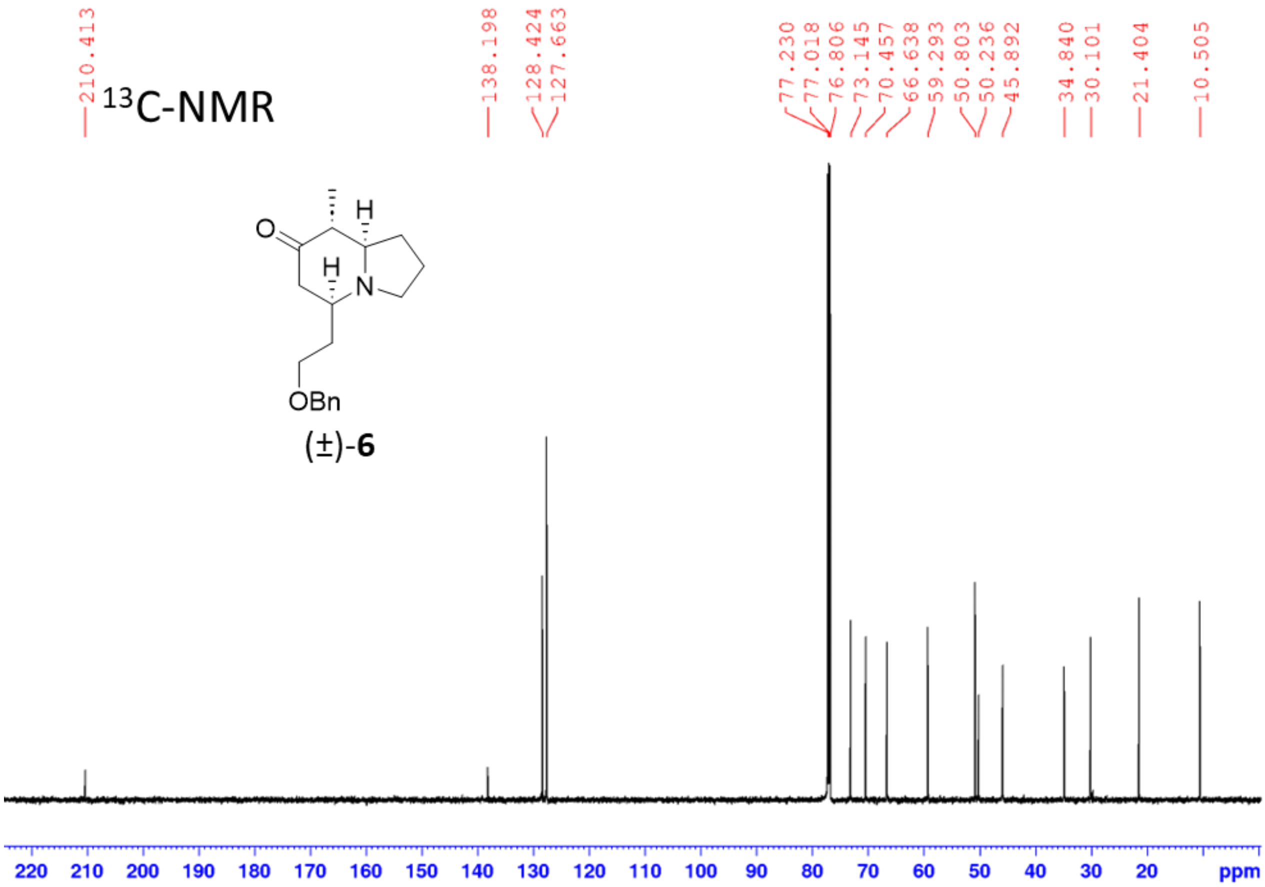

210.413
138.198
128.424
127.663
77.230
77.018
76.806
73.145
70.457
66.638
59.293
50.803
50.236
45.892
34.840
30.101
21.404
10.505
^{13}C-NMR
O
H
H
N
OBn
(±)-6
220
210
200
190
180
170
160
150
140
130
120
110
100
90
80
70
60
50
40
30
20
ppm

COSY

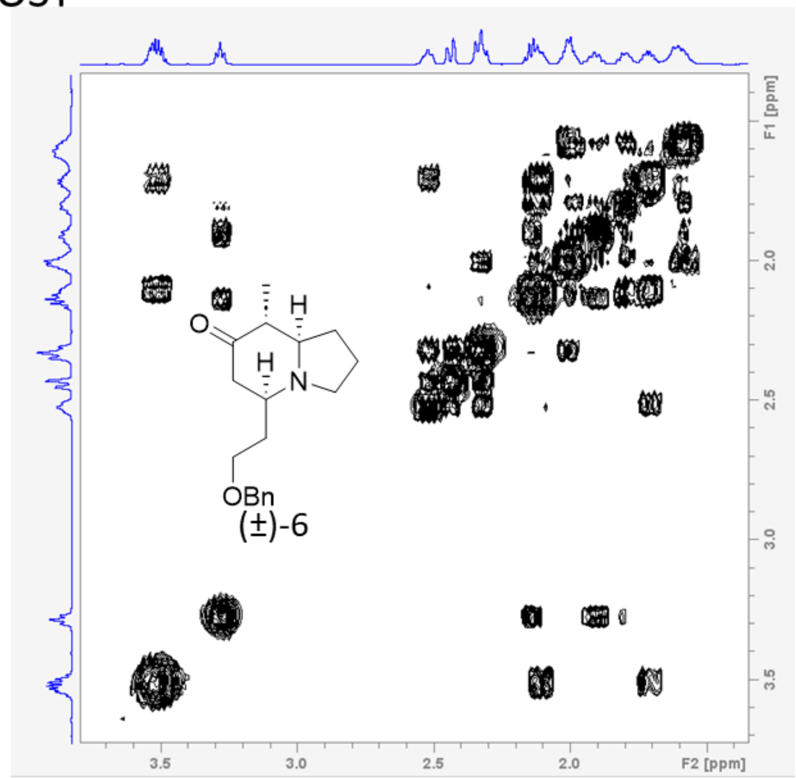

HSQCed

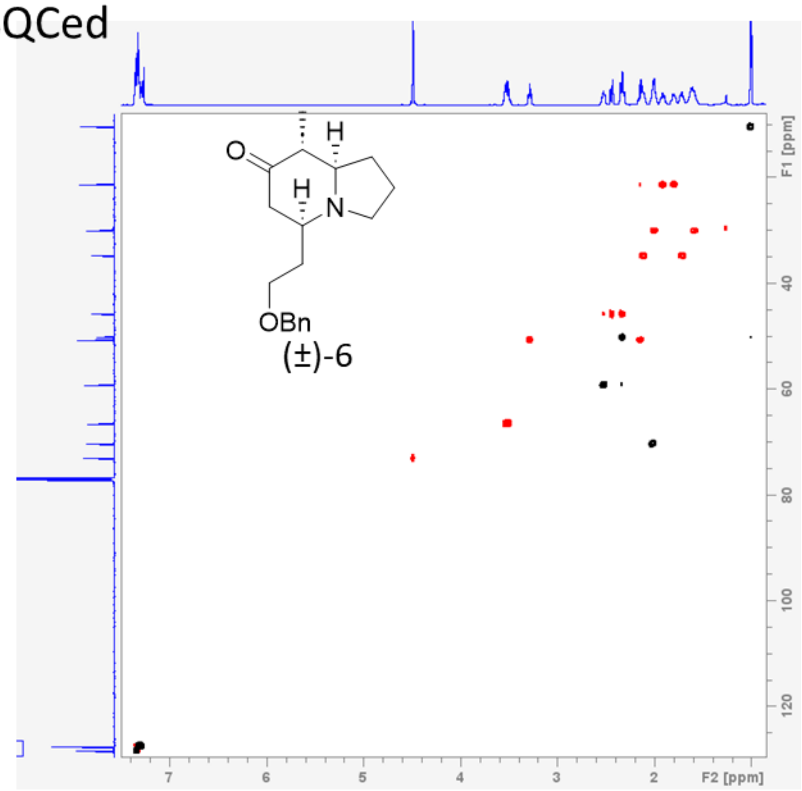

goe

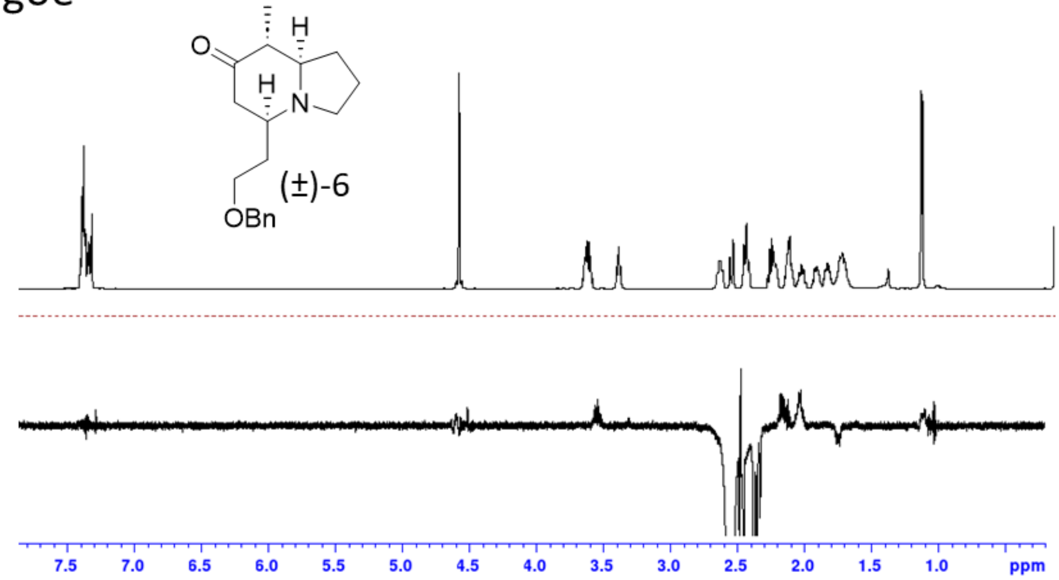
O
H
H
N
OBn
(±)-6
7.5 7.0 6.5 6.0 5.5 5.0 4.5 4.0 3.5 3.0 2.5 2.0 1.5 1.0 ppm

goe

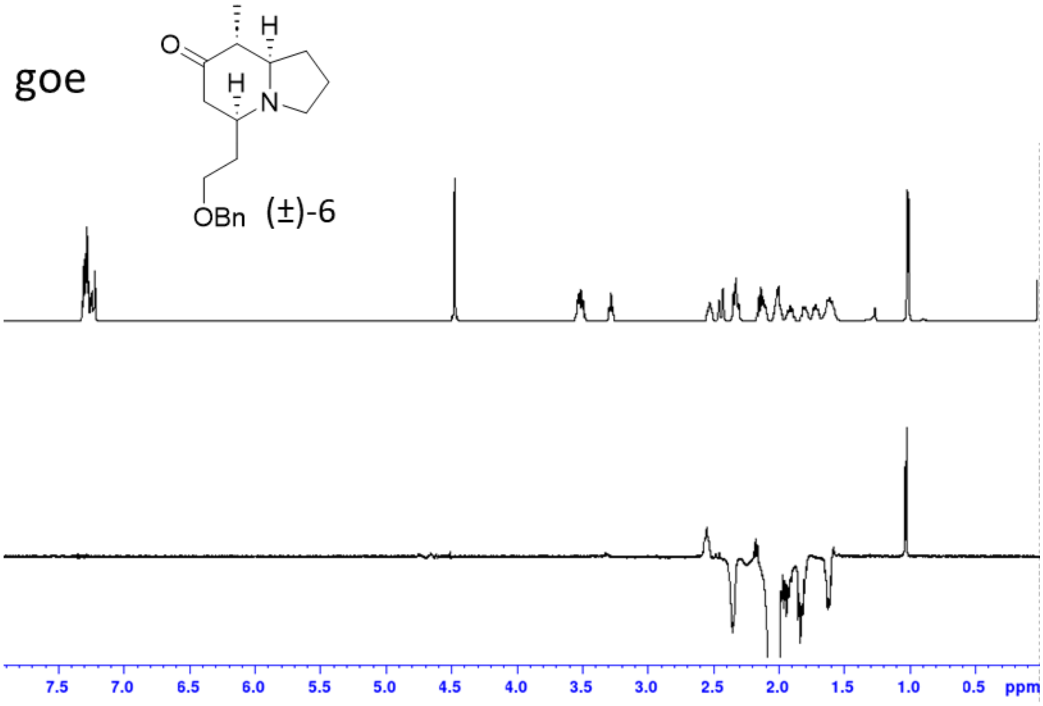
O
H
H
N
OBn
(±)-6
7.5 7.0 6.5 6.0 5.5 5.0 4.5 4.0 3.5 3.0 2.5 2.0 1.5 1.0 0.5 ppm

^{1}H-NMR

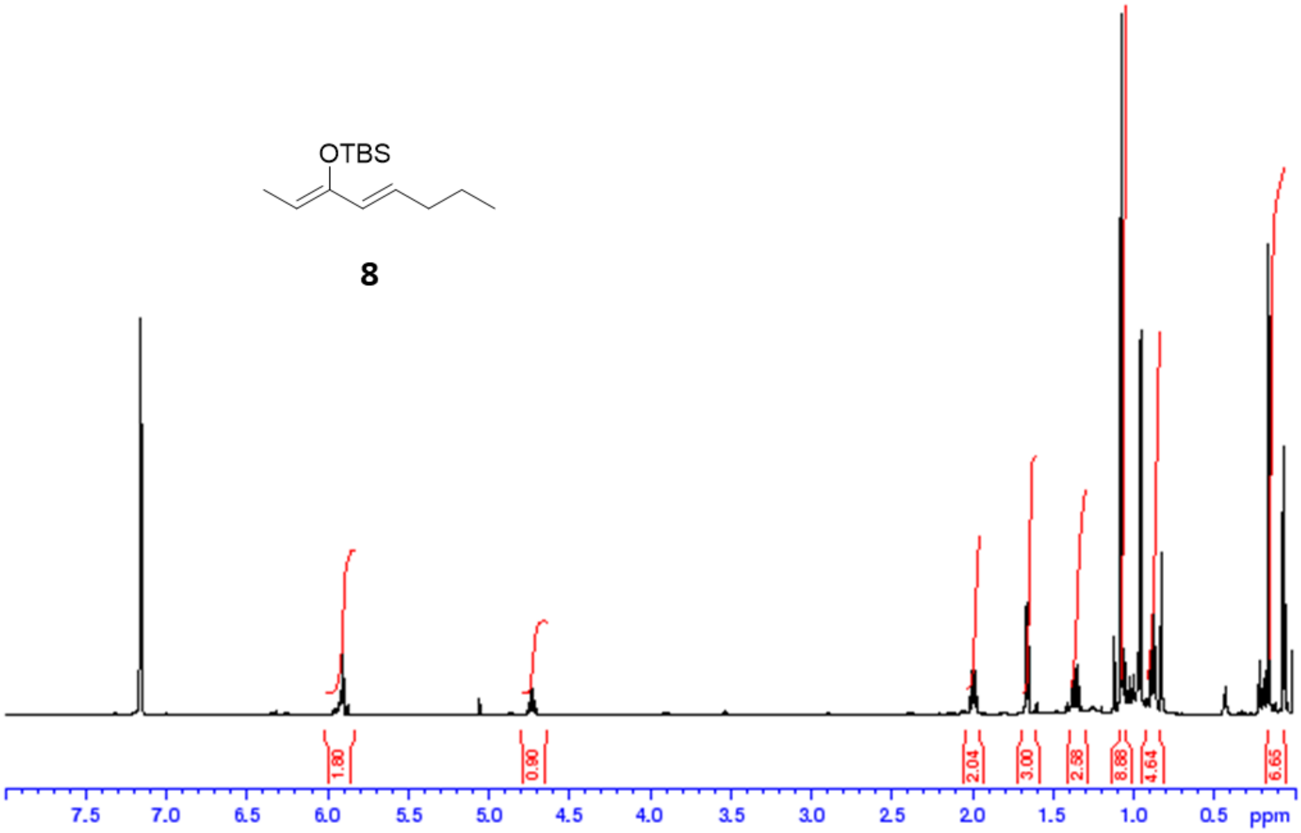

^{13}C-NMR

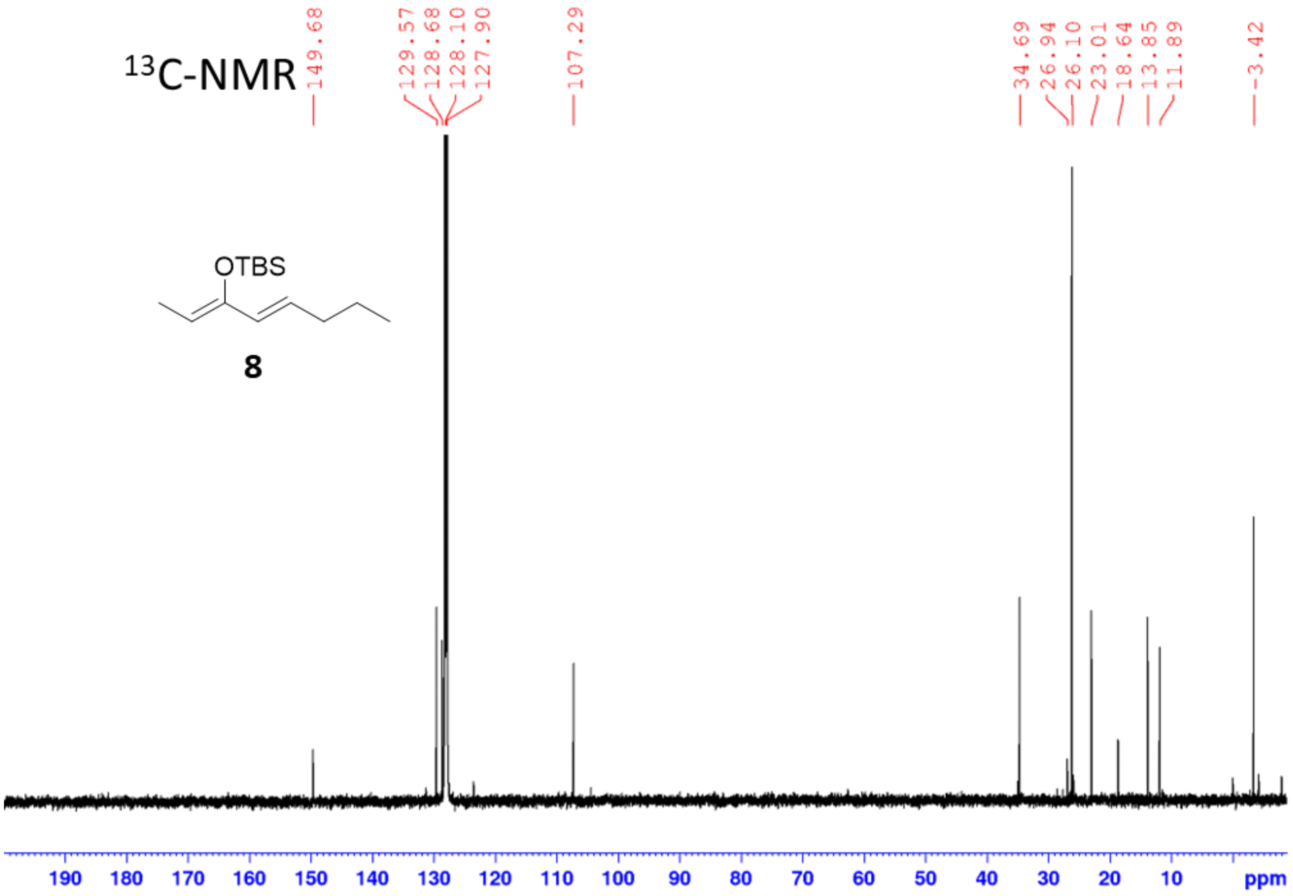

^{1}H-NMR

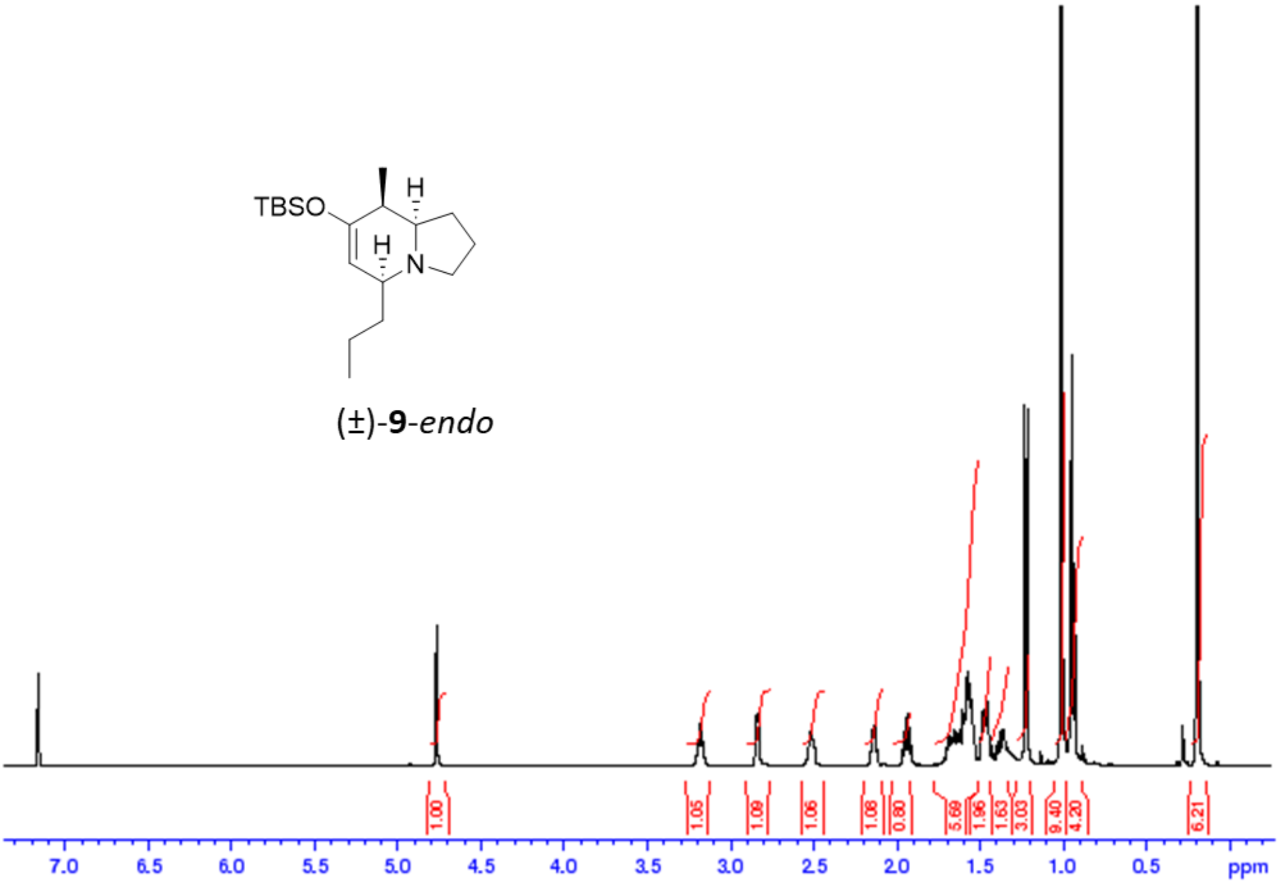

^{13}C-NMR

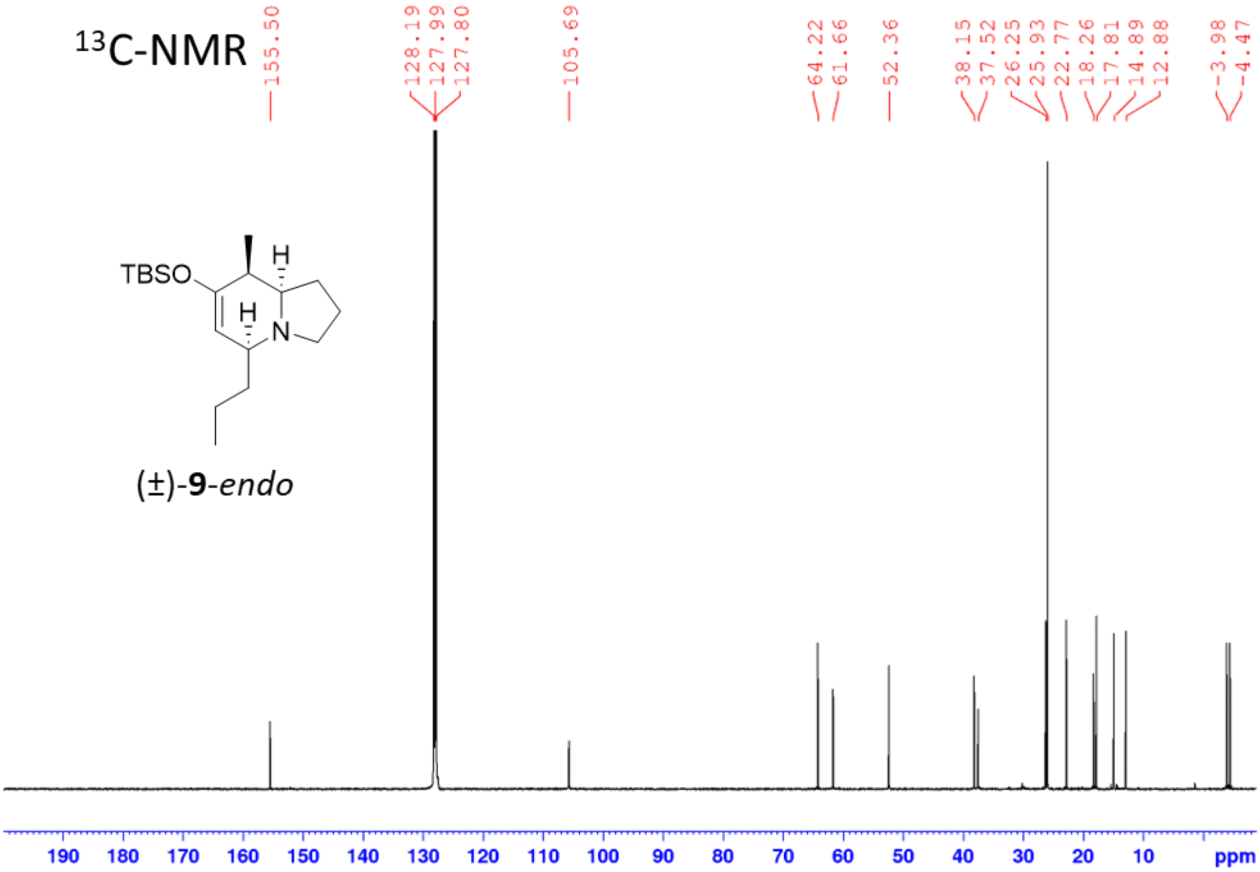

COSY

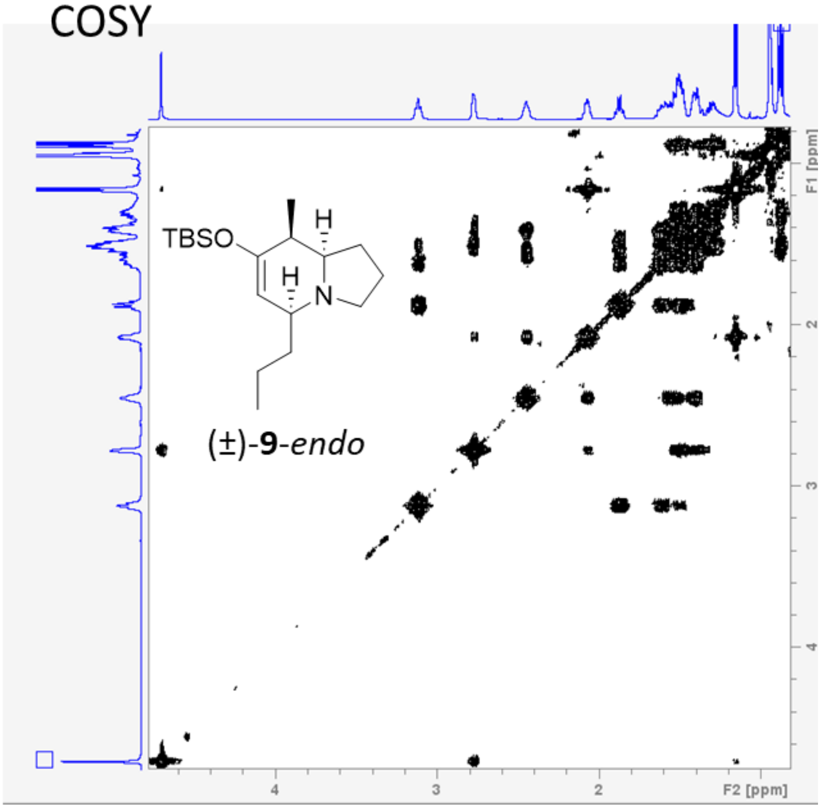

HSQCed

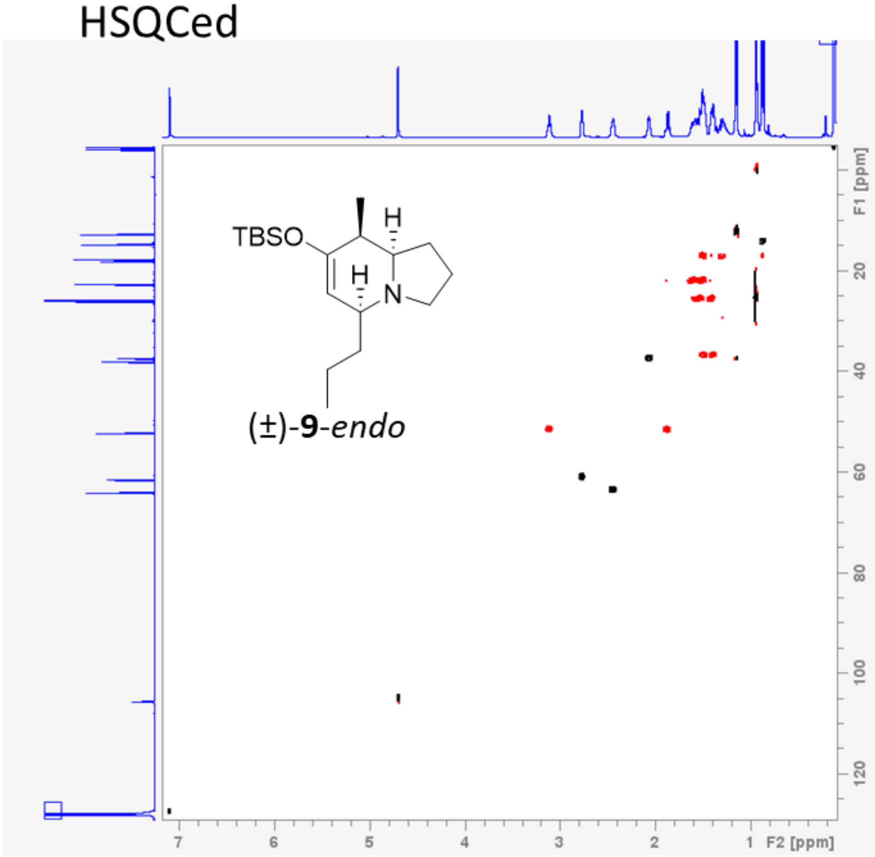

ROESY

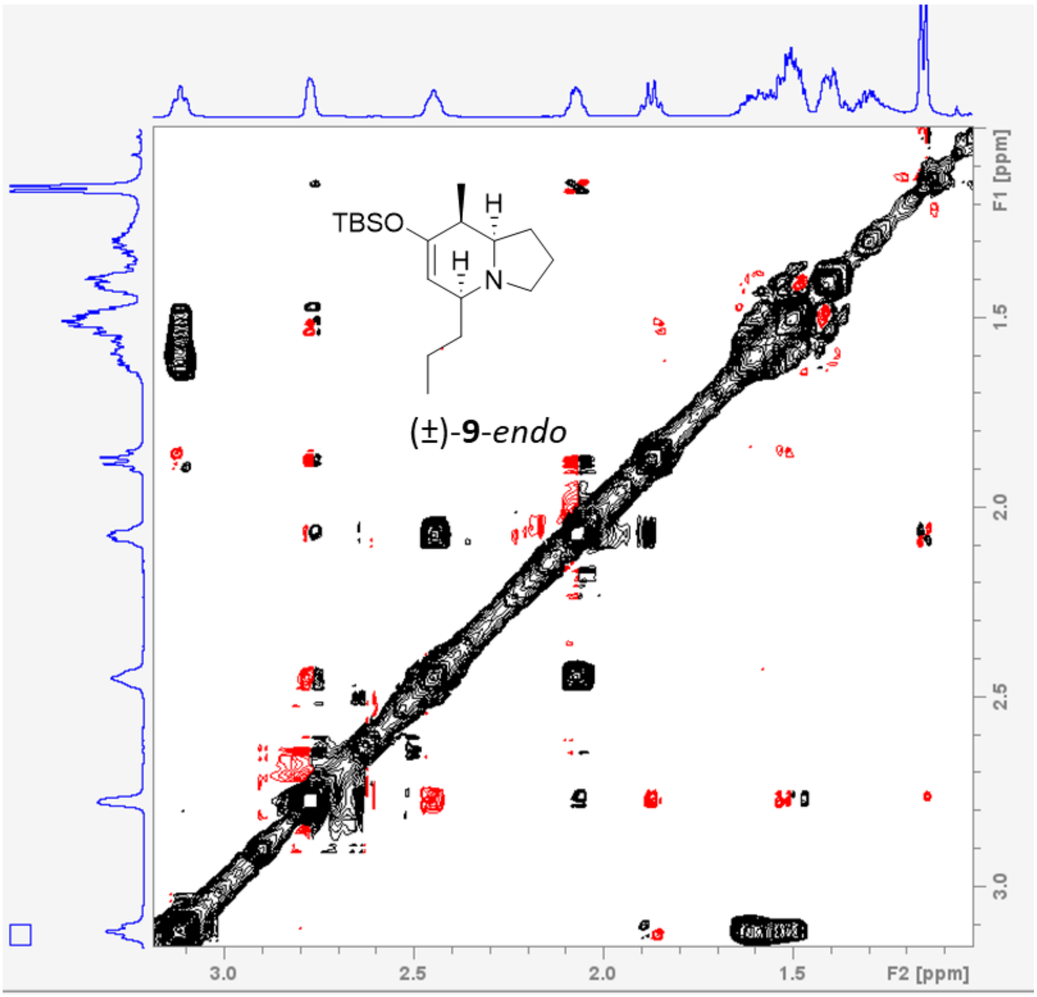

^{1}H-NMR

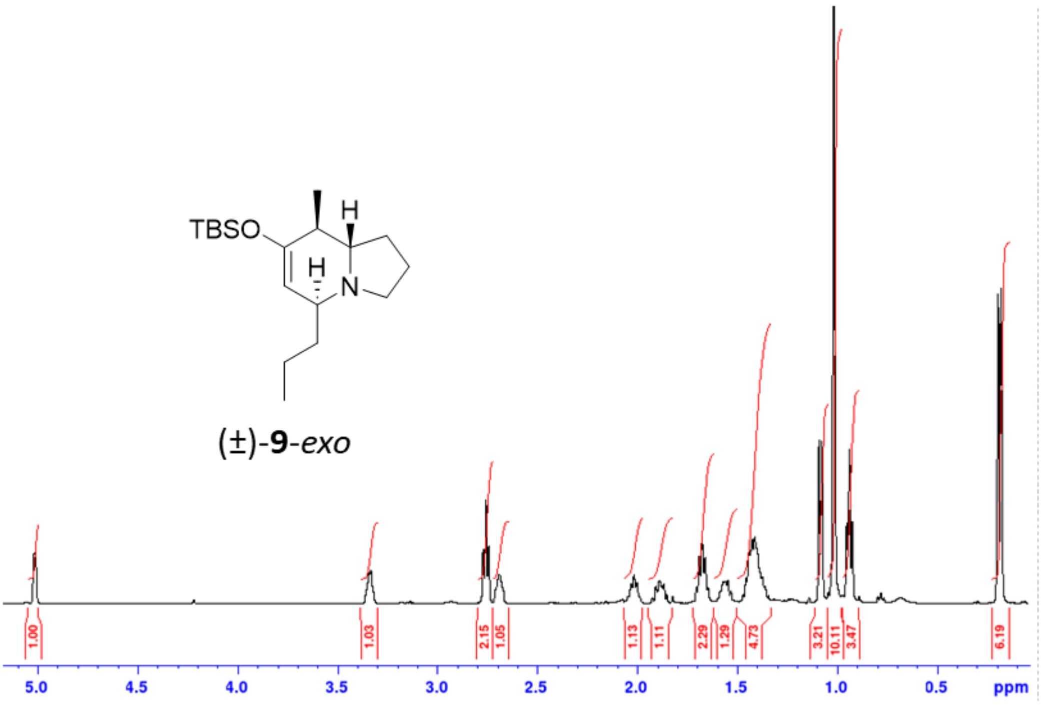

^{13}C-NMR

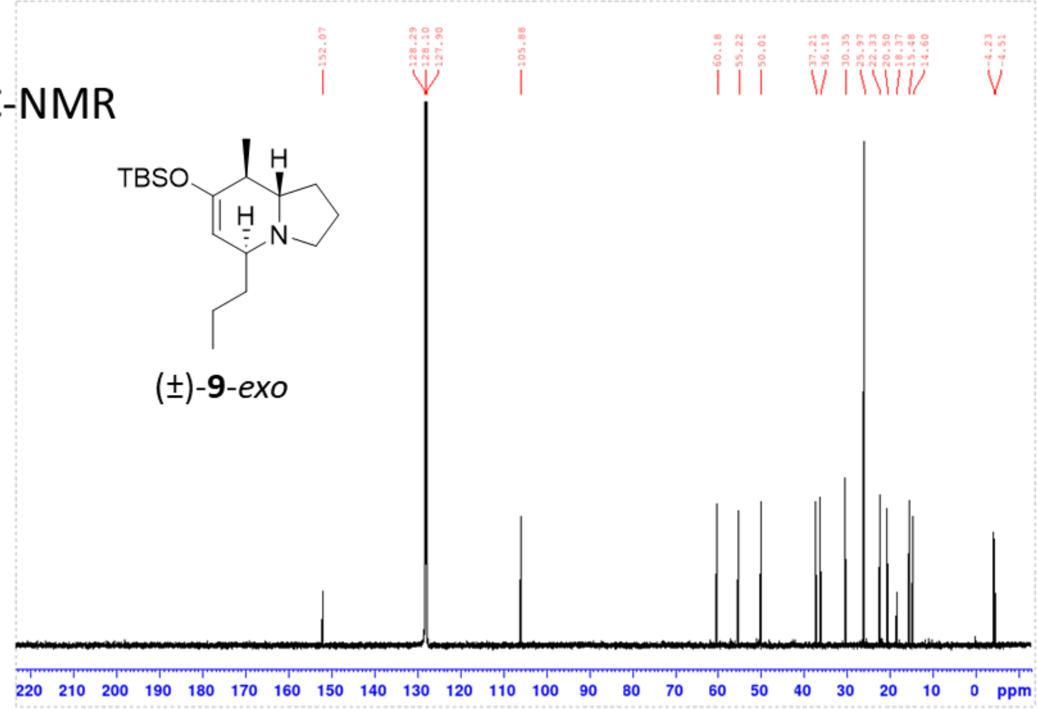

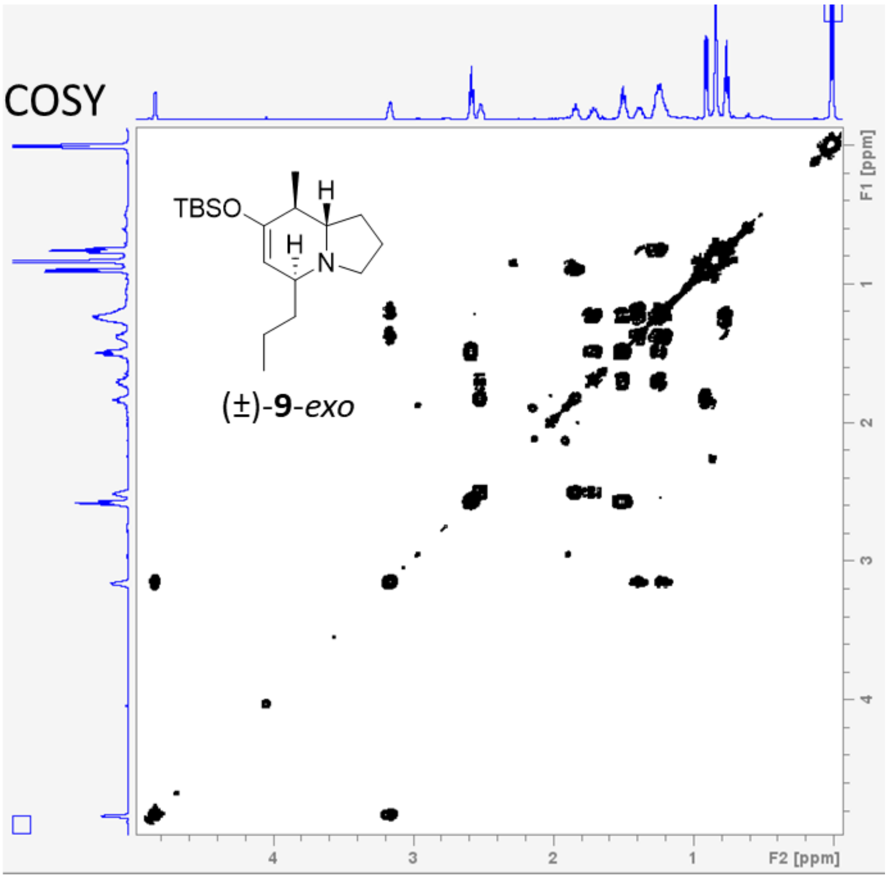
COSY
TBSO
H
H
N
(±)-9-exo
F1 [ppm]
F2 [ppm]

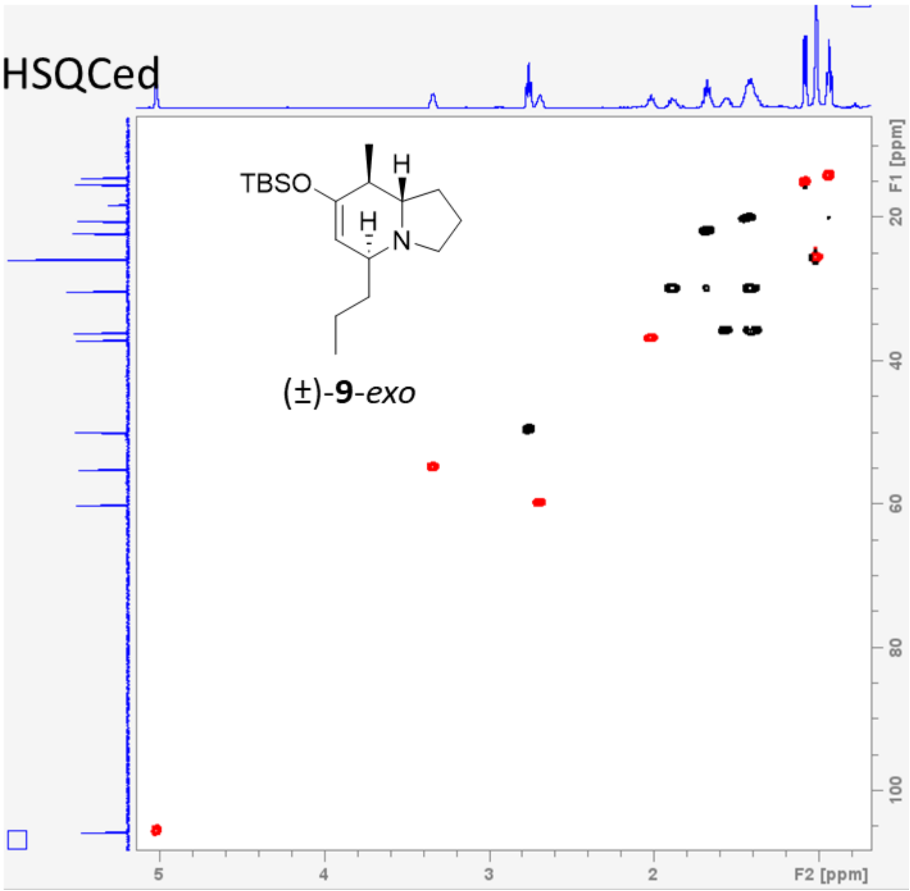
HSQCed
TBSO
H
H
N
(±)-9-exo
F1 [ppm]
F2 [ppm]

ROESY

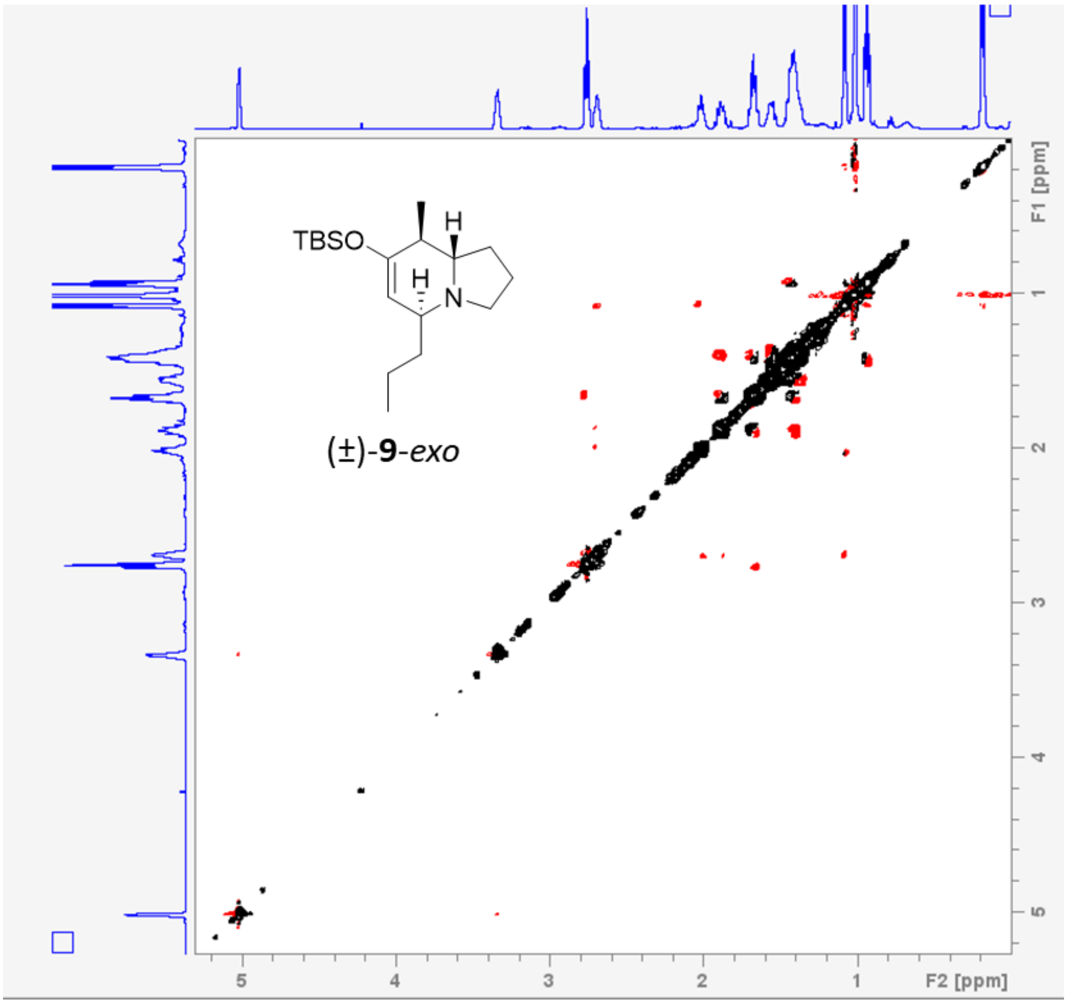

^{1}H-NMR

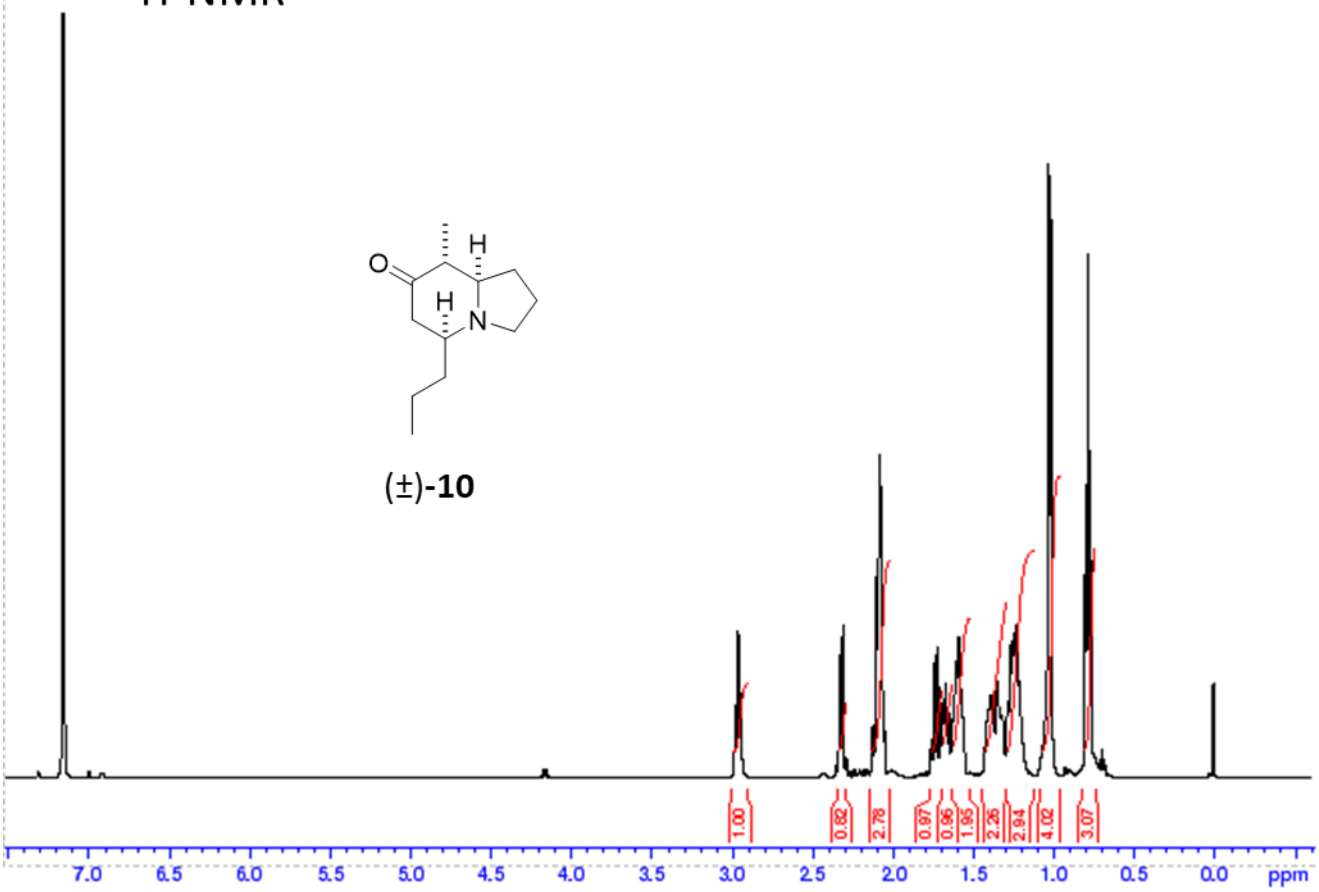

^{13}C-NMR

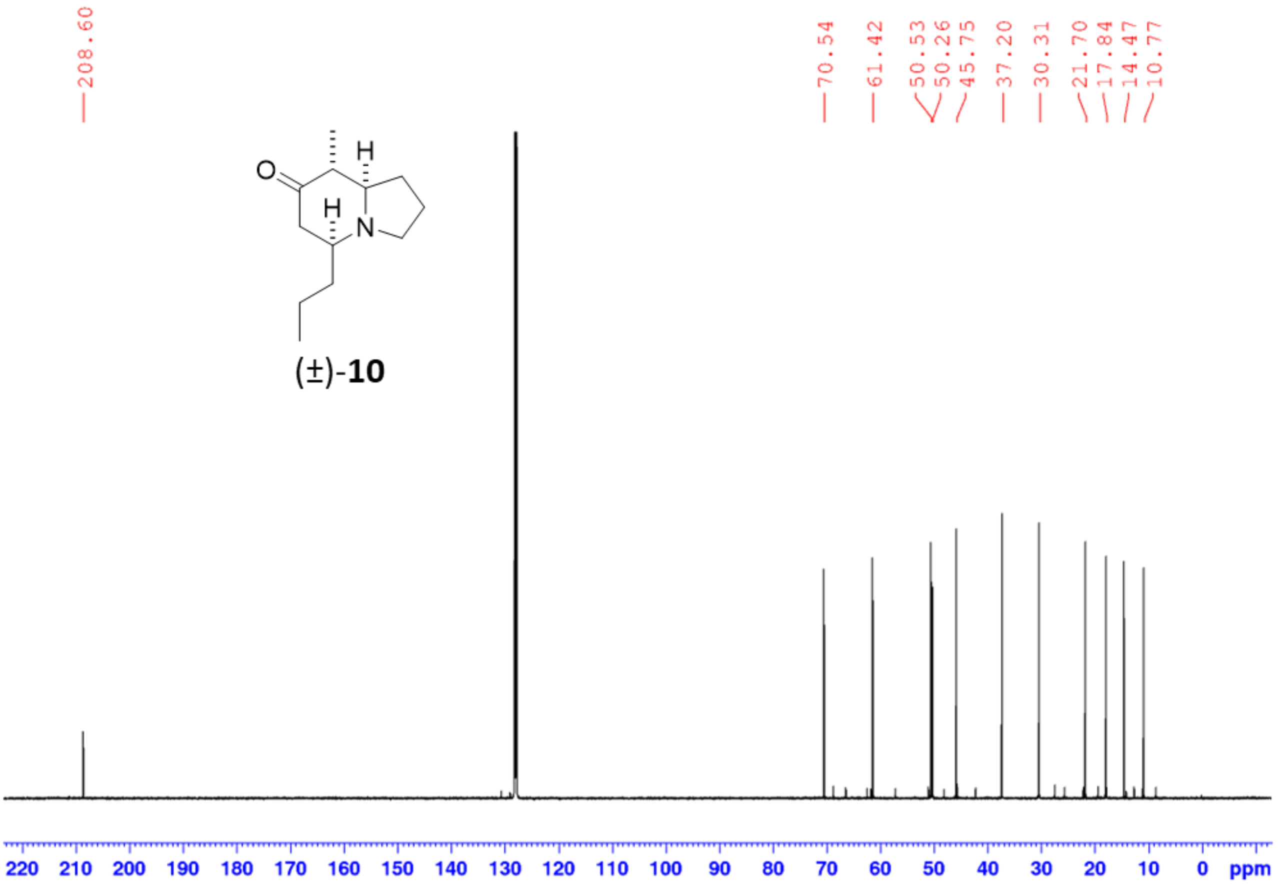

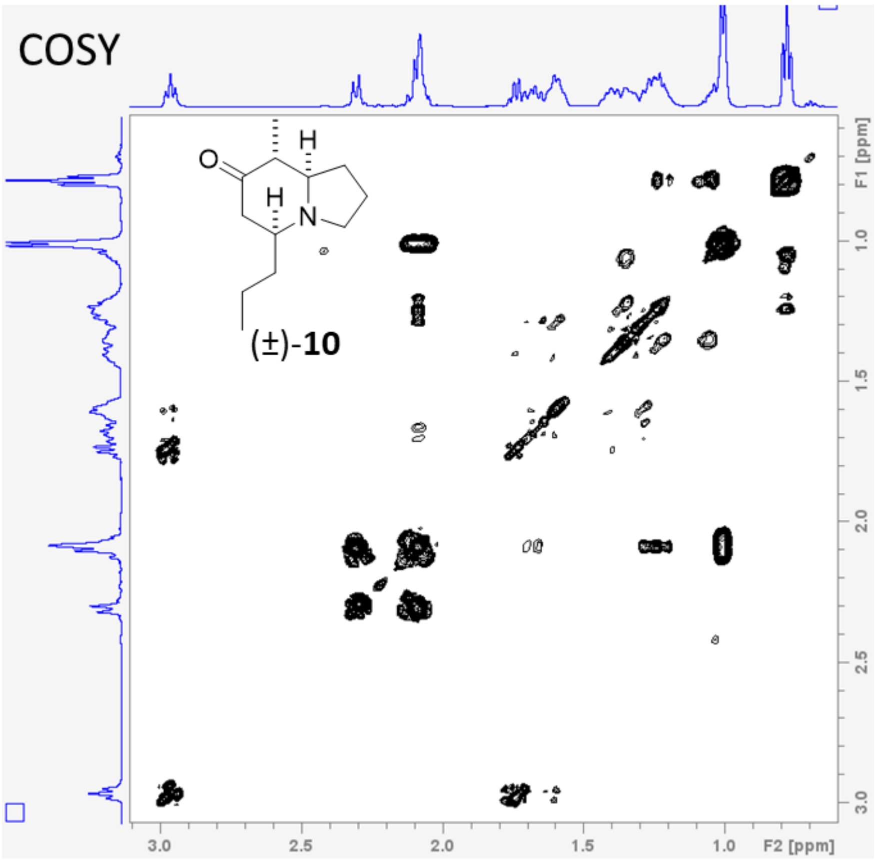
COSY
(±)-10
F1 [ppm]
F2 [ppm]
3.0
2.5
2.0
1.5
1.0

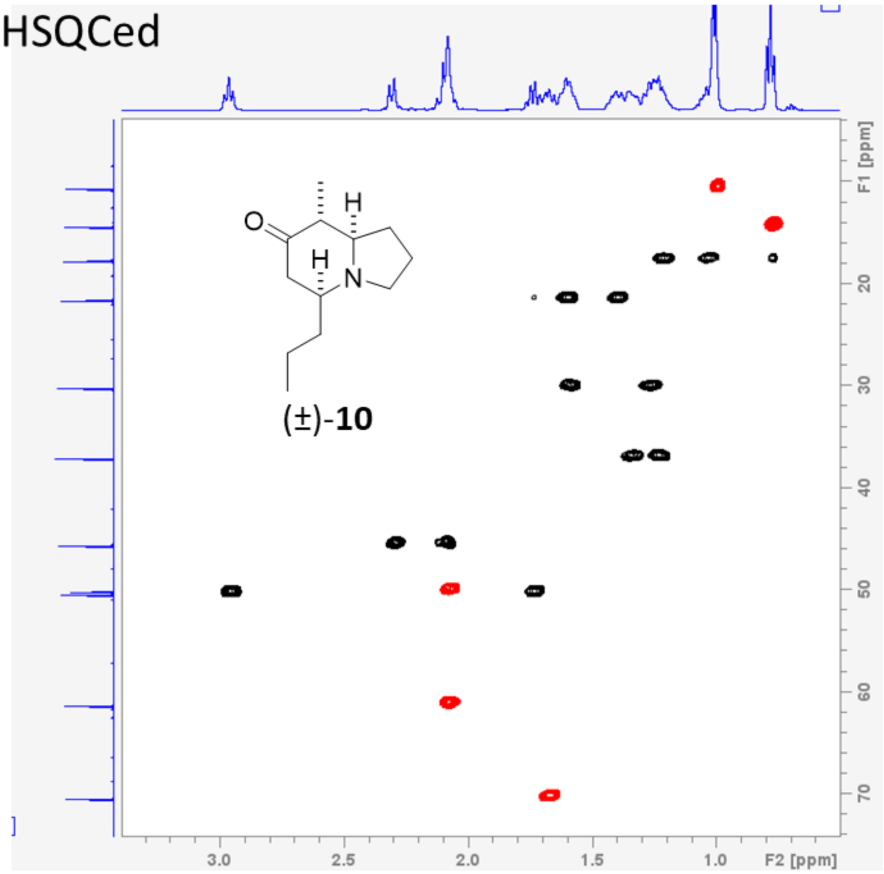
HSQCed
(±)-10
F1 [ppm]
F2 [ppm]
20
30
40
50
60
70
3.0
2.5
2.0
1.5
1.0

ROESY

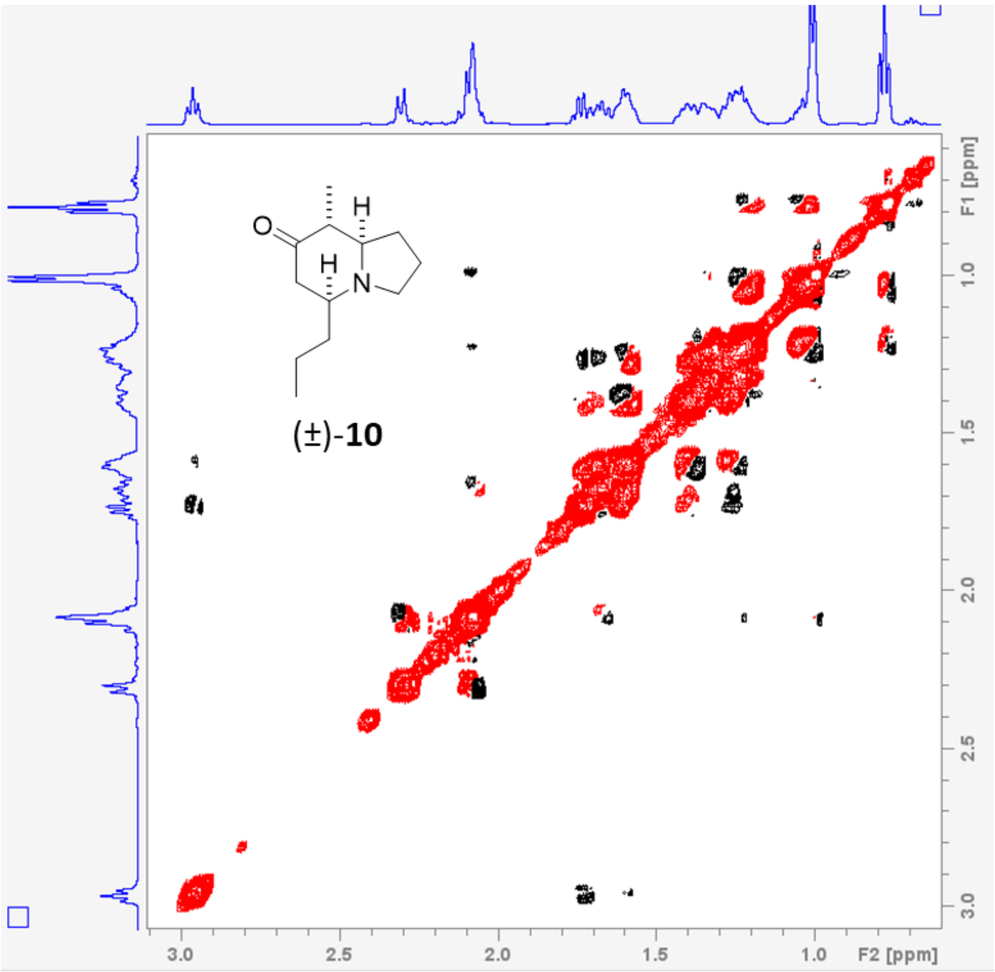

1H-NMR

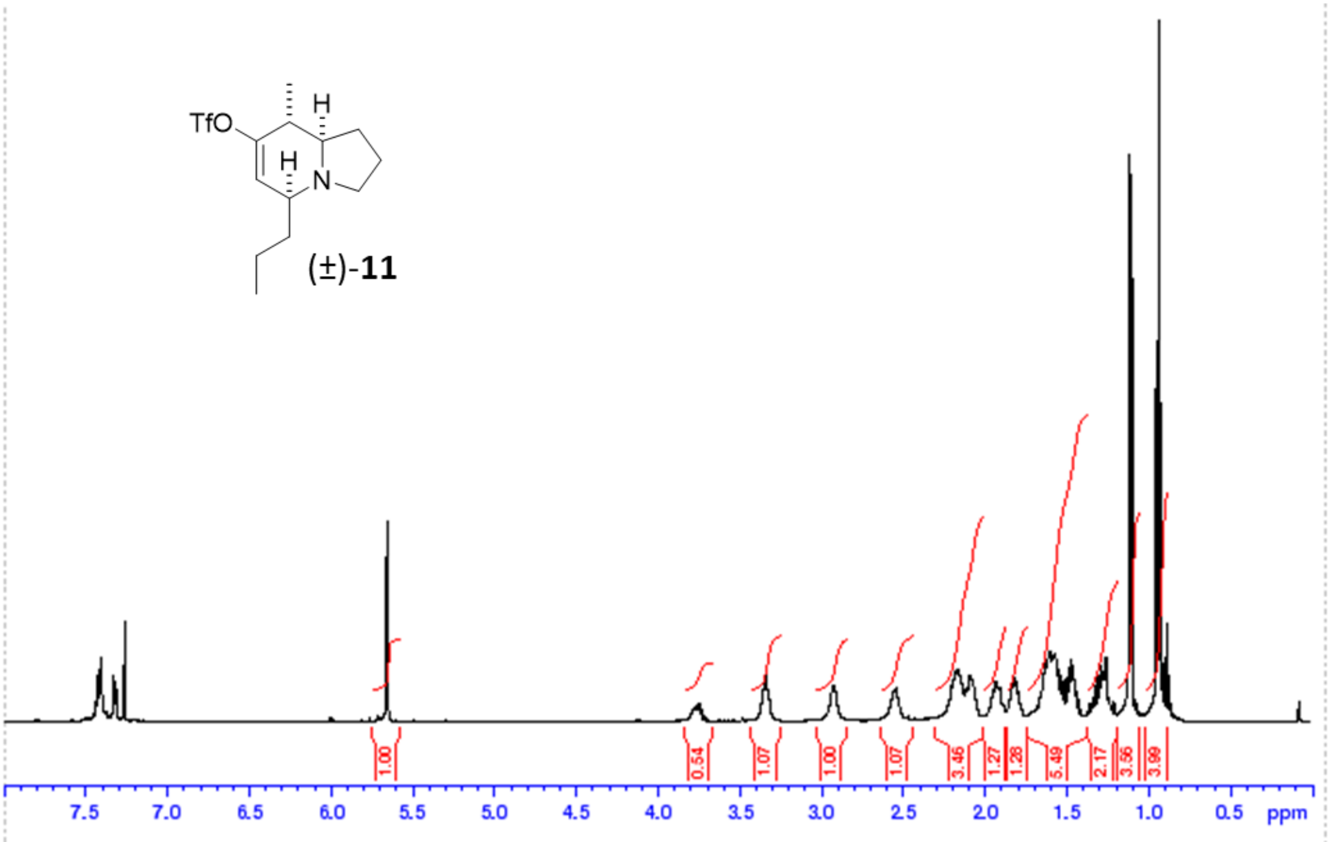

TfO
H
H
N
1.00
0.54
1.07
1.00
1.07
3.46
1.27
1.28
5.49
2.17
3.56
3.99
7.5
7.0
6.5
6.0
5.5
5.0
4.5
4.0
3.5
3.0
2.5
2.0
1.5
1.0
0.5
ppm

(±)-**11**

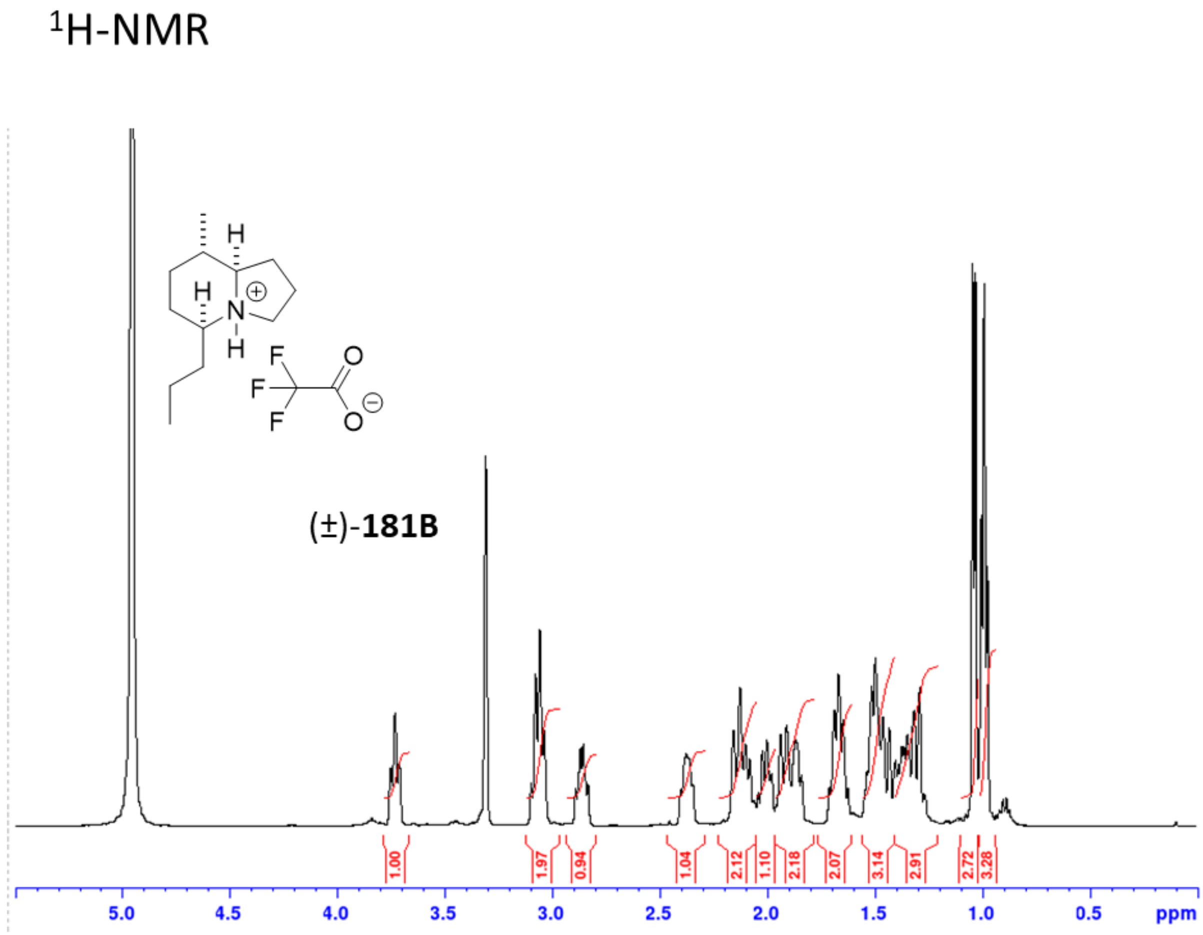

^{1}H-NMR
(±)-181B
1.00
1.97
0.94
1.04
2.12
1.10
2.18
2.07
3.14
2.91
2.72
3.28
5.0
4.5
4.0
3.5
3.0
2.5
2.0
1.5
1.0
0.5
ppm

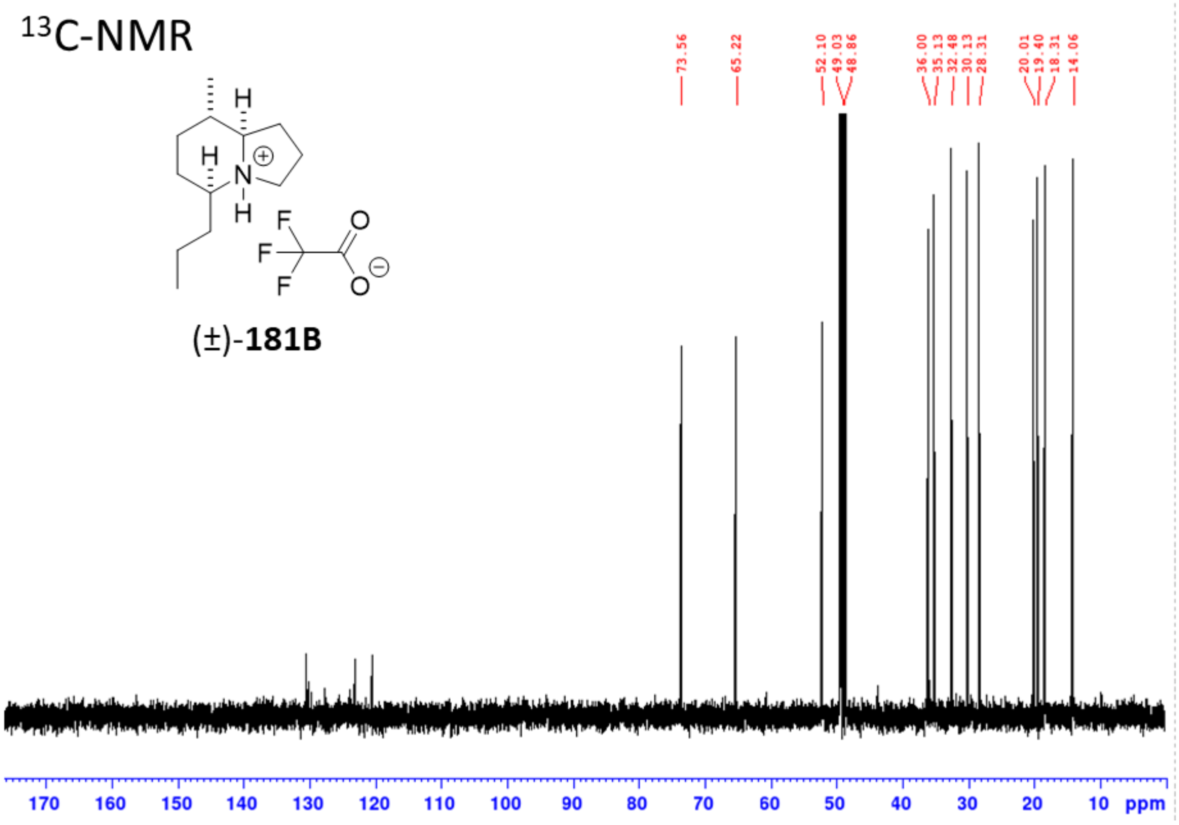

^{13}C-NMR
(±)-181B
73.56
65.22
52.10
49.03
48.86
36.00
35.13
32.48
30.13
28.31
20.01
19.40
18.31
14.06
170
160
150
140
130
120
110
100
90
80
70
60
50
40
30
20
10
ppm

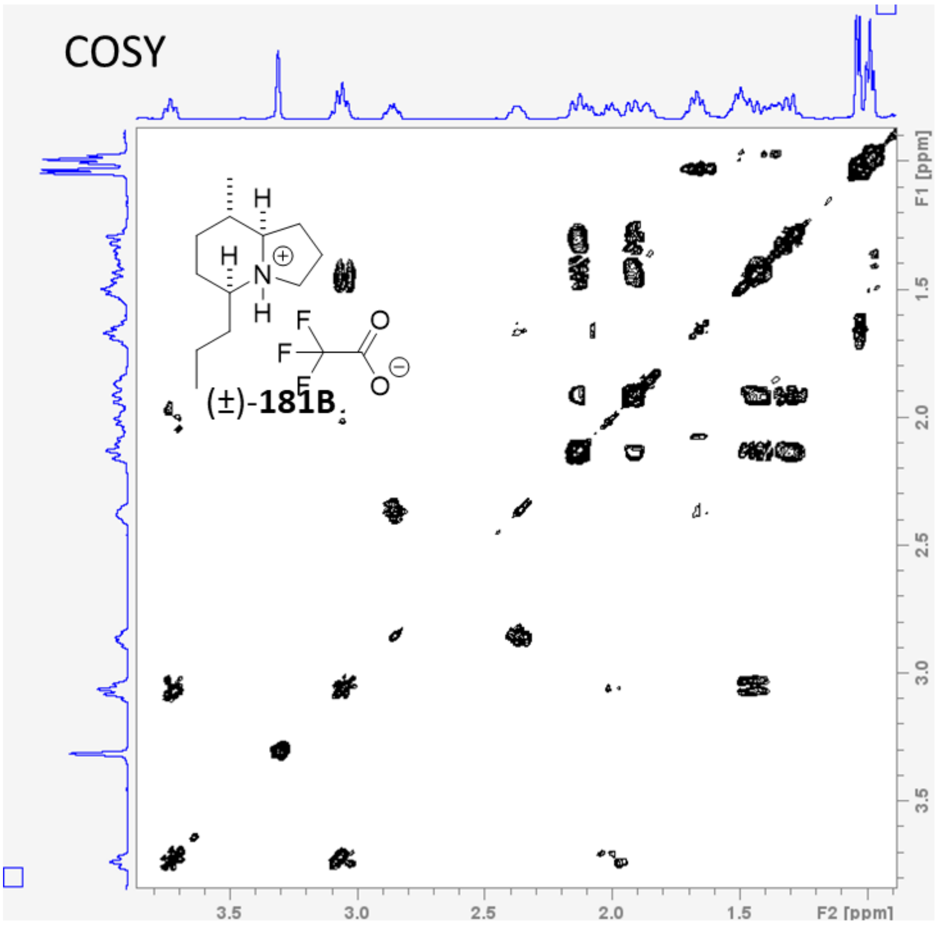

COSY
H
H
N
H
F
F
F
O
O
(±)-181B
F1 [ppm]
1.5
2.0
2.5
3.0
3.5
3.5
3.0
2.5
2.0
1.5
F2 [ppm]

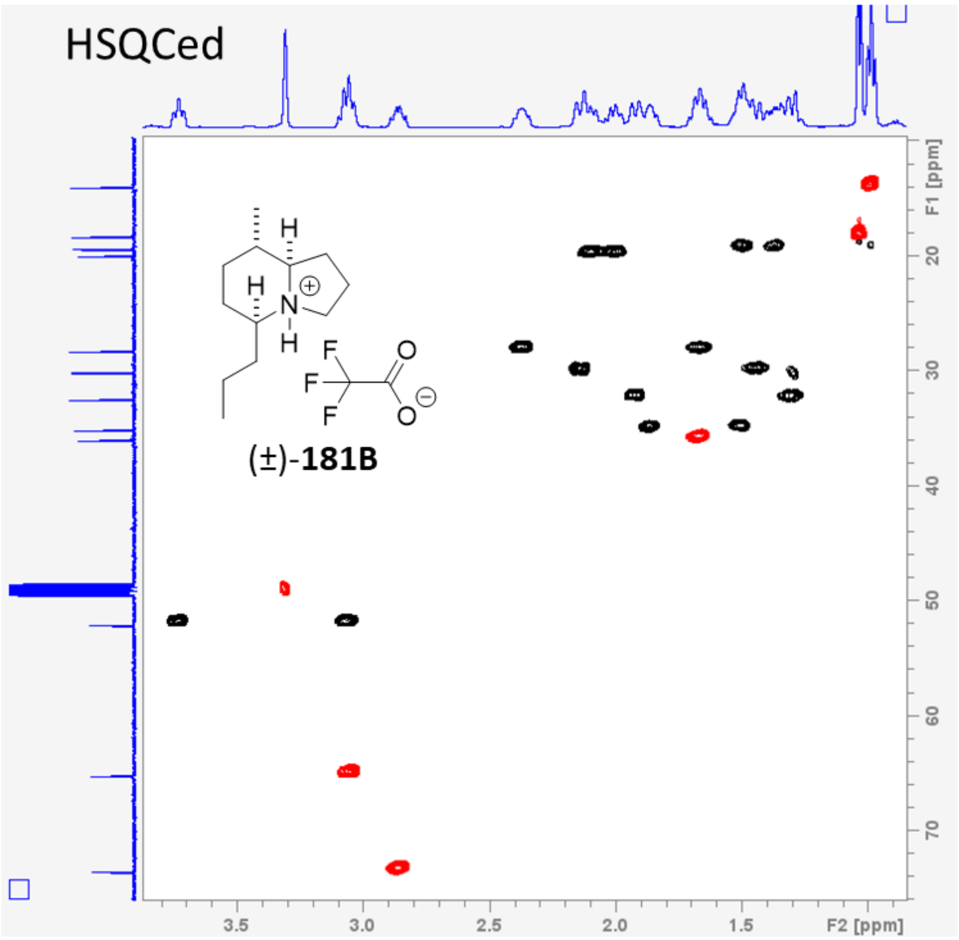

HSQCed
H
H
N
H
F
F
F
O
O
(±)-181B
F1 [ppm]
20
30
40
50
60
70
3.5
3.0
2.5
2.0
1.5
F2 [ppm]

ROESY

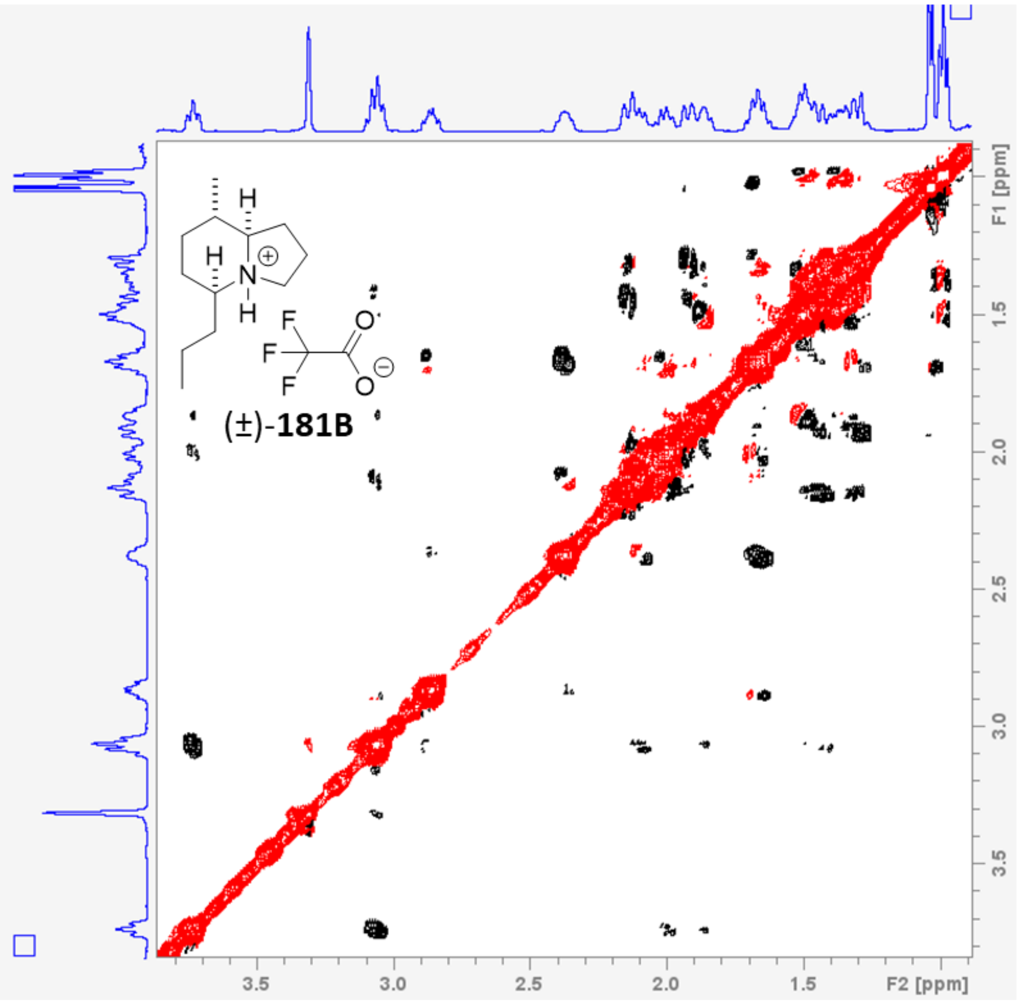

References

30. Neese, F. The ORCA program system. *WIREs Comput. Mol. Sci.* **2012**, *2*, 73–78. https://doi.org/10.1002/wcms.81.

39. Hanwell, M.D.; Curtis, D.E.; Lonie, D.C.; Vandermeerschd, T.; Zurek, E.; Hutchison, G.R. Avogadro: An Advanced Semantic Chemical Editor, Visualization, and Analysis Platform. *J. Cheminform.* **2012**, *4*, 17. https://doi.org/10.1186/1758-2946-4-17.

40. Legault, C.Y. *CYLview20*; Université de Sherbrooke: Sherbrooke, QC, USA, 2020. Available online: http://www.cylview.org (accessed on 6 October 2023).
